# Ten new aurovertins from cultures of the basidiomycete *Albatrellus confluens*

**DOI:** 10.1007/s13659-012-0088-y

**Published:** 2013-01-12

**Authors:** Hua Guo, Tao Feng, Zheng-Hui Li, Ji-Kai Liu

**Affiliations:** 188 State Key Laboratory of Phytochemistry and Plant Resources in West China, Kunming Institute of Botany, Chinese Academy of Sciences, Kunming, 650201 China; 288 School of Chemistry and Life Science, Anshan Normal College, Anshan, 114005 China; 388 University of Chinese Academy of Sciences, Beijing, 100049 China

**Keywords:** *Albatrellus confluens*, aurovertins, cytotoxic activities

## Abstract

Aurovertins J-S (**1–10**), together with four known metabolites, aurovertins B, C, E, and I (**11–14**), were isolated from cultures of the basidiomycete *Albatrellus confluens*. The structures of compounds **1–10** were elucidated on the basis of extensive spectroscopic analysis. All compounds were evaluated for their cytotoxic activities on five tumor cell lines.

**Electronic Supplementary Material:**

Supplementary material is available for this article at 10.1007/s13659-012-0088-y and is accessible for authorized users.

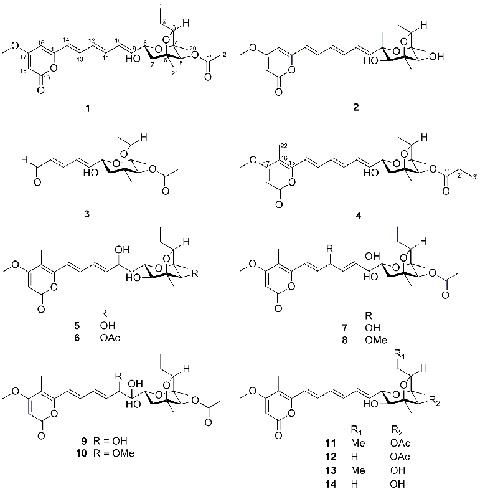

## Introduction

The first four aurovertin-type compounds, aurovertins A–D, were isolated as toxic substances from fermentation broth of the fungus *Calcarisporium arbuscula*.[1] Aurovertins are a class of compounds with the basic structure of 2,6-dioxabicyclo[3.2.1]octane ring system and a conjugated *α*-pyrone moiety originated from polyketide pathway. They have attracted considerable attention of chemists because of their actitivities as potent inhibitors of ATP synthesis and antitumor agents.[2–7] There have been nine aurovertins (A–I) structurally elucidated to date,[1,8–10] while the relative and absolute configurations of aurovertin B were determined by spectroscopic analysis and total synthesis, respectively.[11,12] The absolute configuration of aurovertin F was determined by analysis of the CD spectrum recently.[9] It is deduced that aurovertin-type of compounds obtained in literature probably share the same absolute configuration on biosynthetic grounds.[10,13] During our continuing investigation of bioactive microbial secondary metabolites from the fungus,[8,14–23] ten new aurovertin derivatives, named as aurovertins J–S (**1**–**10**), along with four previously reported aurovertins B, C, E and I (**11**–**14**), were isolated from cultures of the basidiomycete *Albatrellus confluens* (Alb. et Schw.: Fr.) Kolt. et Pouz. All compounds were evaluated for their cytotoxicities against five human cancer cell lines. This paper deals with the isolation, structural elucidation, and bioactivities of these isolates.

**Figure 1.** Structures of compounds **1**–**14**

## Results and Discussion

A total of 14 aurovertins including 10 previously-unreported compounds were isolated from cultures of the basidiomycete *Albatrellus confluens*. Comparison with all the reported nine aurovertin-type metabolites to date, it should be noticed that the methyl (C-22) in the moiety of *α*-pyrone in aurovertins was missing in aurovertin J (**1**) and aurovertin K (**2**), the moiety of

*To whom correspondence should be addressed. E-mail: jkliu@mail.kib.ac.cn

*α*-pyrone was degraded in aurovertin L (**3**) and the polyene side chain was partly oxidized in aurovertins J–S (**5**–**10**). In addition, compounds **7** and **11** exhibited certain cytotoxicities.

Aurovertin J (**1**) was obtained as yellow syrup. The molecular formula of **1** was determined to be $C_{24}H_{30}O_8$ on the basis of HREIMS *m*/*z* 446.1934 (calcd for 446.1941), corresponding to ten degrees of unsaturation. The IR spectrum showed absorption bands for hydroxy group (3441 $cm^{-1}$) and double bonds (1632 $cm^{-1}$). The $^{13}C$ NMR and DEPT spectra showed 24 carbon signals that attributed to five methyls, one methylene, twelve methines, and six quaternary carbons. The $^{1}H$ and $^{13}C$ NMR spectra (Tables 1 and 3) of **1** were similar to those of aurovertin B (**11**)[5] except for the loss of the methyl group at C-16. The upfield shift of C-16 ($\delta_C$ 101.0, d) clearly indicated that **1** was a demethylated analogue of **11**, which was supported by the observed HMBC correlations from $\delta_H$ 5.84 (1H, d, *J* = 1.9 Hz, H-16) to $\delta_C$ 122.0 (d, C-14), 158.6 (s, C-15), 171.0 (s, C-17), and 88.7 (d, C-18). In the light of the evidences mentioned above and the key $^{1}H$-$^{1}H$ COSY and HMBC correlations (Figure 2), the planar structure of **1** was therefore elucidated as shown in Figure 1, named aurovertin J. The key ROESY correlations of H-2 with H-8, H-3 with Me-20, and H-5 with H-7 were observed in the ROESY spectrum of compound **1** (Figure 2), which established the relative stereochemistry of **1** as that of **11**. Because the NMR data and other physico-chemical properties of **1** are very similar to those of **11**, the absolute configuration and geometrical isomerism of both compounds are suggested to be the same. Therefore, the structure of aurovertin J (**1**) was established, as shown.

Aurovertin K (**2**) was inferred to possess the molecular formula $C_{22}H_{28}O_7$ on the basis of its HREIMS at *m*/*z* 404.1832 $[M]^+$. Preliminary analysis of the NMR data also indicated that

**Figure 2.** Key $^{1}H$-$^{1}H$ COSY, HMBC and ROESY correlations of **1**

**2** possessed a similar skeleton to that of aurovertin I (**14**).[10] The same demethylation at C-16 ($\delta_C$ 101.0) was concluded by the observed HMBC correlations from H-16 to C-14, C-15, C-17, and C-18 as the same to those of **1**. In addition, a methyl carbon at $\delta_C$ 26.5 (q) was assigned to be placed at C-8 according to the HMBC correlations from $\delta_H$ 1.26 (3H, s, H-8) to $\delta_C$ 79.6 (d, C-7), 78.2 (s, C-8), and 147.1 (d, C-9). The ROESY correlations of Me-2 with Me-8, H-3 with Me-20, and H-5 with H-7 indicated the same configuration to that of **1**. Thus, compound **2** (aurovertin K) was established, as shown.

Aurovertin L (**3**) exhibited an ion peak at *m*/*z* 324.1564 $[M]^+$ in the HREIMS, in agreement with the molecular formula $C_{17}H_{24}O_6$. Preliminary analysis of 1D NMR data suggested that compound **3** was still an aurovertin derivative, as indicated by the characteristic carbon signals for C-1 to C-8, as well as the olefinic carbons for C-9 to C-12 (Tables 1 and 3). However, compound **3** might be identified as a degraded product of **11**,[5] in which the double bond between C-13 and C-14 was cleaved, then an aldehyde group was formed at C-13 ($\delta_C$ 194.0, d), as supported by the HMBC correlation from $\delta_H$ 9.58 (1H, d, *J* = 8.0 Hz, H-13) to $\delta_C$ 151.3 (d, C-11) and 132.1 (d, C-12). Further analysis of other spectroscopic data (HSQC, HMBC, $^{1}H$-$^{1}H$ COSY, ROESY) established the structure of compound **3** (aurovertin L) as shown in Figure 1.

**Table 1. $^{1}H$ NMR spectroscopic data of aurovertins J–N (1–5) in $CDCl_3$**

| no. | **1**[a] | **2**[b] | **3**[a] | **4**[c] | **5**[c] |
|---|---|---|---|---|---|
| 1 | 1.09 (3H, t, 7.5) | | 1.09 (3H, t, 7.5) | 1.08 (3H, t, 7.6) | 1.00 (3H, t, 7.5) |
| 2 | 1.70 (2H, m) | 1.19 (3H, d, 6.5) | 1.65 (2H, br. s) | 1.68 (2H, m) | 1.61 (2H, m) |
| 3 | 3.93 (1H, dd, 8.8, 4.3) | 4.09 (1H, m) | 3.95 (1H, dd, 9.0, 4.6) | 3.91 (1H, dd, 8.8, 4.3) | 3.91 (1H, dd, 8.7, 4.6) |
| 5 | 4.80 (1H, s) | 4.23 (1H, s) | 4.80 (1H, s) | 4.80 (1H, s) | 3.42 (1H, s) |
| 7 | 3.28 (1H, d, 8.3) | 3.58 (1H, s) | 3.30 (1H, d, 8.5) | 3.28 (1H, br. s) | 3.12 (1H, d, 8.1) |
| 8 | 4.14 (1H, dd, 8.3, 6.3) | | 4.20 (1H, dd, 8.5, 5.2) | 4.14 (1H, dd, 7.1, 6.5) | 3.83 (1H, m) |
| 9 | 5.94 (1H, dd, 14.5, 6.3) | 5.98 (1H, d, 15.2) | 6.37 (1H, dd, 15.3, 5.2) | 5.92 (1H, dd, 14.3, 6.5) | 2.00 (1H, m); 1.89 (1H, m) |
| 10 | 6.45 (1H, dd, 14.5, 10.6) | 6.34 (1H, dd, 15.2, 10.9) | 6.63 (1H, dd, 15.3, 11.0) | 6.45 (1H, overlapped) | 4.53 (1H, br. s) |
| 11 | 6.50 (1H, dd, 14.5, 10.6) | 6.44 (1H, dd, 15.2, 10.9) | 7.12 (1H, dd, 15.3, 11.0) | 6.46 (1H, overlapped) | 6.06 (1H, dd, 15.2, 4.8) |
| 12 | 6.33 (1H, dd, 14.5, 11.3) | 6.29 (1H, dd, 14.5, 10.9) | 6.18 (1H, dd, 15.3, 8.0) | 6.35 (1H, overlapped) | 6.49 (1H, dd, 15.2, 11.2) |
| 13 | 7.16 (1H, dd, 15.2, 11.3) | 7.15 (1H, dd, 15.2, 10.9) | 9.58 (1H, d, 8.0) | 7.16 (1H, dd, 14.7, 11.3) | 7.16 (1H, dd, 15.2, 11.2) |
| 14 | 6.04 (1H, d, 15.2) | 6.04 (1H, d, 15.2) | | 6.33 (1H, d, 14.7) | 6.34 (1H, d, 15.2) |
| 16 | 5.84 (1H, d, 1.9) | 5.84 (1H, d, 1.9) | | | |
| 18 | 5.45 (1H, d, 1.9) | 5.46 (1H, d, 1.9) | | 5.48 (1H, s) | 5.50 (1H, s) |
| 20 | 1.19 (3H, s) | 1.33 (3H, s) | 1.21 (3H, s) | 1.17 (3H, s) | 1.24 (3H, s) |
| 21 | 1.27 (3H, s) | 1.36 (3H, s) | 1.27 (3H, s) | 1.25 (3H, s) | 1.35 (3H, s) |
| 22 | | | | 1.95 (3H, s) | 1.95 (3H, s) |
| Me-8 | | 1.26 (3H, s) | | | |
| 2' | 2.17 (3H, s) | | 2.17 (3H, s) | 2.43 (2H, m) | |
| 3' | | | | 1.19 (3H, t, 7.6) | |
| OMe-17 | 3.80 (3H, s) | 3.81 (3H, s) | | 3.82 (3H, s) | 3.82 (3H, s) |

[a]Measured at 600 MHz; [b]Measured at 500 MHz; [c]Measured at 400 MHz.

**Table 2.** $^1$**H NMR spectroscopic data of aurovertins O–S (6–10) in** $CDCl_3$

| no. | **6**[a] | **7**[c] | **8**[c] | **9**[b] | **10**[a] |
|---|---|---|---|---|---|
| 1 | 1.03 (3H, t, 7.5) | 1.03 (3H, t, 7.5) | 1.06 (3H, t, 7.5) | 1.06 (3H, t, 7.5) | 1.00 (3H, t, 7.6) |
| 2 | 1.65 (2H, m) | 1.61 (2H, m) | 1.65 (2H, m) | 1.66 (2H, m) | 1.52 (2H, m) |
| 3 | 3.89 (1H, m) | 3.84 (1H, m) | 3.88 (1H, dd, 9.0, 4.2) | 3.88 (1H, m) | 3.86 (1H, m) |
| 5 | 4.77 (1H, s) | 4.66 (1H, s) | 4.69 (1H, s) | 4.75 (1H, s) | 4.79 (1H, s) |
| 7 | 3.24 (1H, d, 8.3) | 3.54 (1H, m) | 3.53 (1H, d, 7.9) | 3.66 (1H, d, 7.6) | 3.73 (1H, d, 8.2) |
| 8 | 3.82 (1H, m) | 3.70 (1H, m) | 3.70 (1H, dd, 7.9, 4.3) | 3.80 (1H, m) | 3.56 (1H, dd, 8.2, 6.4) |
| 9 | 2.02 (1H, m); 1.93 (1H, m) | 4.33 (1H, m) | 4.34 (1H, overlapped) | 3.79 (1H, m) | 3.77 (1H, dd, 8.0, 6.4) |
| 10 | 4.53 (1H, br. s) | 5.87 (1H, overlapped) | 5.89 (1H, dd, 15.5, 6.1) | 4.47 (1H, br. s) | 3.89 (1H, m) |
| 11 | 6.06 (1H, dd, 15.2, 4.6) | 5.86 (1H, overlapped) | 5.69 (1H, dd, 15.5, 7.2) | 6.11 (1H, dd, 15.2, 4.6) | 5.84 (1H, dd, 15.2, 8.2) |
| 12 | 6.50 (1H, dd, 15.2, 11.3) | 4.84 (1H, m) | 4.32 (1H, overlapped) | 6.52 (1H, dd, 15.2, 11.2) | 6.47 (1H, dd, 15.2, 11.3) |
| 13 | 7.18 (1H, dd, 15.2, 11.3) | 6.65 (1H, dd, 15.2, 6.3) | 6.62 (1H, dd, 15.5, 6.4) | 7.14 (1H, dd, 15.2, 11.2) | 7.16 (1H, dd, 15.2, 11.3) |
| 14 | 6.36 (1H, d, 15.2) | 6.50 (1H, d, 15.2) | 6.50 (1H, d, 15.5) | 6.36 (1H, d, 15.2) | 6.39 (1H, d, 15.2) |
| 18 | 5.51 (1H, s) | 5.46 (1H, s) | 5.50 (1H, s) | 5.50 (1H, s) | 5.53 (1H, s) |
| 20 | 1.15 (3H, s) | 1.11 (3H, s) | 1.15 (3H, s) | 1.14 (3H, s) | 1.17 (3H, s) |
| 21 | 1.25 (3H, s) | 1.19 (3H, s) | 1.25 (3H, s) | 1.27 (3H, s) | 1.26 (3H, s) |
| 22 | 1.95 (3H, s) | 1.91 (3H, s) | 1.96 (1H, s) | 1.95 (3H, s) | 1.97 (3H, s) |
| 2' | 2.15 (3H, s) | 2.11 (3H, s) | 2.14 (3H, s) | 2.15 (3H, s) | 2.14 (3H, s) |
| OMe-10 | | | | | 3.34 (3H, s) |
| OMe-12 | | | 3.34 (3H, s) | | |
| OMe-17 | 3.83 (3H, s) | 3.79 (3H, s) | 3.82 (3H, s) | 3.83 (3H, s) | 3.84 (3H, s) |

[a]Measured at 600 MHz; [b]Measured at 500 MHz; [c]Measured at 400 MHz.

The HREIMS indicated the molecular formula of aurovertin M (**4**) to be $C_{26}H_{34}O_8$ by the ion peak at *m/z* 474.2264 $[M]^+$. The NMR data of **4** resembled those of **11**[5] except that the acetoxy group at C-5 was replaced by a propionyloxy group, which was supported by the $^1$H-$^1$H COSY correlation between $\delta_H$ 2.43 (2H, m, H-2') and 1.19 (3H, t, $J$ = 7.6 Hz, Me-3'), as well as the HMBC correlation from Me-3' to $\delta_C$ 27.5 (t, C-2') and 173.3 (s, C-1'). Accordingly, compound **4** was identified as aurovertin M.

Aurovertin N (**5**) was isolated as yellow syrup. The molecular formula $C_{23}H_{32}O_8$ was determined by HREIMS at *m/z* 436.2097 $[M]^+$. The 1D and 2D NMR data suggested that the structure of **5** was related to that of aurovertin E (**13**),[8] except that the double bond between C-9 and C-10 in **14** was opened to form a hydroxymethine at C-10 ($\delta_C$ 69.8), as implied by the HMBC correlations from $\delta_H$ 4.53 (1H, br. s, H-10) to $\delta_C$ 75.9 (d, C-8), 39.6 (t, C-9), and 141.5 (d, C-11). Detailed analysis of other spectroscopic data suggested that the other parts of **5** were the same to those of **13**. Thus, compound **5** was established as aurovertin N.

The same to **5**, all the spectral data suggested that compound **6** also showed similar patterns to those of aurovertin B (**11**),[5] except that the double bond between C-9 and C-10 was opened to form a hydroxymethine group at C-10 ($\delta_C$ 69.8), as implied by the HMBC correlations from $\delta_H$ 4.53 (1H, br. s, H-10) to $\delta_C$ 75.9 (d, C-8), 39.6 (t, C-9), and 141.5 (d, C-11). Detailed analysis of other spectroscopic data suggested that the other parts of **6** were the same to those of **11**. Thus, compound **6** was established as aurovertin O.

Aurovertin P (**7**) had the molecular formular $C_{25}H_{34}O_{10}$, as established by the HREIMS at *m/z* 494.2157 $[M]^+$. All the data suggested that the structure of **7** was closely related to that of **11**.[5] A significant change was that C-9 and C-12 in **7** were two oxygenated methines at $\delta_C$ 72.3 (C-9) and 71.8 (C-12) instead of two olefinic carbons in **11**, which was supported by the HMBC correlations from $\delta_H$ 4.33 (1H, m, H-9) to $\delta_C$ 79.3 (d, C-8) and 130.0 (d, C-10), and from $\delta_H$ 4.84 (1H, m, H-12) to $\delta_C$ 131.7 (d, C-11) and 138.6 (d, C-13), as well as the $^1$H-$^1$H COSY correlations from H-8 to H-13. Further analysis of other 2D NMR data suggested that the other parts were the same to those of **11**. Therefore, compound **7** was established as aurovertin P, as shown.

The NMR data of aurovertin Q (**8**) were closely related to those of **7**, except for the existence of a methoxy group at C-12 ($\delta_C$ 81.0) in **8** rather than the hydroxy group at C-12 in **7**, as established by the HMBC correlation of $\delta_H$ 3.34 (3H, s, OMe) to $\delta_C$ 81.0 (d, C-12). Thus, compound **8** was elucidated as aurovertin Q, as shown.

Aurovertin R (**9**) possessed a molecular formula $C_{25}H_{34}O_{10}$ according to the ion peak at *m/z* 494.2144 $[M]^+$ in the HREIMS. The NMR data suggested that the structure of **9** was related to that of **6**, except for one more hydroxy group placed at C-9, as indicated by the HMBC correlation from $\delta_H$ 3.79 (1H, m, H-9) to $\delta_C$ 77.0 (d, C-8) and 71.3 (d, C-10), as well as the $^1$H-$^1$H COSY correlation from H-8 to H-10. Thus, compound **9** was determined as aurovertin R, as shown.

The NMR data suggested that aurovertin S (**10**) had the related structure to that of **9** except for the methoxy group at C-10 in **10** rather than the hydroxy group in **9**, as indicated by the signal at $\delta_H$ 3.34 (3H, s, OMe), as well as the HMBC correlation from $\delta_H$ 3.34 (3H, s, OMe) to $\delta_C$ 83.1 (d, C-10). Detailed analysis of other 2D NMR data suggested that the other parts of **10** were the same to those of **9**. Compound **10** was, therefore, determined as aurovertin S, as shown.

The structures of the known compounds **11**–**14** isolated were identified as aurovertin B,[5] aurovertin C,[22] aurovertin E,[8] and aurovertin I,[10] respectively, by comparison of their spectroscopic data with literature values. All aurovertins

**Table 3. $^{13}$C NMR spectroscopic data of aurovertins J–S (1–10) in $CDCl_3$**

| no. | **1**[a] | **2**[b] | **3**[a] | **4**[c] | **5**[c] | **6**[a] | **7**[c] | **8**[c] | **9**[b] | **10**[a] |
|---|---|---|---|---|---|---|---|---|---|---|
| 1 | 11.8 q | | 11.7 q | 11.7 q | 11.9 q | 11.8 q | 11.7 q | 11.7 q | 11.8 q | 11.9 q |
| 2 | 20.1 t | 13.2 q | 20.1 t | 20.1 t | 20.1 t | 20.0 t | 20.1 t | 20.2 t | 20.1 t | 20.3 t |
| 3 | 85.5 d | 80.3 d | 85.5 d | 85.5 d | 84.6 d | 85.3 d | 85.2 d | 85.2 d | 85.4 d | 85.3 d |
| 4 | 82.7 s | 83.0 s | 82.9 s | 82.7 s | 83.9 s | 82.8 s | 82.5 s | 82.6 s | 82.8 s | 82.6 s |
| 5 | 80.4 d | 75.4 d | 80.2 d | 80.2 d | 80.3 d | 80.3 d | 80.4 d | 80.3 d | 80.5 d | 80.2 d |
| 6 | 83.4 s | 82.6 s | 83.4 s | 83.4 s | 84.1 s | 83.3 s | 83.5 s | 83.3 s | 83.3 s | 83.5 s |
| 7 | 76.3 d | 79.6 d | 76.2 d | 76.2 d | 76.2 d | 76.3 d | 71.5 d | 72.6 d | 73.5 d | 71.9 d |
| 8 | 77.9 d | 78.2 s | 77.2 d | 77.8 d | 75.9 d | 75.9 d | 79.3 d | 79.6 d | 77.0 d | 76.2 d |
| 9 | 134.5 d | 147.1 d | 142.1 d | 134.2 d | 39.6 t | 39.6 t | 72.3 d | 72.8 d | 75.6 d | 71.9 d |
| 10 | 131.5 d | 128.0 d | 128.4 d | 131.6 d | 69.8 d | 69.8 d | 130.0 d | 131.9 d | 71.3 d | 83.1 d |
| 11 | 137.6 d | 137.7 d | 151.3 d | 137.0 d | 141.5 d | 141.5 d | 131.7 d | 130.3 d | 138.3 d | 135.2 d |
| 12 | 131.5 d | 130.9 d | 132.1 d | 132.0 d | 128.5 d | 128.7 d | 71.8 d | 81.0 d | 130.5 d | 133.9 d |
| 13 | 135.9 d | 135.8 d | 194.0 d | 135.6 d | 135.3 d | 135.1 d | 138.6 d | 136.8 d | 135.1 d | 134.3 d |
| 14 | 122.0 d | 121.9 d | | 119.4 d | 119.1 d | 119.3 d | 117.4 d | 118.6 d | 119.8 d | 120.3 d |
| 15 | 158.6 s | 158.7 s | | 154.2 s | 154.3 s | 154.2 s | 153.7 s | 153.4 s | 154.2 s | 152.8 s |
| 16 | 101.0 d | 101.0 d | | 108.0 s | 108.0 s | 108.0 s | 108.2 s | 108.3 s | 108.2 s | 108.6 s |
| 17 | 171.0 s | 171.1 s | | 170.6 s | 170.8 s | 170.7 s | 170.9 s | 170.7 s | 170.7 s | 170.5 s |
| 18 | 88.7 d | 88.7 d | | 88.7 d | 88.8 d | 88.8 d | 88.7 d | 89.1 d | 88.9 d | 89.2 d |
| 19 | 164.1 s | 164.0 s | | 163.7 s | 163.9 s | 163.8 s | 164.1 s | 163.7 s | 163.7 s | 163.6 s |
| 20 | 16.4 q | 17.3 q | 16.3 q | 16.4 q | 16.5 q | 16.5 q | 16.4 q | 16.4 q | 16.4 q | 16.5 q |
| 21 | 15.0 q | 18.6 q | 15.0 q | 15.0 q | 14.6 q | 15.0 q | 15.1 q | 15.0 q | 14.9 q | 15.1 q |
| 22 | | | | 8.8, q | 8.8, q | 8.8 q | 8.7 q | 8.9 q | 8.8 q | 8.9 q |
| Me-8 | | 26.5 q | | | | | | | | |
| 1' | 169.9 s | | 169.9 s | 173.3 s | | 169.9 s | 170.0 s | 170.0 s | 169.8 s | 169.7 s |
| 2' | 20.8 q | | 20.8 q | 27.5 t | | 20.8 q | 20.7 q | 20.8 q | 20.7 q | 20.7 q |
| 3' | | | | 9.2 q | | | | | | |
| OMe-10 | | | | | | | | | | 57.0 q |
| OMe-12 | | | | | | | | 56.2 q | | |
| OMe-17 | 56.9 q | 55.9 q | | 56.1 q | 56.2 q | 56.2 q | 56.2 q | 56.3 q | 56.2 q | 56.2 q |

[a]Measured at 150 MHz; [b]Measured at 125 MHz; [c]Measured at 100 MHz.

obtained from cultures of the basidiomycete *A. confluens* were yellow syrup, and could not obtain as crystals in diverse solution systems. In order to elucidate the absolute configuration of the side chain in compounds **5–10**, modified Mosher reaction[24] of compound **5** and acylation of compound **7** with 4-bromobenzoyl chloride[25] for crystals were all in failure. Thus, the stereoconfigurations of compounds **5–10** were not determined wholly.

All compounds were evaluated for their cytotoxicities against five human cancer cell lines. The results showed that compounds **7** and **11** exhibited moderate cytotoxicities compared with those of cisplatin (Table 4), while the other compounds were inactive ($IC_{50} > 40$ $\mu$M).

## Experimental Section

**General Experimental Procedures.** Optical rotations were measured on a Jasco-P-1020 polarimeter. UV spectra were measured on a Shimadzu UV-2401 PC spectrophotometer. IR spectra were obtained by using a Bruker Tensor 27 FT-IR spectrometer with KBr pellets. NMR spectra were acquired with instruments of Avance III 600 or Bruker DRX-500 or Bruker AV 400. ESIMS and HREIMS were measured on Bruker HCT/Esquire and VG Autospec-3000 mass spectrometer respectively. Preparative HPLC was performed on an Agilent 1100 series with a Zorbax SB-C18 (5 $\mu$m, 9.4 × 150 mm) column. Preparative MPLC was performed on a Büchi apparatus equipped with Büchi fraction collector C-660, Büchi pump module C-605 and manager C-615. Silica gel (200–300 mesh and 80–100 mesh, Qingdao Marine Chemical Inc., China), RP-18 gel (40–75 $\mu$m, Fuji Silysia Chemical Ltd., Japan) and Sephadex LH-20 (Amersham Biosciences, Sweden) were used for column chromatography. Fractions were monitored by TLC (Qingdao Marine Chemical Inc., China) and spots visualized by heating silica gel plates immersed in vanillin-$H_2SO_4$ in EtOH.

**Table 4. Cytotoxicity of compounds 7 and 11 ($IC_{50}$, $\mu$M)**

| Entry | HL-60 | SMMC-7721 | A-549 | MCF-7 | SW480 |
|---|---|---|---|---|---|
| **7** | > 40 | 18.3 | > 40 | > 40 | 14.4 |
| **11** | 14.7 | 10.8 | 14.7 | 18.8 | 22.4 |
| cisplatin | 1.8 | 16.5 | 10.2 | 15.8 | 26.5 |

**Fungal Material and Cultivation Conditions.** The fungus *A. confluens* was collected from Ailao Mountain of Yunnan Province, China, in July 2003, and identified by Prof. Mu Zang, Kunming Institute of Botany. The voucher specimen

(HFG0307252) was deposited at the Herbarium of the Kunming Institute of Botany, CAS. Culture medium: glucose (5%), pork peptone (0.15%), yeast (0.5%), $KH_2PO_4$ (0.05%), $MgSO_4$ (0.05%), The initial pH was adjusted to 6.0, the fermentation was first carried out on an erlenmeyer flask for six days till the mycelium biomass reached to the maximum. Later it was transferred to a fermentation tank (100 L) at 24 °C and 250 rpm for twenty days, ventilation was settled to 1.0 vvm (vvm: air volume/culture volumn/min).

**Extraction and Isolation.** The culture broth (70 L) was extracted three times with EtOAc (3 × 10 L). The combined EtOAc extracts were evaporated in vacuo to give a residue (50.0 g). The residue was subjected to silica gel column chromatography (CC) with a gradient elution system of chloroform-methanol (100:0 → 0:100) to obtain ten fractions (A–K). Fraction D was subjected to preparative MPLC with a reversed-phased $C_{18}$ column ($MeOH$-$H_2O$, 0–60%) to obtain subfractions D01–D10. Fraction D05 was eluted with petroleum ether (PE)-EtOAc (4:1) and then subjected to Sephadex LH-20 CC ($CHCl_3$-MeOH, 1:1) to give **5** (8.0 mg) and **9** (5.0 mg). Fraction D10 eluted with PE-acetone (5:1) was further separated by preparative HPLC ($CH_3CN$-$H_2O$, 30%) to give **2** (6.5 mg) and **4** (15.0 mg). Fraction G was chromatographed over a silica gel column using PE-acetone (10:1 → 0:1) to produce fractions G01–G06. **8** (6.0 mg) and **13** (20.0 mg) were afforded from fraction G04 by preparative HPLC ($CH_3CN$-$H_2O$, 20%), compound **1** (3.0 mg) was also obtained by preparative HPLC ($CH_3CN$-$H_2O$, 30%) from the G03 fraction. Fraction G05 was subjected to a RP-18 column (MeOH-$H_2O$, 40%), then purified on a silica gel column (PE-acetone, 2:1) to afford **3** (2.0 mg) and **11** (10.0 mg). Fraction G06 was separated by repeated silica gel column chromatography (PE-acetone, 6:1 → 0:1) to yield fractions G061-G068. Fraction G061 was chromatographed on a RP-18 column (MeOH-$H_2O$, 50%) and then purified on a silica gel column (PE-EtOAc, 2:1) to yield **6** (2.0 mg) and **10** (2.0 mg). Compound **12** (12.0 mg) was obtained from fraction G063 after preparative HPLC ($CH_3CN$-$H_2O$, 10%), followed by Sephadex LH-20 CC ($CHCl_3$-MeOH, 1:1). Fraction I was eluted with PE-acetone (4:1). It was further chromatographed on a RP-18 column (MeOH-$H_2O$, 50%) and then purified by CC on Sephadex LH-20 ($CHCl_3$-MeOH, 1:1) to give **7** (12.0 mg) and **14** (20.0 mg).

**Aurovertin J (1):** yellow syrup; $[\alpha]^{15}_{D}$ – 15.6 (*c* 0.23, MeOH); UV (MeOH) $\lambda_{max}$ (log ε) 346 (2.9), 263 (2.9), 247 (2.9), 206 (3.0) nm; IR (KBr) $\nu_{max}$ 3441, 1632, 1108 $cm^{-1}$; $^1H$ NMR ($CDCl_3$, 600 MHz) and $^{13}C$ NMR ($CDCl_3$, 150 MHz) data, see Tables 1 and 3; ESIMS (positive) *m/z* 469 $[M + Na]^+$; HREIMS *m/z* 446.1934 (calcd for $C_{24}H_{30}O_8$, 446.1941).

**Aurovertin K (2):** yellow syrup; $[\alpha]^{15}_{D}$ – 2.0 (*c* 0.33, MeOH); UV (MeOH) $\lambda_{max}$ (log ε) 330 (3.3), 272 (3.3), 205 (3.4) nm; IR (KBr) $\nu_{max}$ 3442, 1630 $cm^{-1}$; $^1H$ NMR ($CDCl_3$, 500 MHz) and $^{13}C$ NMR ($CDCl_3$, 125 MHz) data, see Tables 1 and 3; ESIMS (positive) *m/z* 427 $[M + Na]^+$; HREIMS *m/z* 404.1832 (calcd for $C_{22}H_{28}O_7$, 404.1835).

**Aurovertin L (3):** yellow syrup; $[\alpha]^{15}_{D}$ – 17.1 (*c* 0.20, MeOH); UV (MeOH) $\lambda_{max}$ (log ε) 388 (1.3), 264 (2.3), 202 (2.5) nm; IR (KBr) $\nu_{max}$ 3440, 1632 $cm^{-1}$; $^1H$ NMR ($CDCl_3$, 600 MHz) and $^{13}C$ NMR ($CDCl_3$, 150 MHz) data, see Tables 1 and 3; ESIMS (positive) *m/z* 347 $[M + Na]^+$; HREIMS *m/z* 324.1564 (calcd for $C_{17}H_{24}O_6$, 324.1573).

**Aurovertin M (4):** yellow syrup; $[\alpha]^{15}_{D}$ – 38.5 (*c* 0.40, MeOH); UV (MeOH) $\lambda_{max}$ (log ε) 359 (3.5), 271 (3.6), 227 (3.4), 199 (3.4) nm; IR (KBr) $\nu_{max}$ 3441, 1630, 1110 $cm^{-1}$; $^1H$ NMR ($CDCl_3$, 400 MHz) and $^{13}C$ NMR ($CDCl_3$, 100 MHz) data, see Tables 1 and 3; ESIMS (positive) *m/z* 497 $[M + Na]^+$; HREIMS *m/z* 474.2264 (calcd for $C_{26}H_{34}O_8$, 474.2254).

**Aurovertin N (5):** yellow syrup; $[\alpha]^{15}_{D}$ – 10.6 (*c* 0.31, MeOH); UV (MeOH) $\lambda_{max}$ (log ε) 337 (3.2), 246 (3.4), 229 (3.4), 207 (3.3) nm; IR (KBr) $\nu_{max}$ 3442, 1632 $cm^{-1}$; $^1H$ NMR ($CDCl_3$, 400 MHz) and $^{13}C$ NMR ($CDCl_3$, 100 MHz) data, see Tables 1 and 3; ESIMS (positive) *m/z* 459 $[M + Na]^+$; HREIMS *m/z* 436.2097 (calcd for $C_{23}H_{32}O_8$, 436.2097).

**Aurovertin O (6):** yellow syrup; $[\alpha]^{15}_{D}$ – 13.9 (*c* 0.30, MeOH); UV (MeOH) $\lambda_{max}$ (log ε) 320 (2.9), 226 (3.2) nm; IR (KBr) $\nu_{max}$ 3442, 1631 $cm^{-1}$; $^1H$ NMR ($CDCl_3$, 600 MHz) and $^{13}C$ NMR ($CDCl_3$, 150 MHz) data, see Tables 2 and 3; ESIMS (positive) *m/z* 501 $[M + Na]^+$; HREIMS *m/z* 478.2190 (calcd for $C_{25}H_{34}O_9$, 478.2203).

**Aurovertin P (7):** yellow syrup; $[\alpha]^{15}_{D}$ – 10.3 (*c* 0.20, MeOH); UV (MeOH) $\lambda_{max}$ (log ε) 319 (3.2), 225 (3.7) nm; IR (KBr) $\nu_{max}$ 3443, 1638, 1251, 1037 $cm^{-1}$; $^1H$ NMR ($CDCl_3$, 400 MHz) and $^{13}C$ NMR ($CDCl_3$, 100 MHz) data, see Tables 2 and 3; ESIMS (positive) *m/z* 517 $[M + Na]^+$; HREIMS *m/z* 494.2157 (calcd for $C_{25}H_{34}O_{10}$, 494.2152).

**Aurovertin Q (8):** yellow syrup; $[\alpha]^{15}_{D}$ – 13.4 (*c* 0.30, MeOH); UV (MeOH) $\lambda_{max}$ (log ε) 333 (3.2), 245 (3.4), 227 (3.5), 197 (3.3) nm; IR (KBr) $\nu_{max}$ 3440, 1631 $cm^{-1}$; $^1H$ NMR ($CDCl_3$, 400 MHz) and $^{13}C$ NMR ($CDCl_3$, 100 MHz) data, see Tables 2 and 3; ESIMS (positive) *m/z* 531 $[M + Na]^+$; HREIMS *m/z* 508.2305 (calcd for $C_{26}H_{36}O_{10}$, 508.2308).

**Aurovertin R (9):** yellow syrup; $[\alpha]^{15}_{D}$ – 4.2 (*c* 0.30, MeOH); UV (MeOH) $\lambda_{max}$ (log ε) 340 (3.3), 247 (3.5), 212 (3.5) nm; IR (KBr) $\nu_{max}$ 3449, 1639, 1038 $cm^{-1}$; $^1H$ NMR ($CDCl_3$, 500 MHz) and $^{13}C$ NMR ($CDCl_3$, 125 MHz) data, see Tables 2 and 3; ESIMS (positive) *m/z* 517 $[M + Na]^+$; HREIMS *m/z* 494.2144 (calcd for $C_{25}H_{34}O_{10}$, 494.2152).

**Aurovertin S (10):** yellow syrup; $[\alpha]^{15}_{D}$ – 70.0 (*c* 0.20, MeOH); UV (MeOH) $\lambda_{max}$ (log ε) 338 (3.2), 248 (3.4), 199 (3.2) nm; IR (KBr) $\nu_{max}$ 3443, 1632 $cm^{-1}$; $^1H$ NMR ($CDCl_3$, 600 MHz) and $^{13}C$ NMR ($CDCl_3$, 150 MHz) data, see Tables 2 and 3; ESIMS (positive) *m/z* 531 $[M + Na]^+$; HREIMS *m/z* 508.2326 (calcd for $C_{26}H_{36}O_{10}$, 508.2308).

**Cytotoxic Assay.** All compounds were evaluated for their cytotoxicity against five human cancer cell lines, breast cancer SK-BR-3, hepatocellular carcinoma SMMC-7721, human myeloid leukemia HL-60, pancreatic cancer PANC-1, and lung cancer A-549 cells. Cells were cultured in RPMI-1640 or in DMEM medium (Hyclone, USA), supplemented with 10% fetal bovine serum (Hyclone, USA) in 5% $CO_2$ at 37 °C. The cytotoxicity assay was performed according to 3-(4,5-dimethylthiazol-2-yl)-2,5-diphenyl tetrazolium bromide (MTT) method in 96-well microplates.[26] Briefly, 100 $\mu$L of adherent cells were seeded into each well of 96-well cell culture plates and allowed to adhere for 12 h before addition of test compounds, while suspended cells were seeded just before drug addition with initial density of 1 × $10^5$ cells/mL. Each tumor cell line was exposed to the test compound at concentrations of 0.0625, 0.32, 1.6, 8, and 40 $\mu$M in triplicates for 48 h, and all tests were done in twice with cisplatin (Sigma, USA) as a positive control. After compound treatment, cell viability was detected and a cell growth curve was graphed. $IC_{50}$ values were calculated by Reed and Muench's method.[27]

## Electronic Supplementary Material

Supplementary material is available in the online version of this article at http://dx.doi.org/ 10.1007/s13659-012-0088-y and is accessible for authorized users.

## Acknowledgments

This work was financially supported by National Basic Research Program of China (973 Program, 2009CB522300), the National Natural Science Foundation of China (U1132607).

**Open Access** This article is distributed under the terms of the Creative Commons Attribution License which permits any use, distribution, and reproduction in any medium, provided the original author(s) and source are credited.

## Electronic supplementary material

Supplementary material, approximately 10.1 MB.

## References

[1] Baldwin C. L., Weaver L. C., Brooker R. M., Jacobsen T. N., Osborne C. E., Nash H. A. (1964). Lloydia.

[2] Lardy H. A., Connelly J. L., Johnson D. (1964). Biochemistry.

[3] Roberton A. M., Beechey R. B., Holloway C. T., Knight I. G. (1967). Biochem. J..

[4] Ebel R. E., Lardy H. A. (1975). J. Biol. Chem..

[5] Steyn P. S., Vleggaar R., Wessels P. L. (1981). J. Chem. Soc., Perkin Trans..

[6] Van Raaij M. J., Abrahams J. P., Leslie A. G. W., Walker J. E. (1996). Proc. Natl. Acad. Sci..

[7] Huang T. C., Chang H. Y., Hsu C. H., Kuo W. H., Chang K. J., Juan H. F. (2008). J. Proteome Res..

[8] Wang F., Luo D. Q., Liu J. K. (2005). J. Antibiot..

[9] Azumi M., Ishidoh K. I., Kinoshita H., Nihira T., Ihara F., Fujita T., Igarashi Y. (2008). J. Nat. Prod..

[10] Niu X. M., Wang Y. L., Chu Y. S., Xue H. X., Li N., Wei L. X., Mo M. H., Zhang K. Q. (2010). J. Agric. Food Chem..

[11] Nishiyama S., Toshima H., Kanai H., Yamamura S. (1986). Tetrahedron Lett..

[12] Nishiyama S., Toshima H., Kanai H., Yamamura S. (1988). Tetrahedron.

[13] Steyn, P. S.; Vleggaar, R.; Wessels, P. L. *J. Chem. Soc., Chem. Commun.* **1979**, 1041–1042.

[14] Ding Z. H., Dong Z. J., Liu J. K. (2001). Helv. Chim. Acta.

[15] Yang W. M., Liu J. K., Chen Q., Liu Y. D., Ding Z. H., Shen Z. Q., Chen Z. H. (2003). Planta Med..

[16] Hellwig V., Nopper R., Mauler F., Freitag J., Liu J. K., Ding Z. H., Stadler M. (2003). Arch. Pharm..

[17] Chen Q., Liu M. H., Yangl W. M., Zhang Y. L., Wang L., Liu J. K. (2004). Planta Med..

[18] Ye M., Liu J. K., Lu Z. X., Zhao Y., Liu S. F., Li L. L., Tan M., Weng X. X., Li W., Cao Y. (2005). FEBS Lett..

[19] Ye M., Luo X. J., Li L. L., Shi Y., Tan M., Weng X. X., Li W., Liu J. K., Cao Y. (2007). Cancer Lett..

[20] Liu Q. Y., Shu X. L., Sun A., Sun Q. L., Zhang C. X., An H. Z., Liu J. K., Cao X. T. (2008). Int. Immunopharmacol..

[21] Liu Q. Y., Shu X. L., Wang L., Sun A., Liu J. K., Cao X. T. (2008). Cell. Mol. Immunol..

[22] Zhou Z. Y., Liu R., Jiang M. Y., Zhang L., Niu Y., Zhu Y. C., Dong Z. J., Liu J. K. (2009). Chem. Pharm. Bull..

[23] Luo X. J., Li W., Yang L. F., Yu X. F., Xiao L. B., Tang M., Dong X., Deng Q. P., Bode A. M., Liu J. K., Cao Y. (2011). Eur. J. Pharmacol..

[24] Zhang H. W., Zhang J., Hu S., Zhang Z. J., Zhu C. J., Ng S. W., Tan R. X. (2010). Planta Med..

[25] Jiang R. W., Ye W. C., Shaw P. C., But P. P. H., Mak T. C. W. (2010). J. Mol. Struct..

[26] Mosmann T. (1983). J. Immunol. Methods.

[27] Reed L. J., Muench H. (1938). Am. J. Hygiene.

