# Supplementary material for: Ten new aurovertins from cultures of the basidiomycete Albatrellus confluens
Source: Nat Prod Bioprospect. 2013 Jan 12;3(1):8–13. doi: 10.1007/s13659-012-0088-y (PMC4131612; doi:10.1007/s13659-012-0088-y)
Supplement: Supplementary file 1 — Supplementary material, approximately 10.1 MB. [file 13659_2012_88_MOESM1_ESM.pdf]

Electronic Supplementary Material

Ten new aurovertins from cultures of the basidiomycete *Albatrellus confluens*

Hua GUO,[a,b,c] Tao FENG,[a] Zheng-Hui LI,[a] and Ji-Kai LIU[a,*]

[a]State Key Laboratory of Phytochemistry and Plant Resources in West China, Kunming Institute of Botany, Chinese Academy of Sciences, Kunming 650201, China

[b]School of Chemistry and Life Science, Anshan Normal College, Anshan 114005, China

[c]University of Chinese Academy of Sciences, Beijing 100049, China

Received 31 October 2012; Accepted 25 December 2012

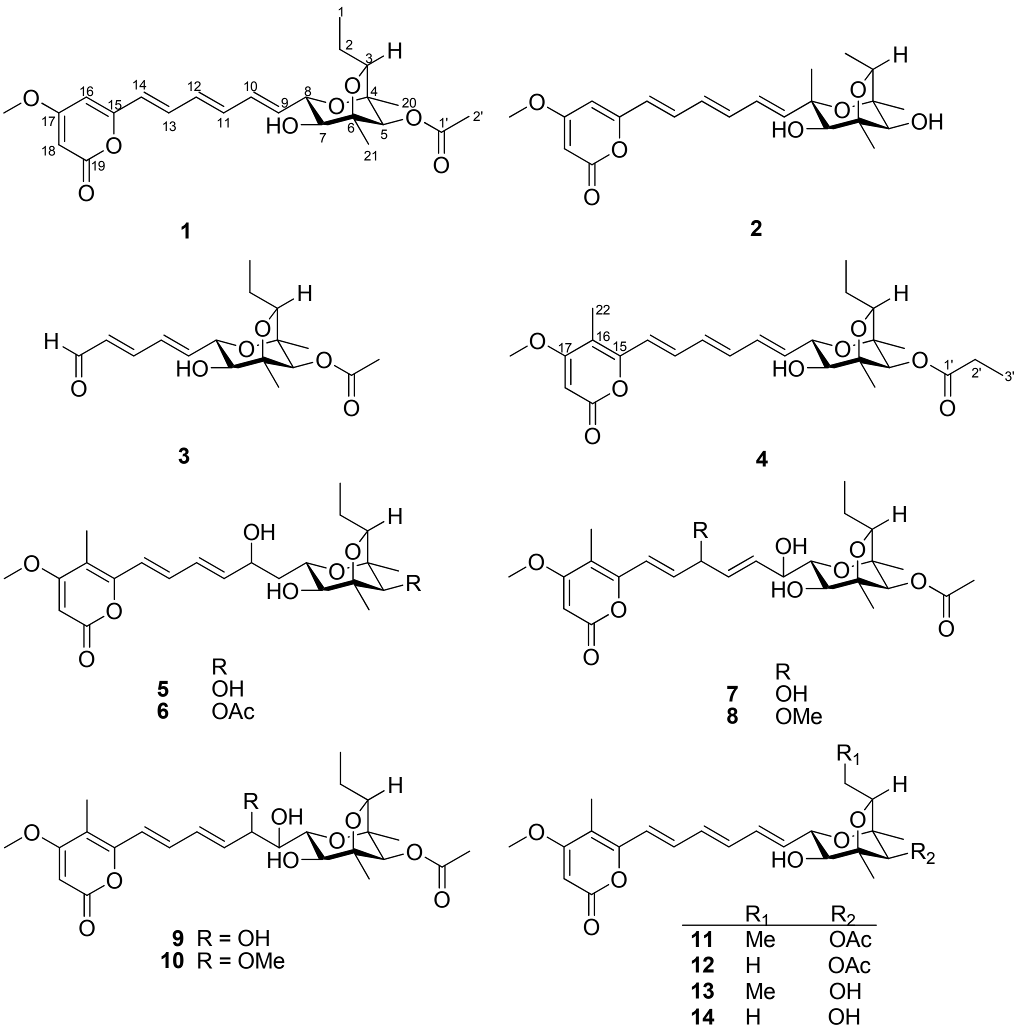

Structures of compounds **1**–**14**

*To whom correspondence should be addressed. E-mail: jkliu@mail.kib.ac.cn

Figure 1S–7S. NMR and MS of aurovertin J (**1**).

Figure 8S–14S. NMR and MS of aurovertin K (**2**).

Figure 15S–21S. NMR and MS of aurovertin L (**3**).

Figure 22S–28S. NMR and MS of aurovertin M (**4**).

Figure 29S–35S. NMR and MS of aurovertin N (**5**).

Figure 36S–42S. NMR and MS of aurovertin O (**6**).

Figure 43S–49S. NMR and MS of aurovertin P (**7**).

Figure 50S–56S. NMR and MS of aurovertin Q (**8**).

Figure 57S–63S. NMR and MS of aurovertin R (**9**).

Figure 64S–70S. NMR and MS of aurovertin S (**10**).

Figure 1S. ^{1}H NMR of aurovertin J (**1**).

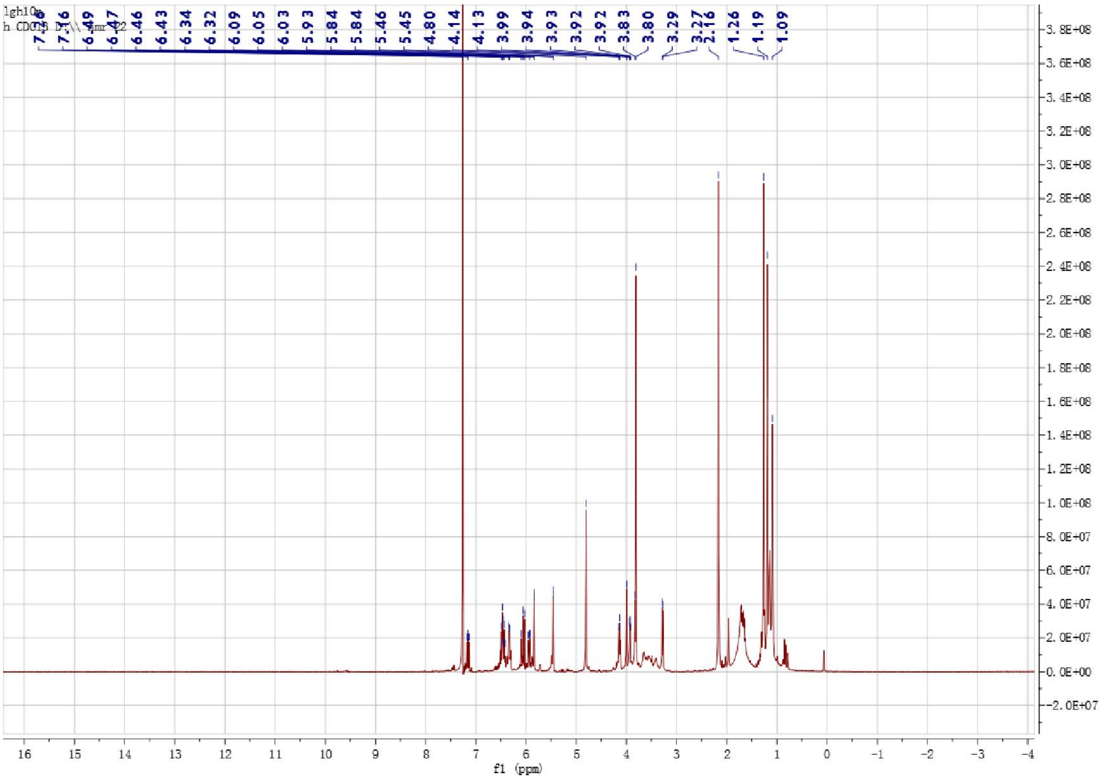

Figure 2S. ^{13}C NMR of aurovertin J (**1**).

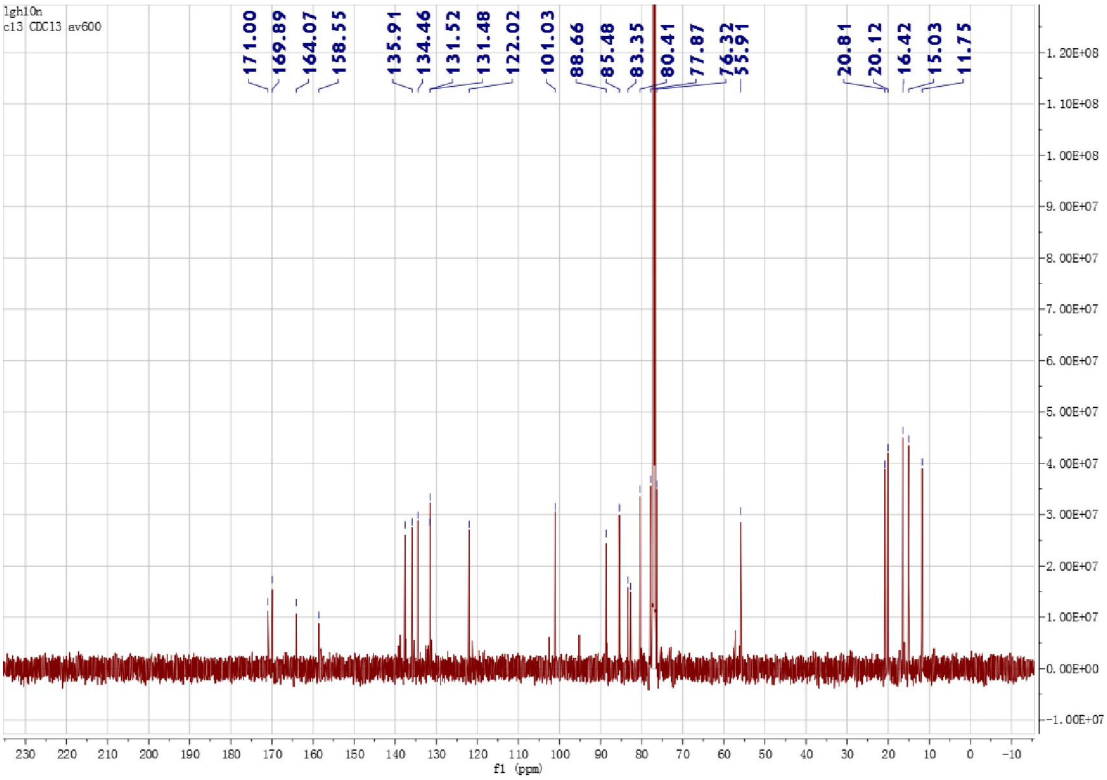

Figure 3S. HSQC of aurovertin J (**1**).

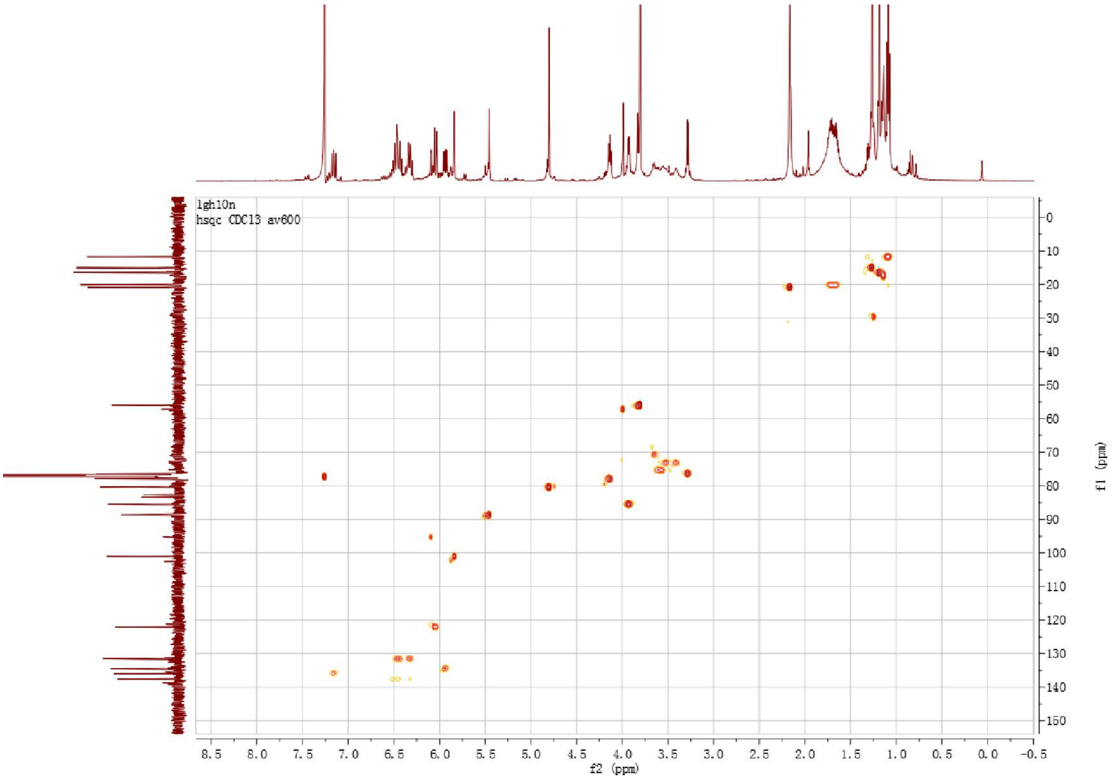

Figure 4S. HMBC aurovertin J (**1**)

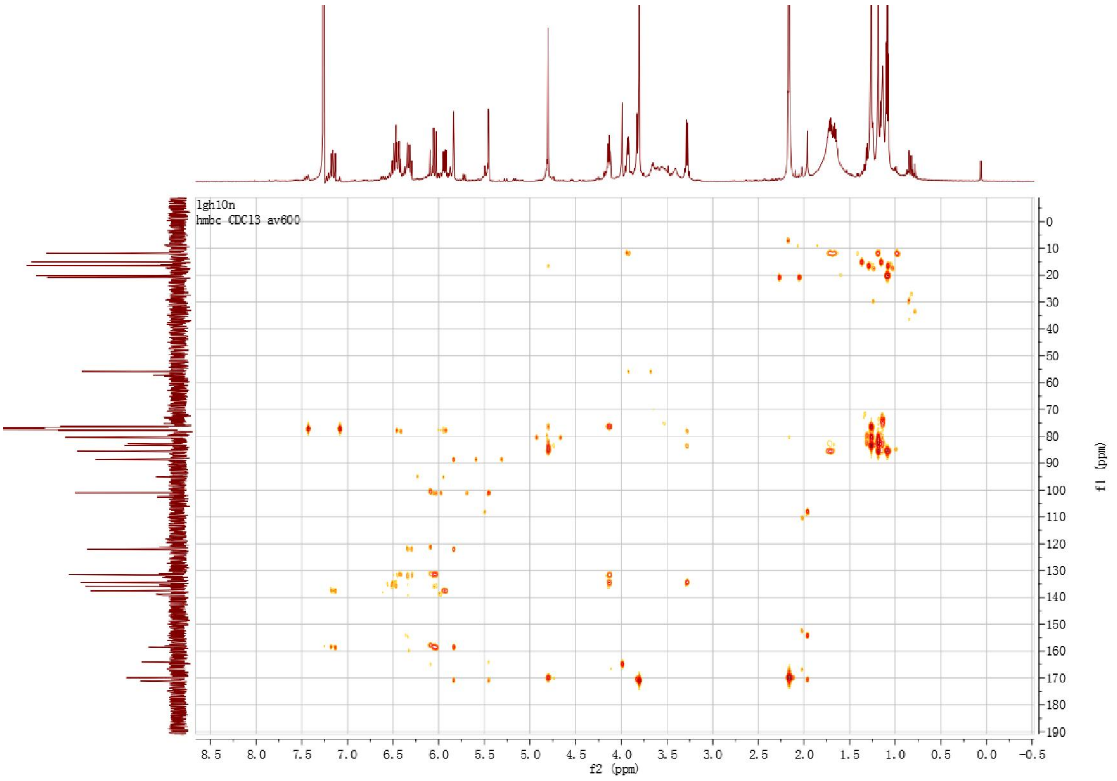

Figure 5S. ^{1}H-^{1}H COSY of aurovertin J (**1**).

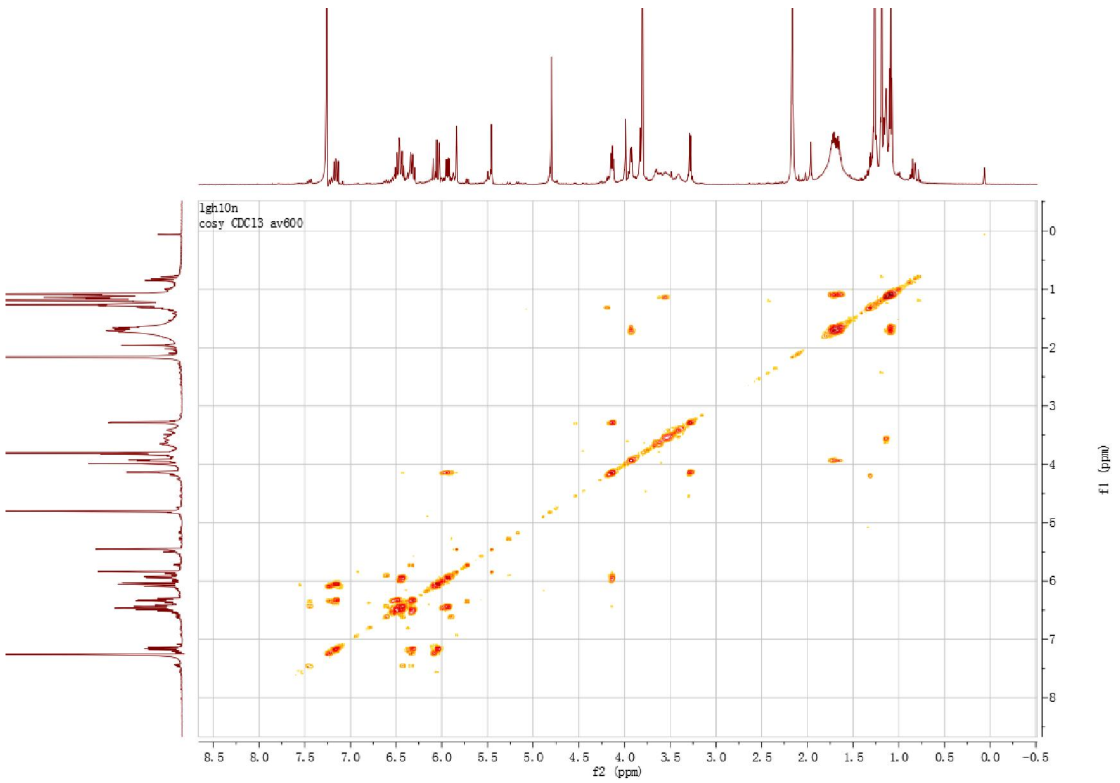

Figure 6S. ROESY of aurovertin J (**1**).

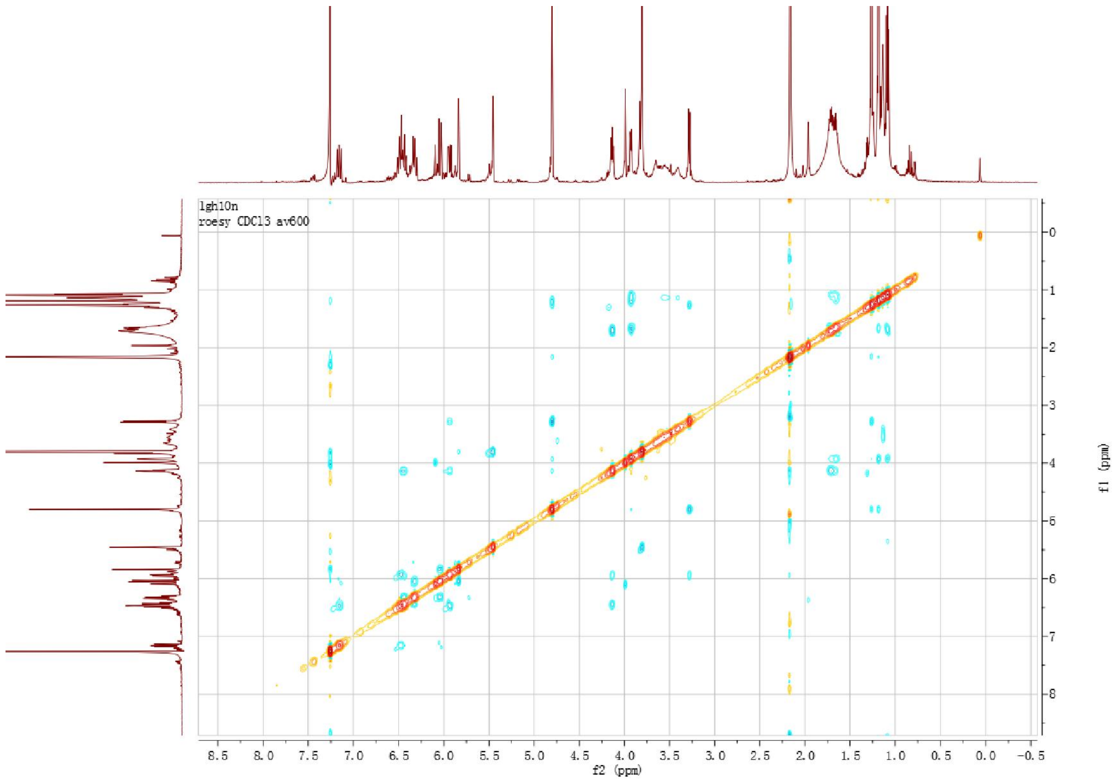

Figure 7S. HREIMS of aurovertin J (**1**).

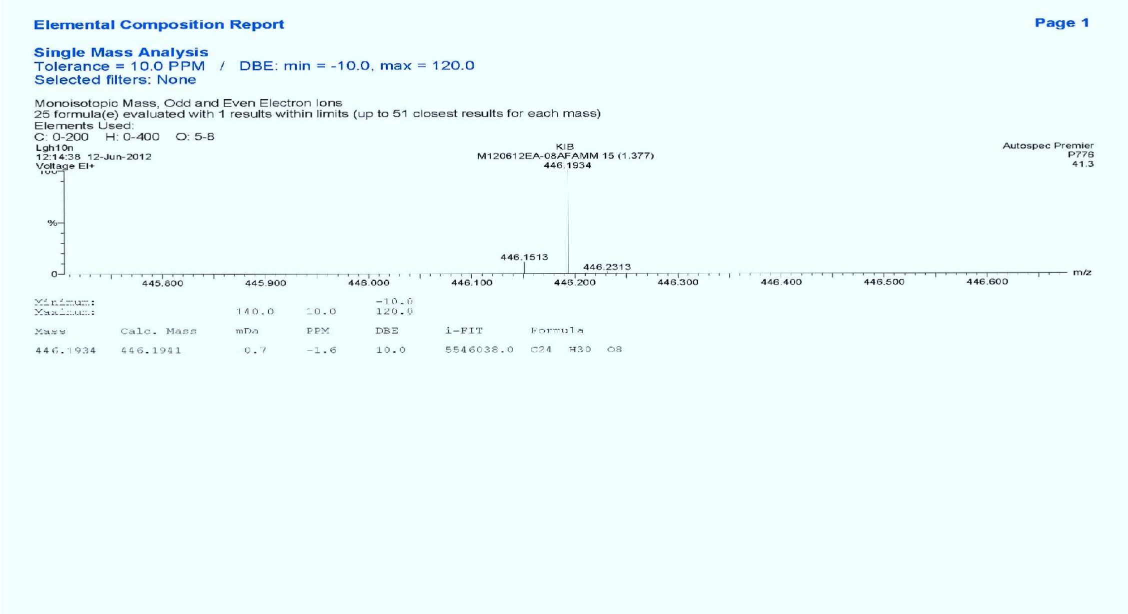

Figure 8S. ^{1}H NMR of aurovertin K (**2**).

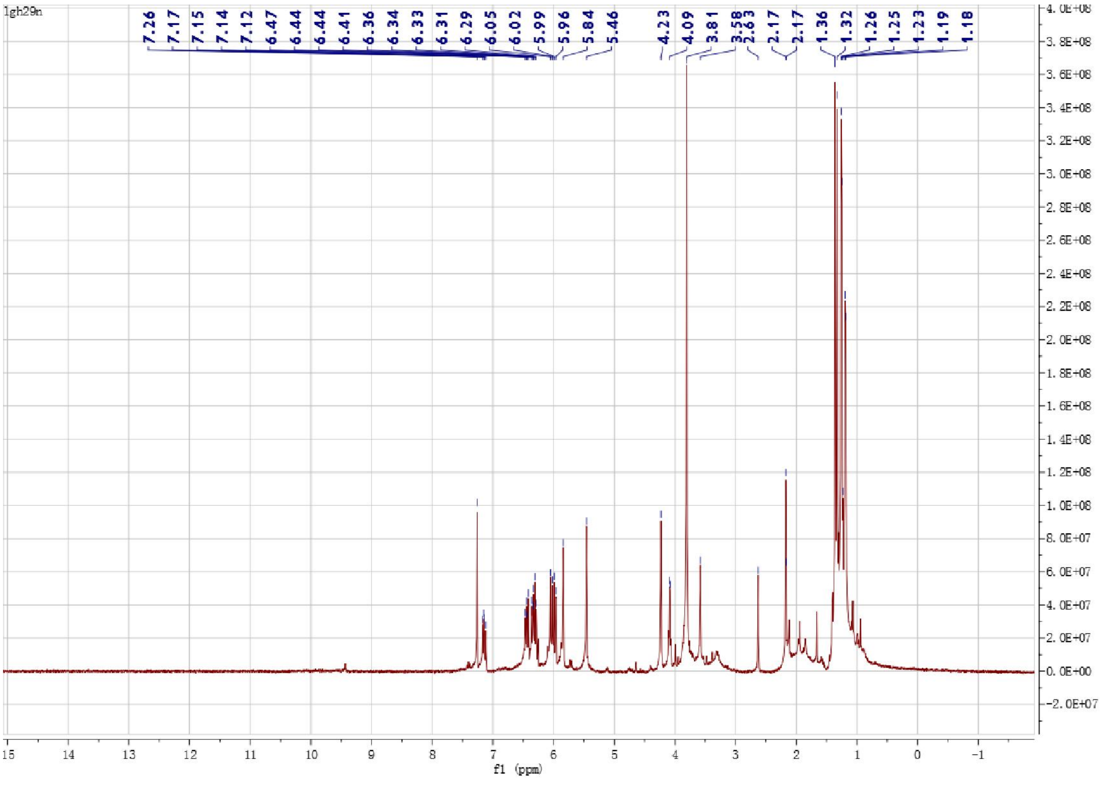

Figure 9S. ^{13}C NMR of aurovertin K (**2**).

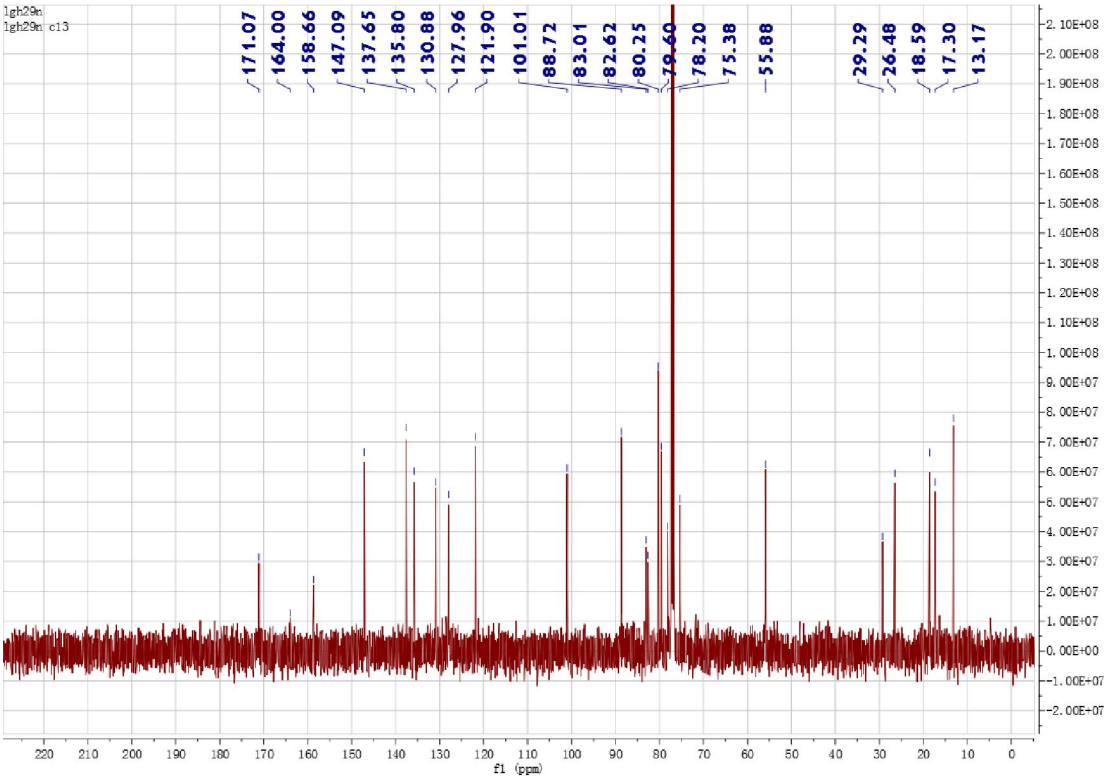

Figure 10S. HSQC of aurovertin K (**2**).

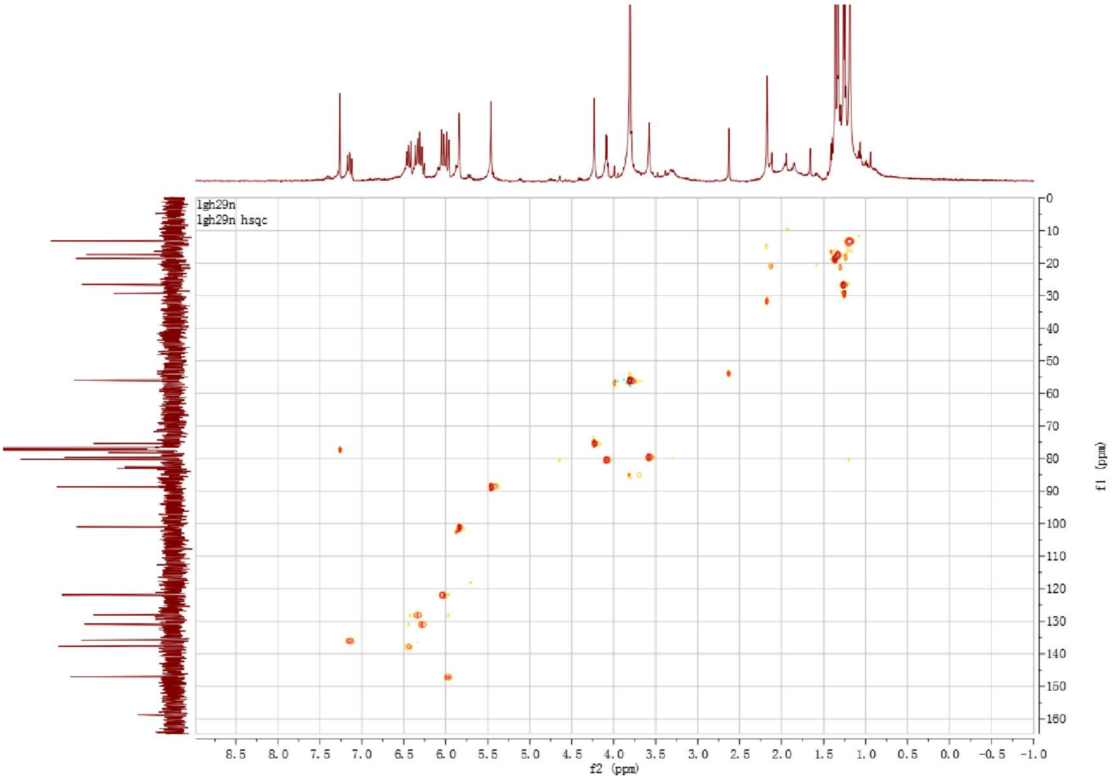

Figure 11S. HMBC aurovertin K (**2**)

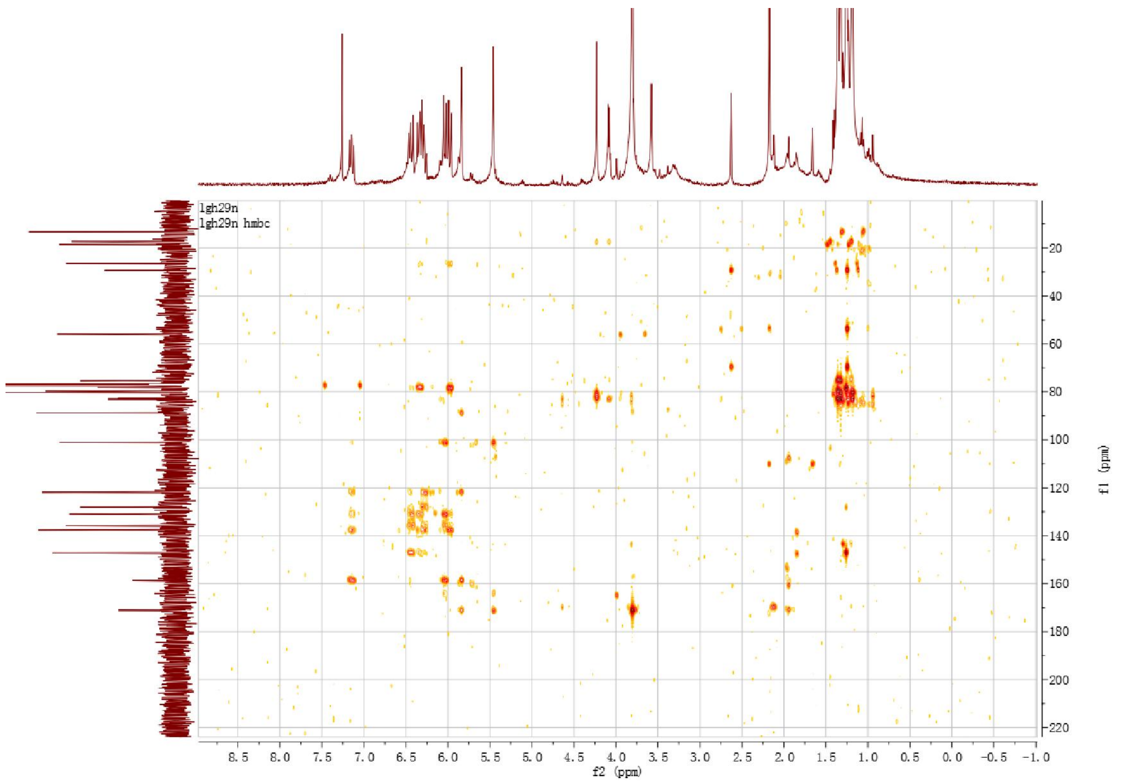

Figure 12S. ^{1}H-^{1}H COSY of aurovertin K (**2**).

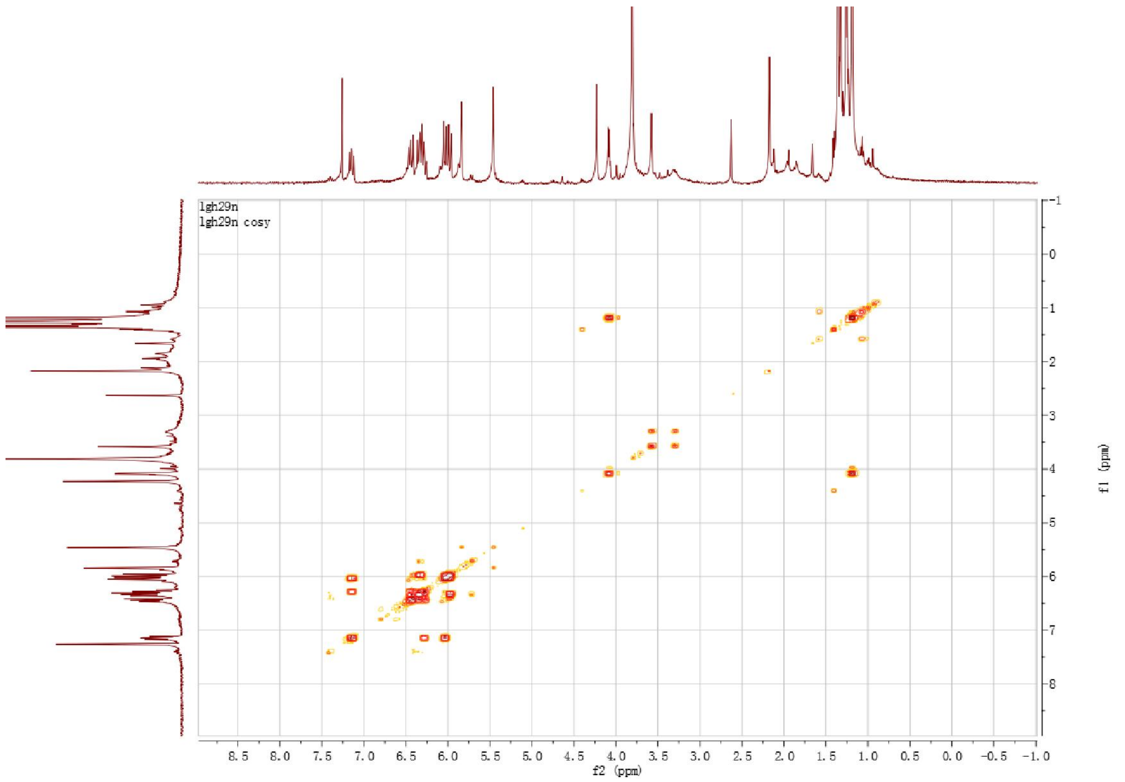

Figure 13S. ROESY of aurovertin K (**2**).

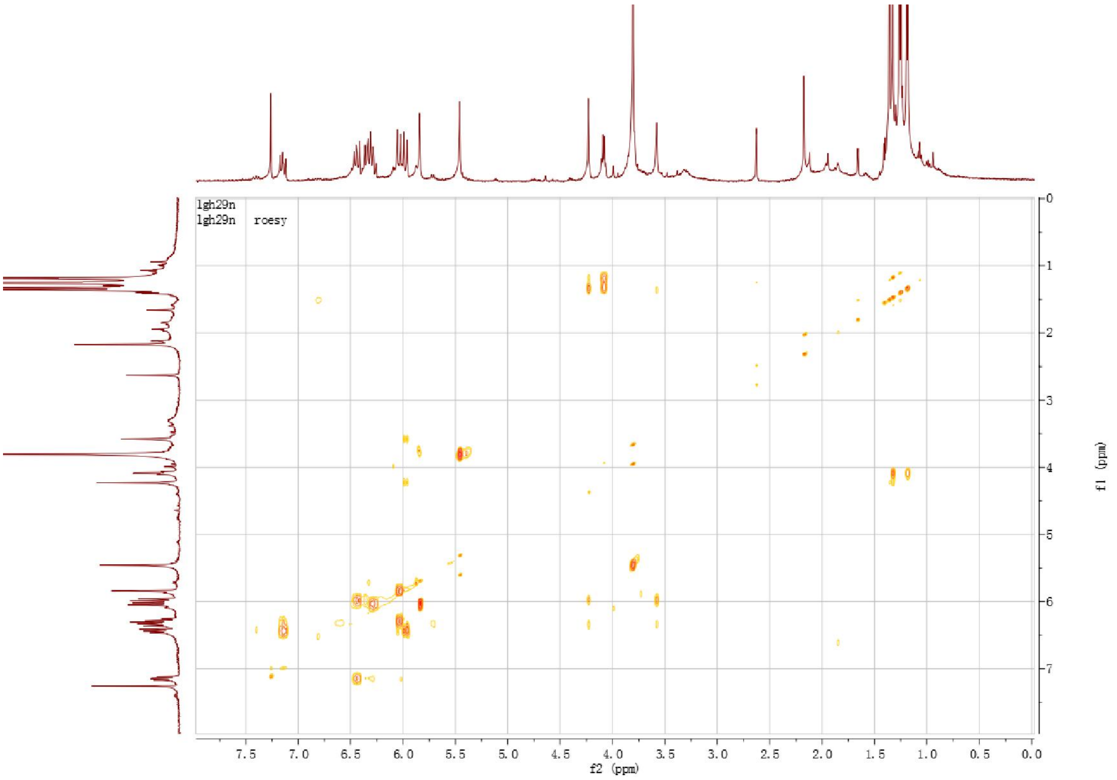

Figure 14S. HREIMS of aurovertin K (**2**).

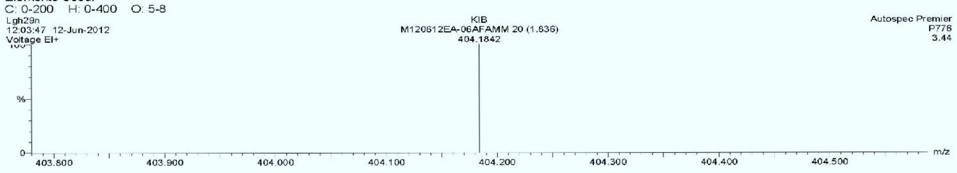

Elemental Composition Report **Page 1**

Single Mass Analysis
Tolerance = 10.0 PPM / DBE: min = -10.0, max = 120.0
Selected filters: None

Monoisotopic Mass, Odd and Even Electron Ions
24 formula(e) evaluated with 1 results within limits (up to 51 closest results for each mass)
Elements Used:
C: 0-200 H: 0-400 O: 5-8

Minimum:				-10.0		
Maximum:		140.0	10.0	120.0		
Mass	Calc. Mass	mDa	PPM	DBE	i-FIT	Formula
404.1842	404.1835	0.7	1.7	9.0	5546025.5	C22 H28 O7

Figure 15S. ^{1}H NMR of aurovertin L (**3**).

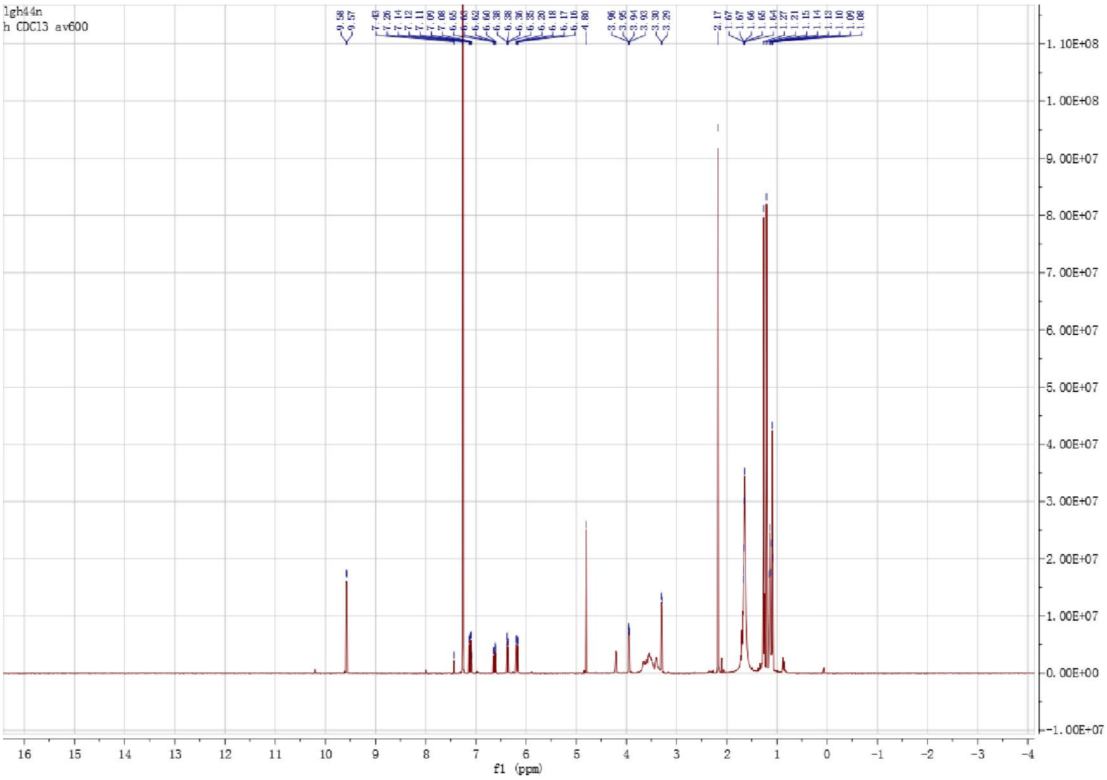

Figure 16S. ^{13}C NMR of aurovertin L (**3**).

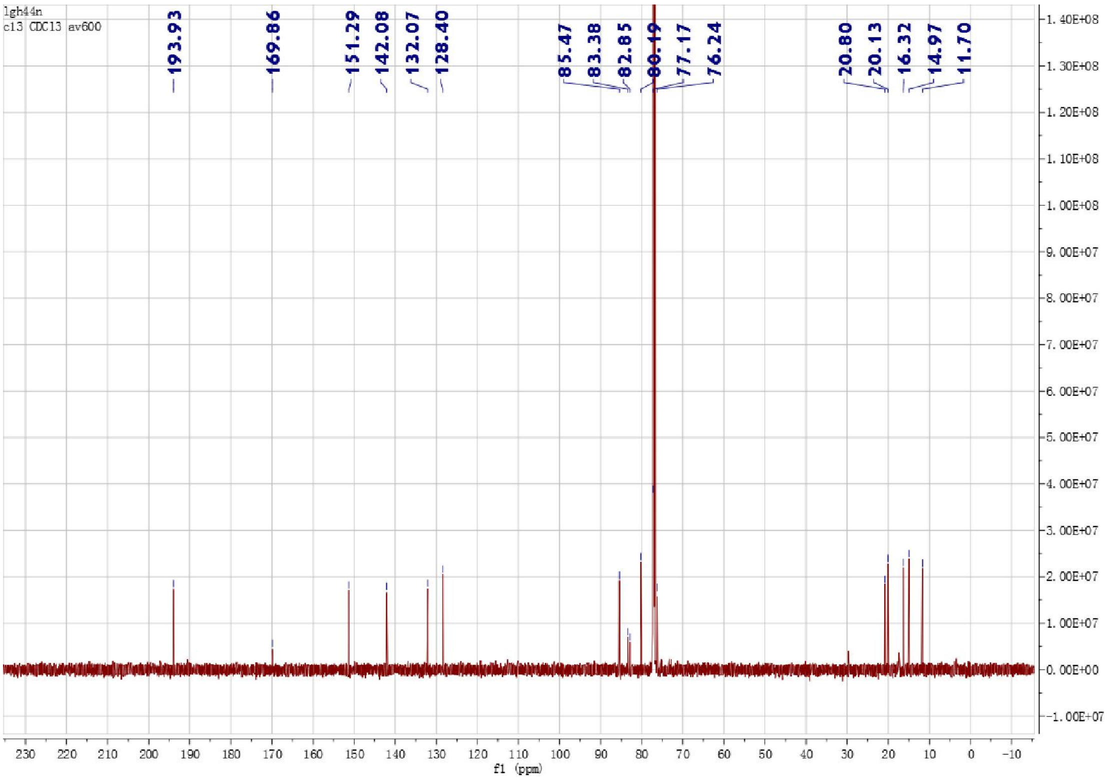

Figure 17S. HSQC of aurovertin L (**3**).

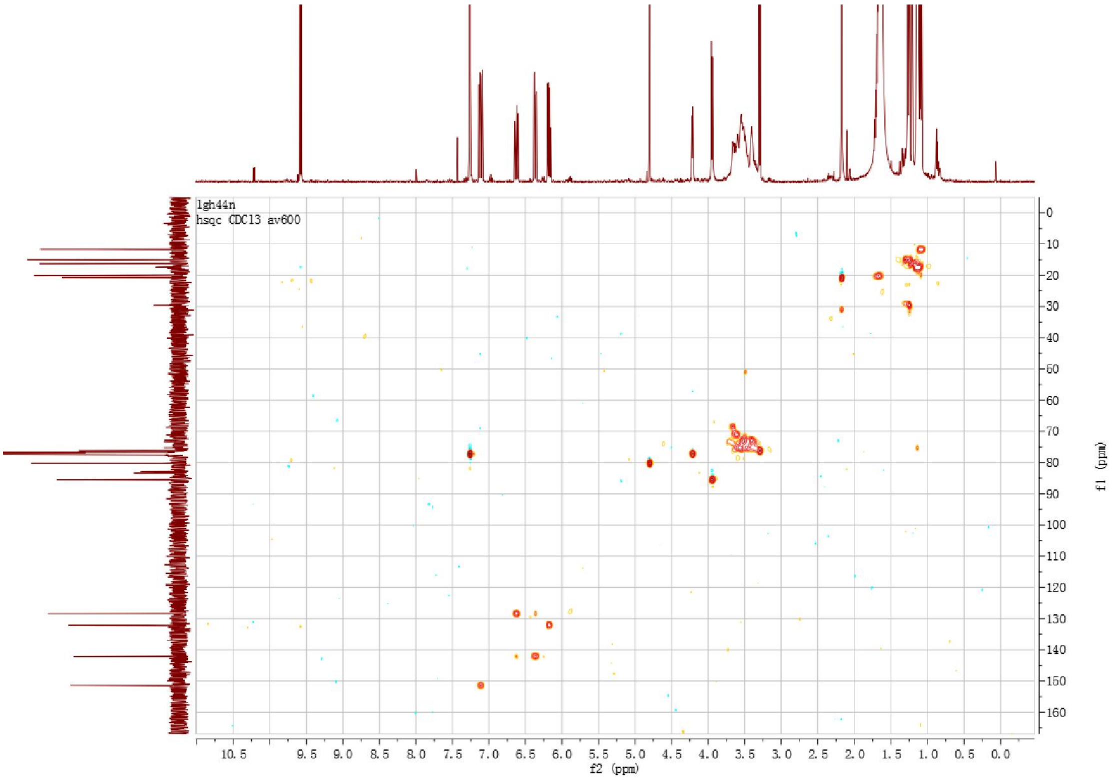

Figure 18S. HMBC of aurovertin L (**3**).

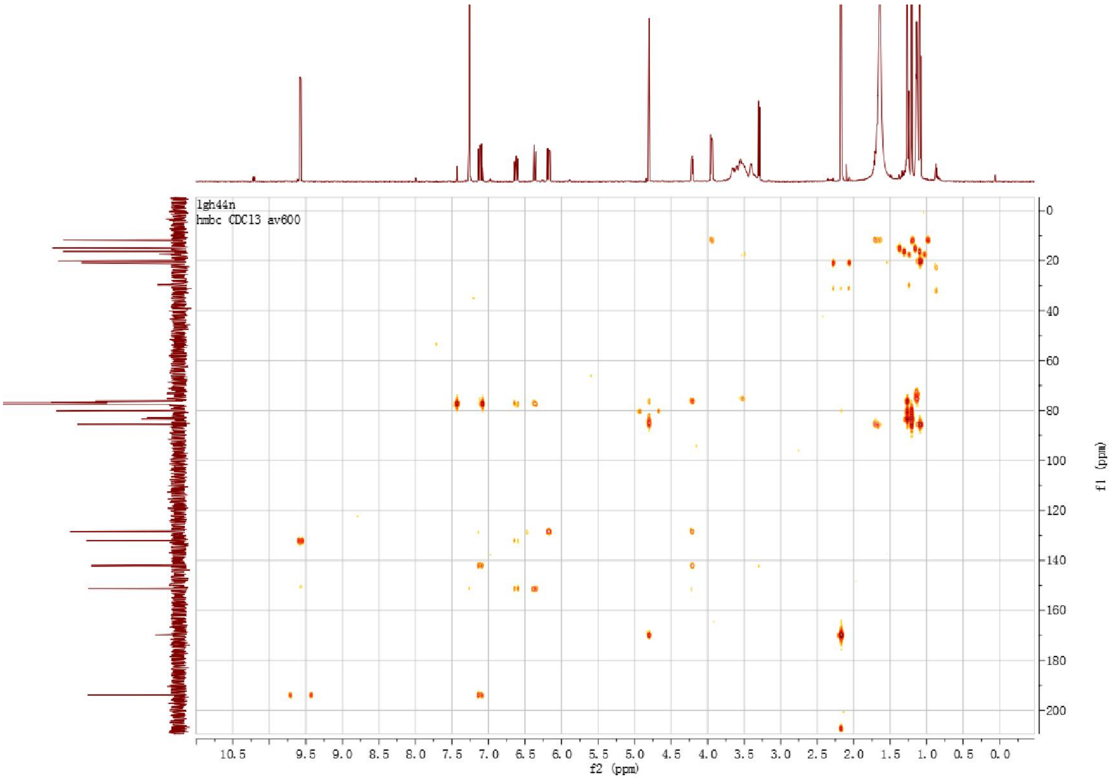

Figure 19S. ^{1}H-^{1}H COSY of aurovertin L (**3**).

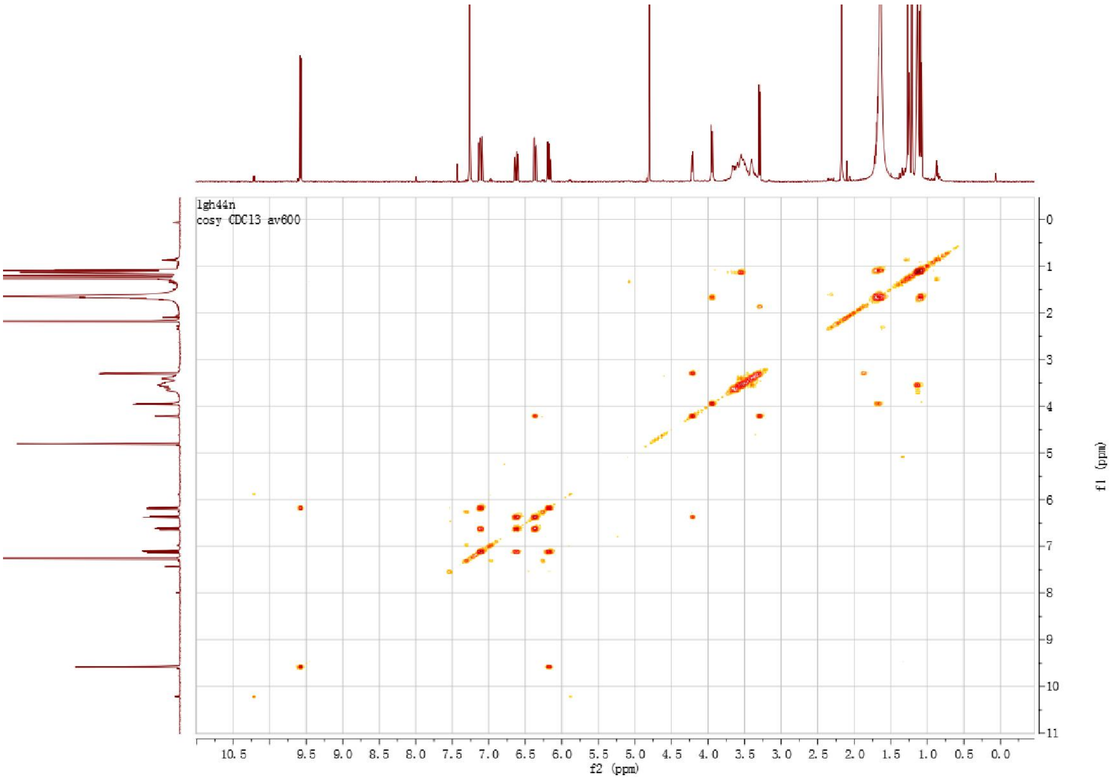

Figure 20S. ROESY of aurovertin L (**3**).

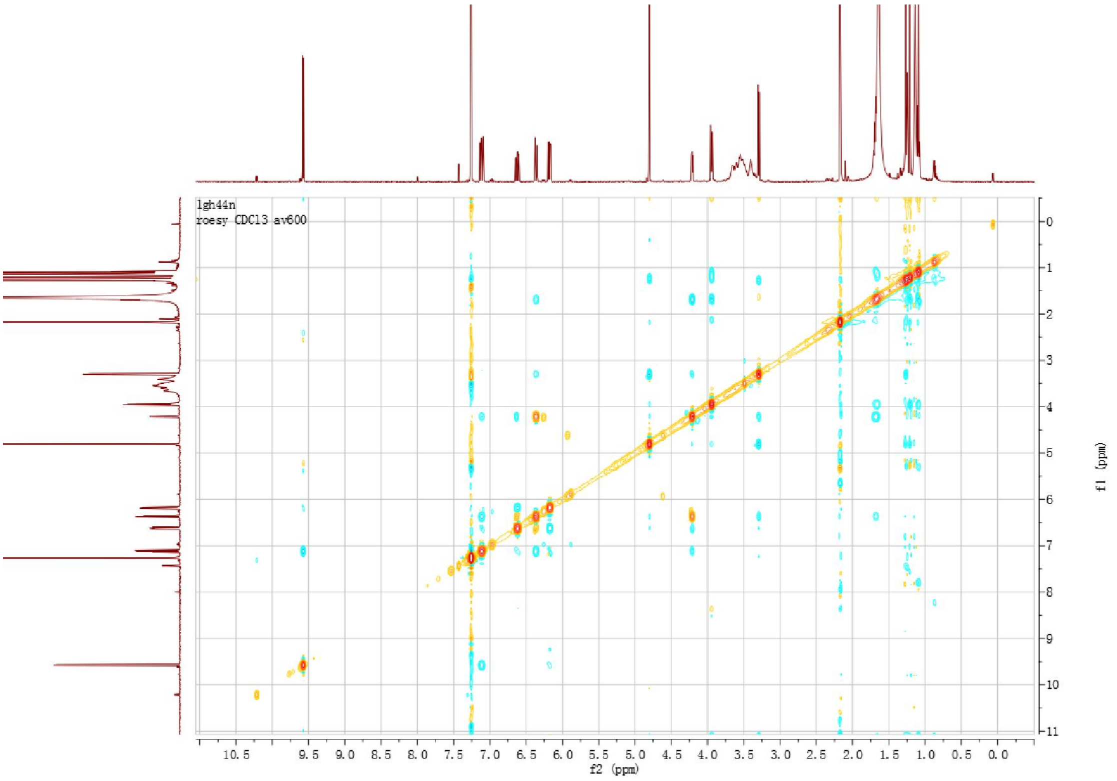

Figure 21S. HREIMS of aurovertin L (**3**).

Elemental Composition Report Page 1

Single Mass Analysis
Tolerance = 10.0 PPM / DBE: min = -10.0, max = 120.0
Selected filters: None

Monoisotopic Mass, Odd and Even Electron Ions
19 formula(e) evaluated with 1 results within limits (up to 51 closest results for each mass)
Elements Used:
C: 0-200 H: 0-400 O: 4-7

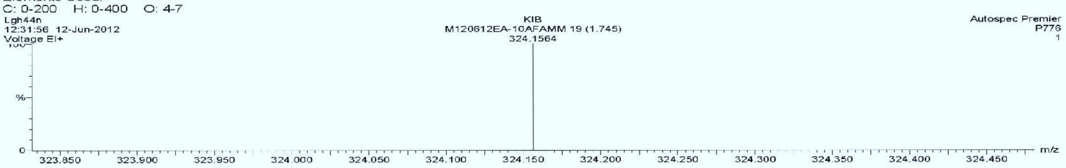

Minimum:				-10.0		
Maximum:		140.0	10.0	120.0		
Mass	Calc. Mass	mDa	PPM	DBE	i-FIT	Formula
324.1564	324.1573	-0.9	-2.8	6.0	5546026.0	C17 H24 O6

Figure 22S. ^{1}H NMR of aurovertin M (**4**).

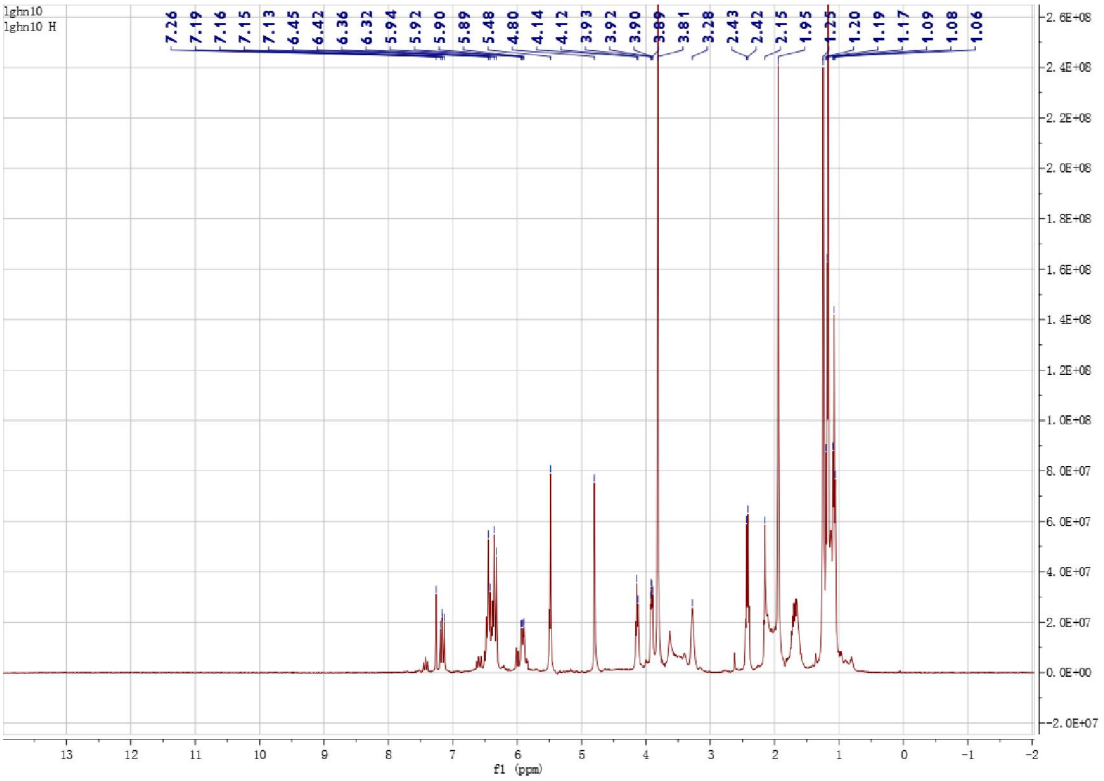

Figure 23S. ^{13}C NMR of aurovertin M (**4**).

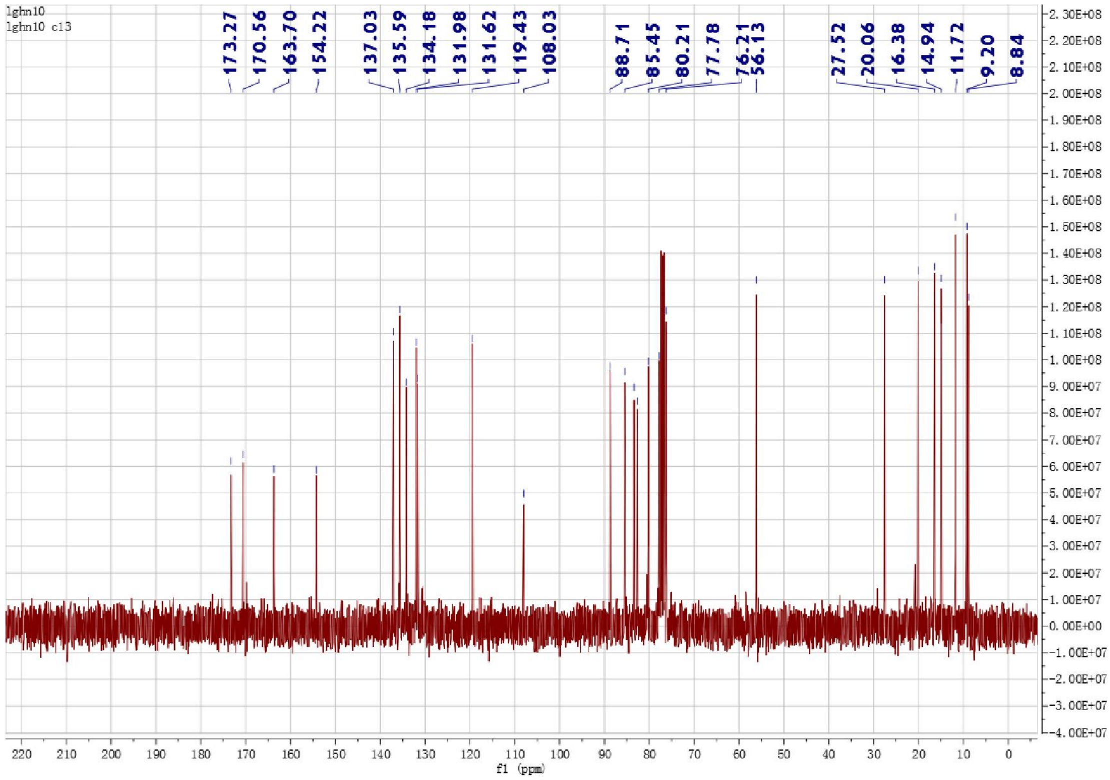

Figure 24S. HSQC of aurovertin M (**4**).

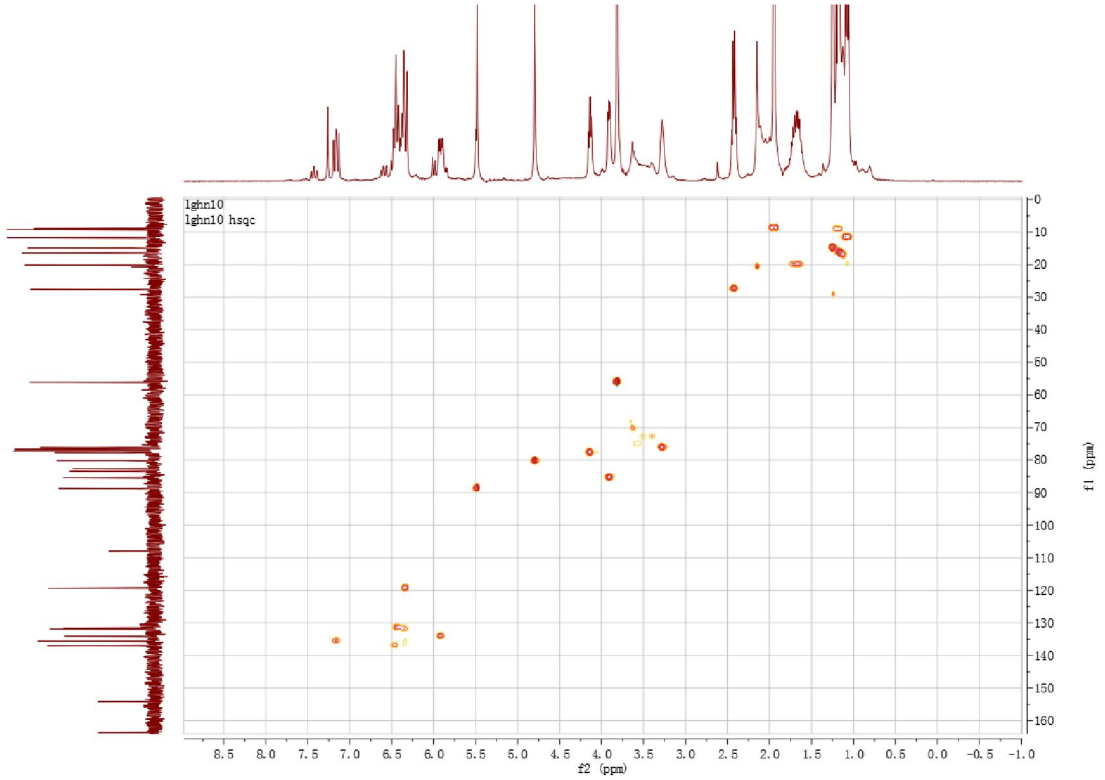

Figure 25S. HMBC of aurovertin M (**4**).

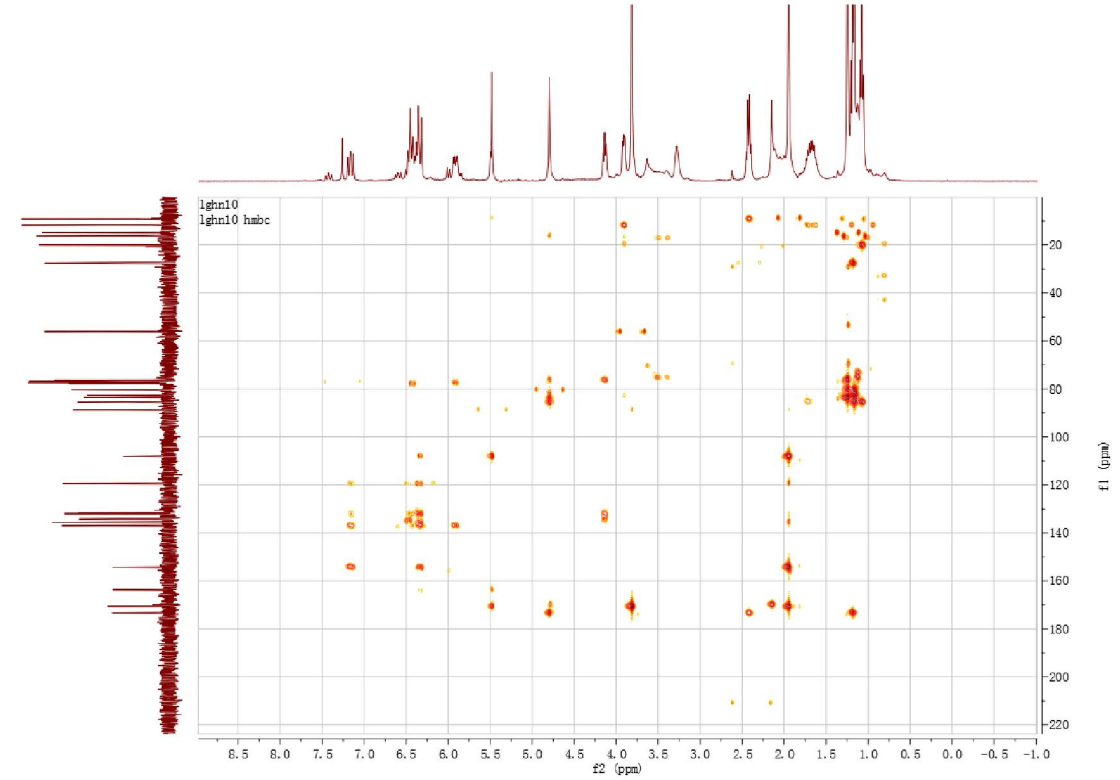

Figure 26S. ^{1}H-^{1}H COSY of aurovertin M (**4**).

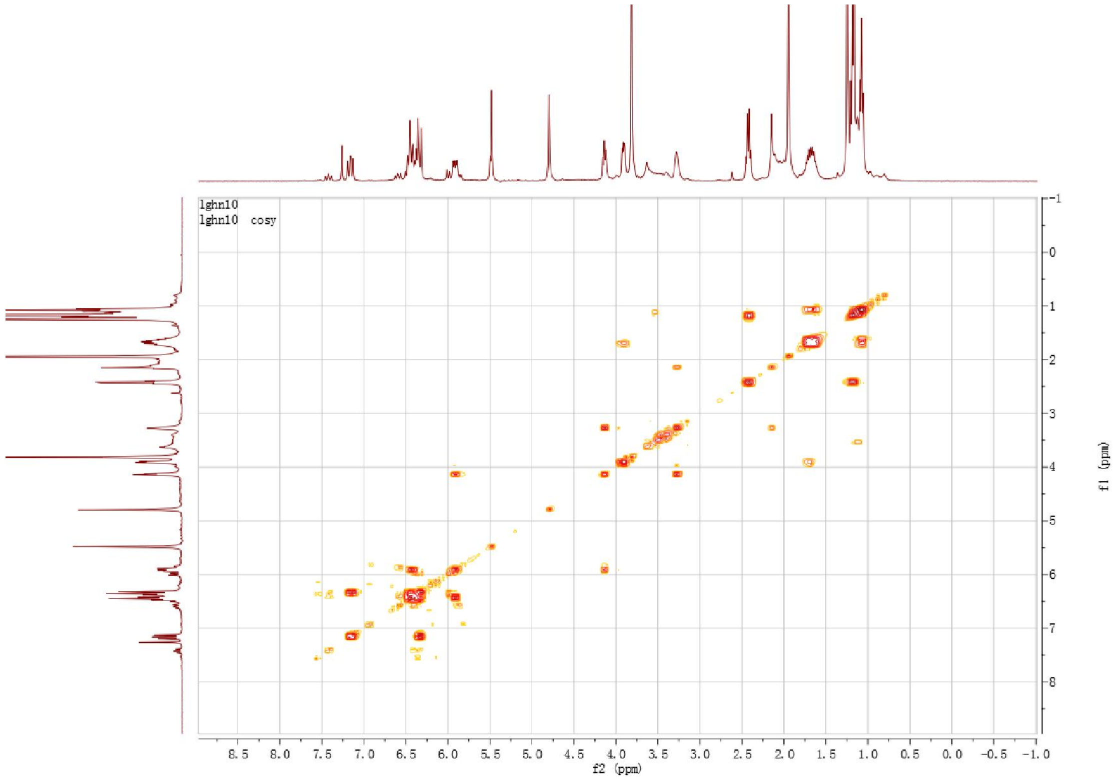

Figure 27S. ROESY of aurovertin M (**4**).

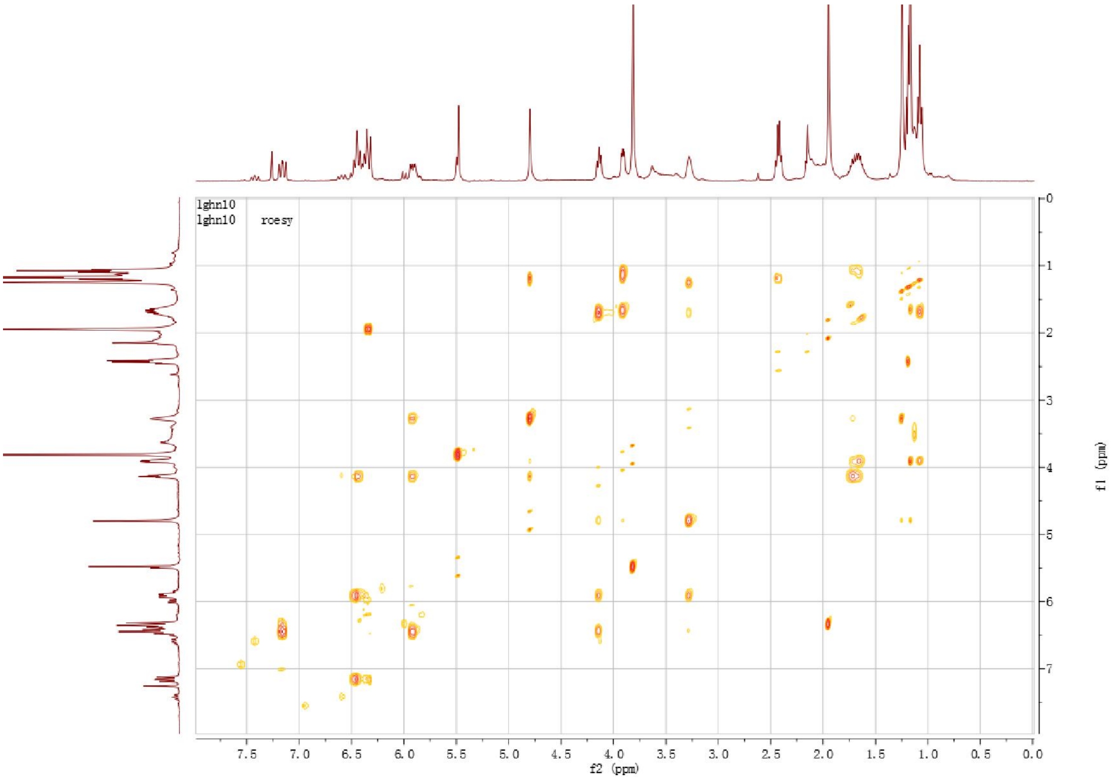

Figure 28S. HREIMS of aurovertin M (**4**).

Elemental Composition Report **Page 1**

Single Mass Analysis
Tolerance = 10.0 PPM / DBE: min = -10.0, max = 120.0
Selected filters: None

Monoisotopic Mass, Odd and Even Electron Ions
32 formula(e) evaluated with 1 results within limits (up to 51 closest results for each mass)
Elements Used:
C: 0-200 H: 0-400 O: 6-10

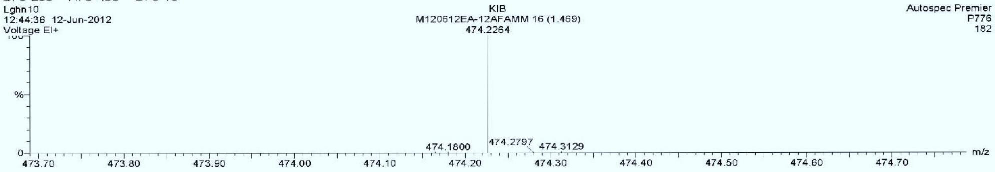

Minimum:				-10.0		
Maximum:		[illegible]	10.0	120.0		
Mass	Calc. Mass	mDa	PPM	DBE	i-FIT	Formula
474.2264	474.2254	1.0	2.1	10.0	5546108.0	C26 H34 O8

Figure 29S. ^{1}H NMR of aurovertin N (**5**).

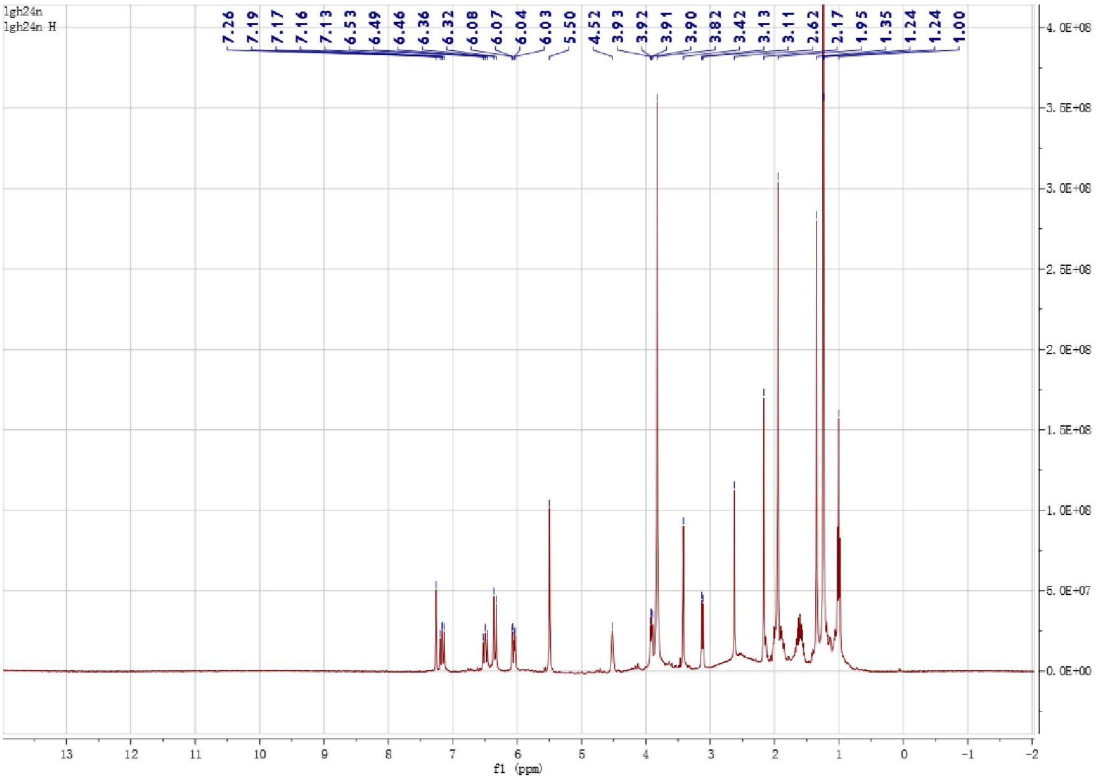

Figure 30S. ^{13}C NMR of aurovertin N (**5**).

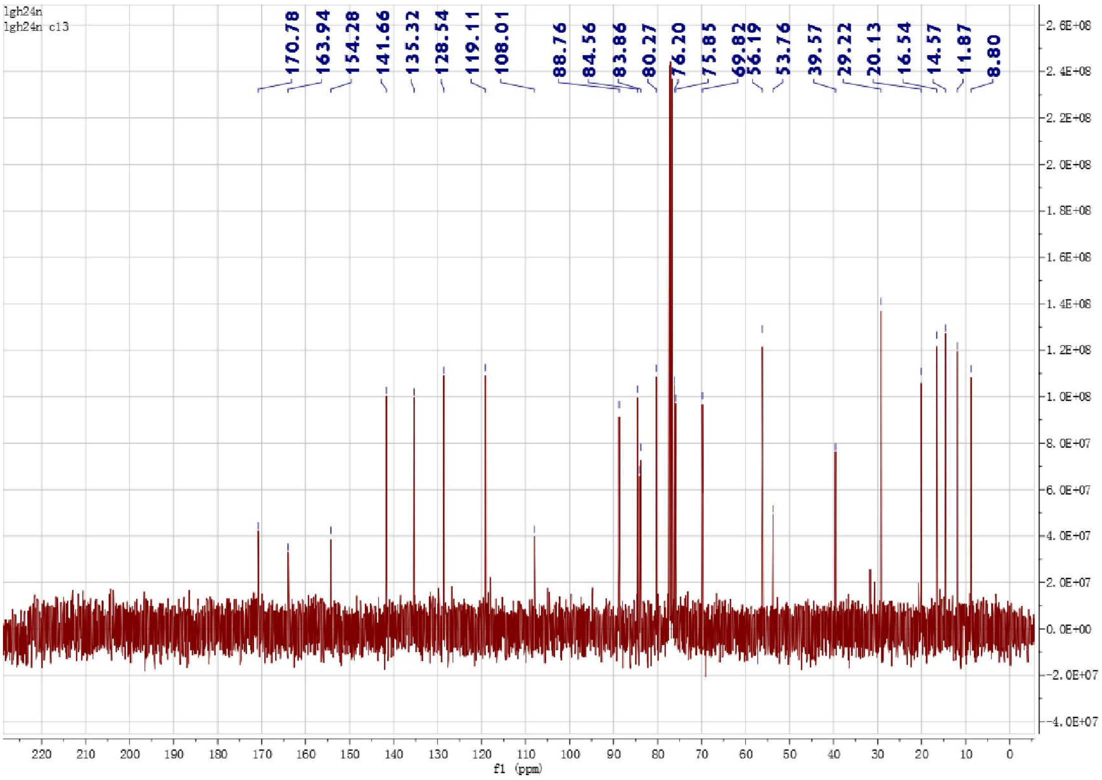

Figure 31S. HSQC of aurovertin N (**5**).

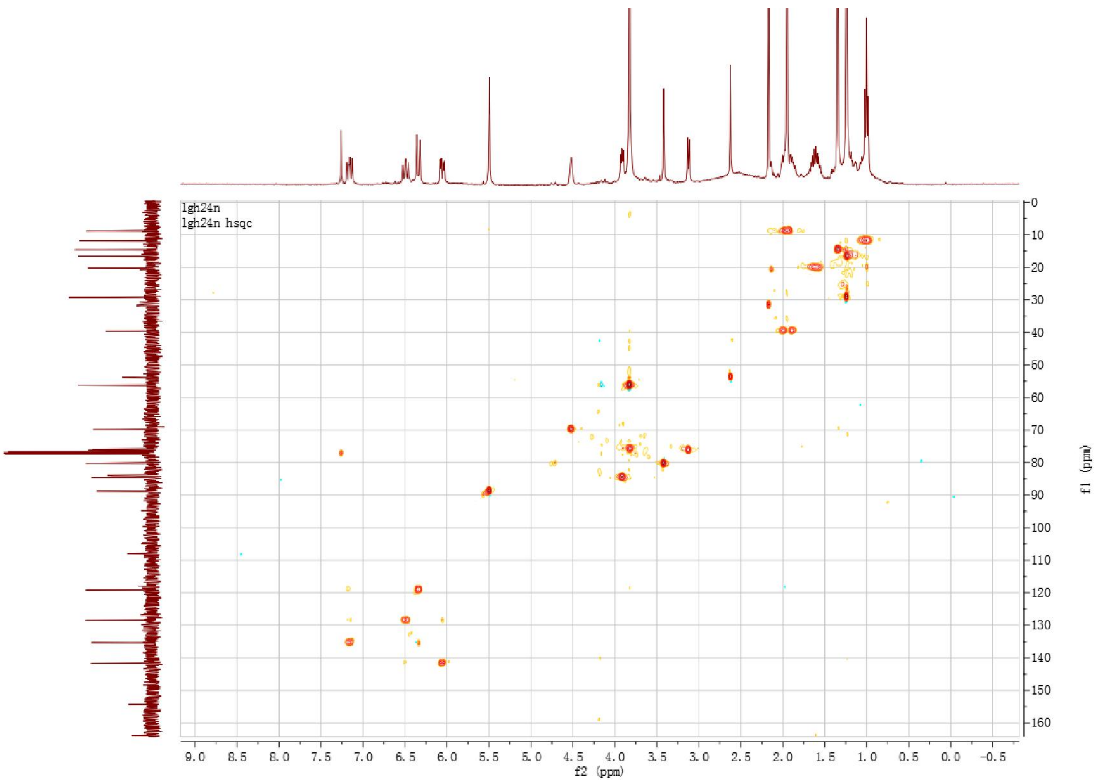

Figure 32S. HMBC aurovertin N (**5**)

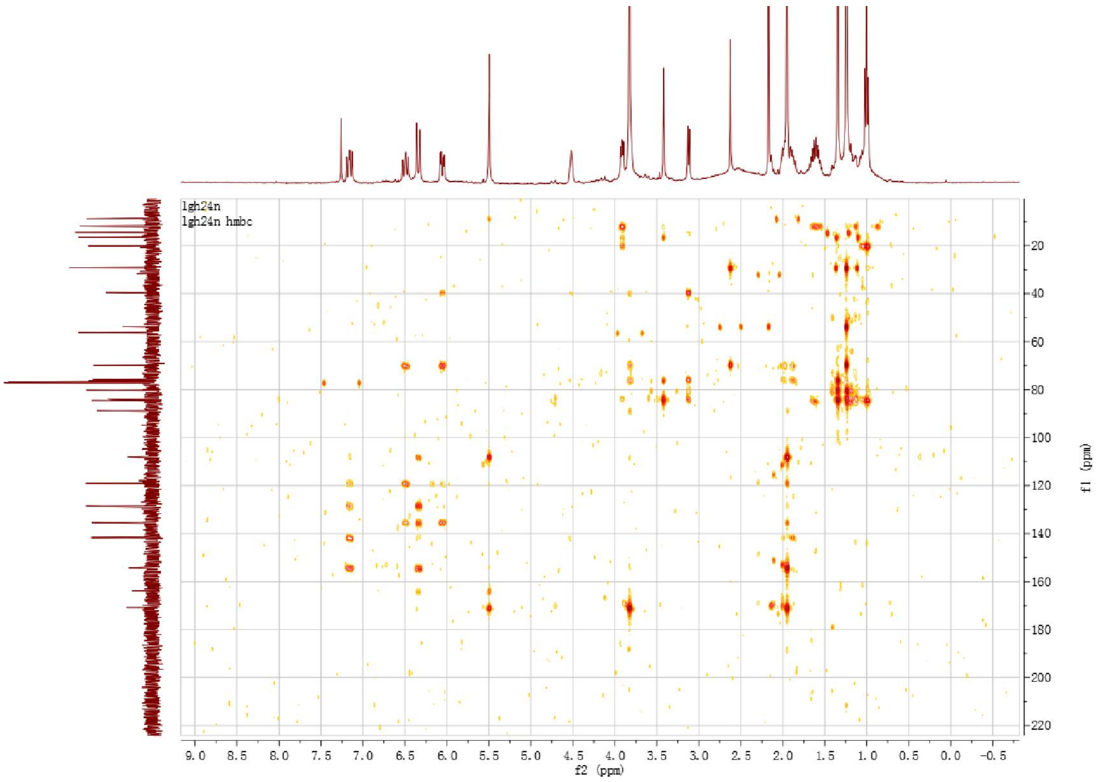

Figure 33S. ^1H-^1H COSY of aurovertin N (**5**).

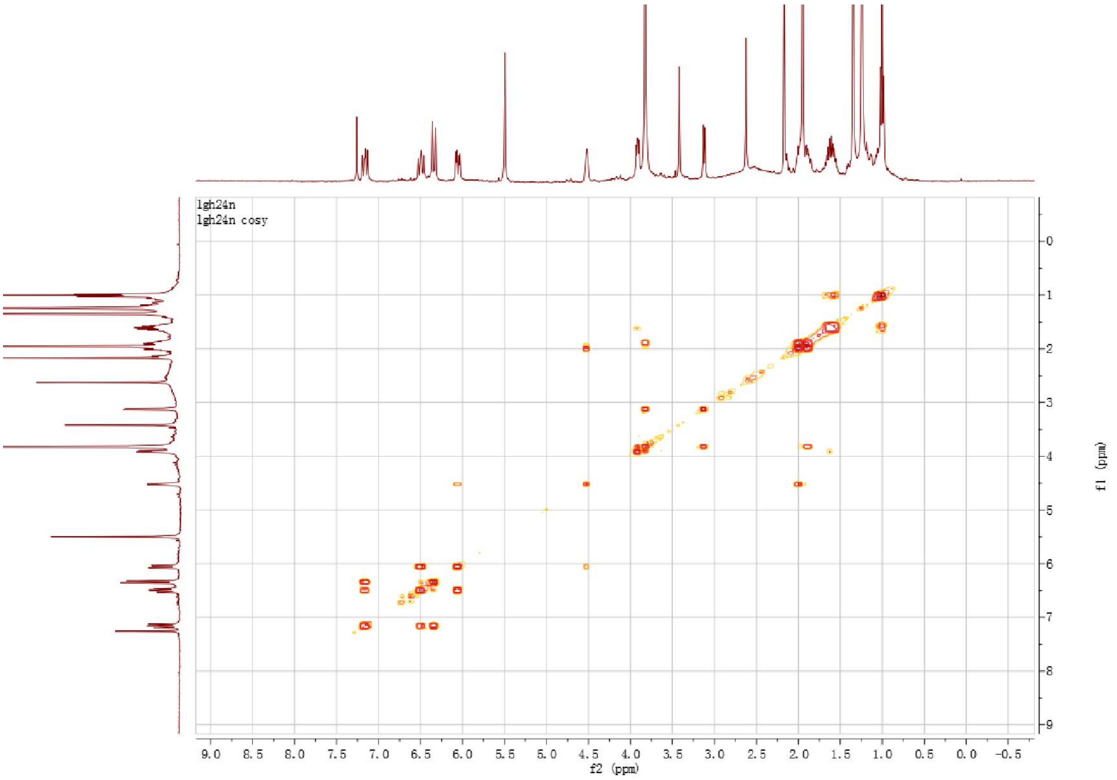

Figure 34S. ROESY of aurovertin N (**5**).

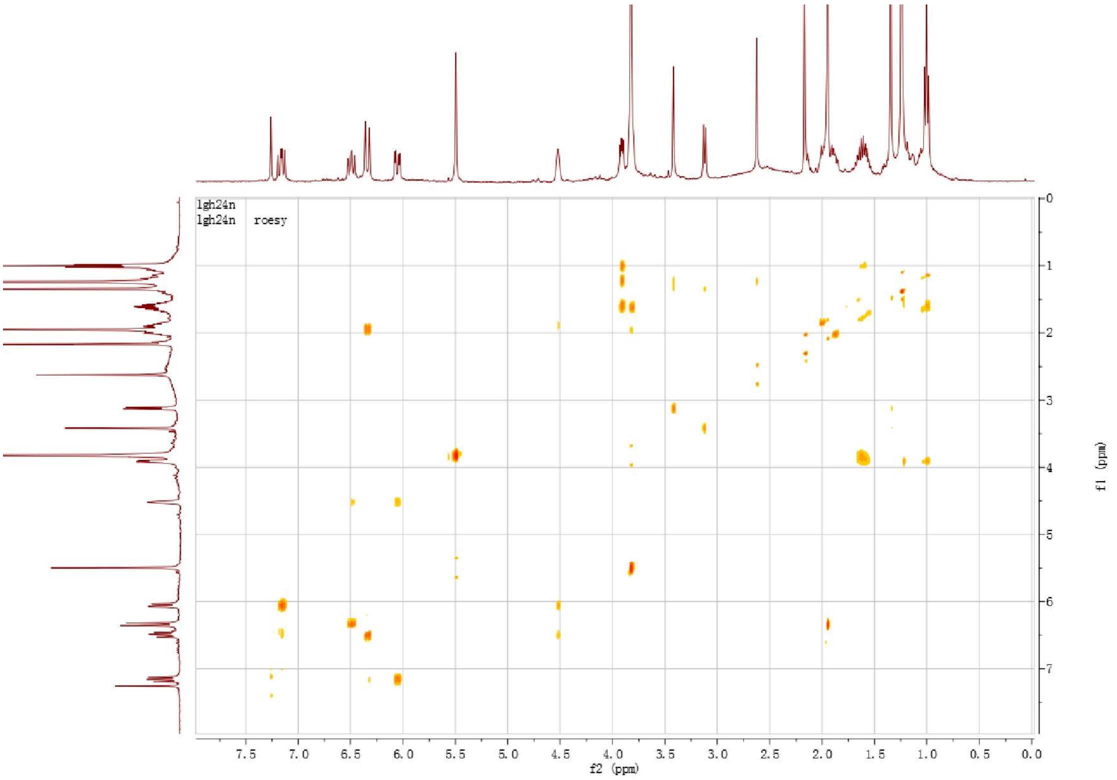

Figure 35S. HREIMS of aurovertin N (**5**).

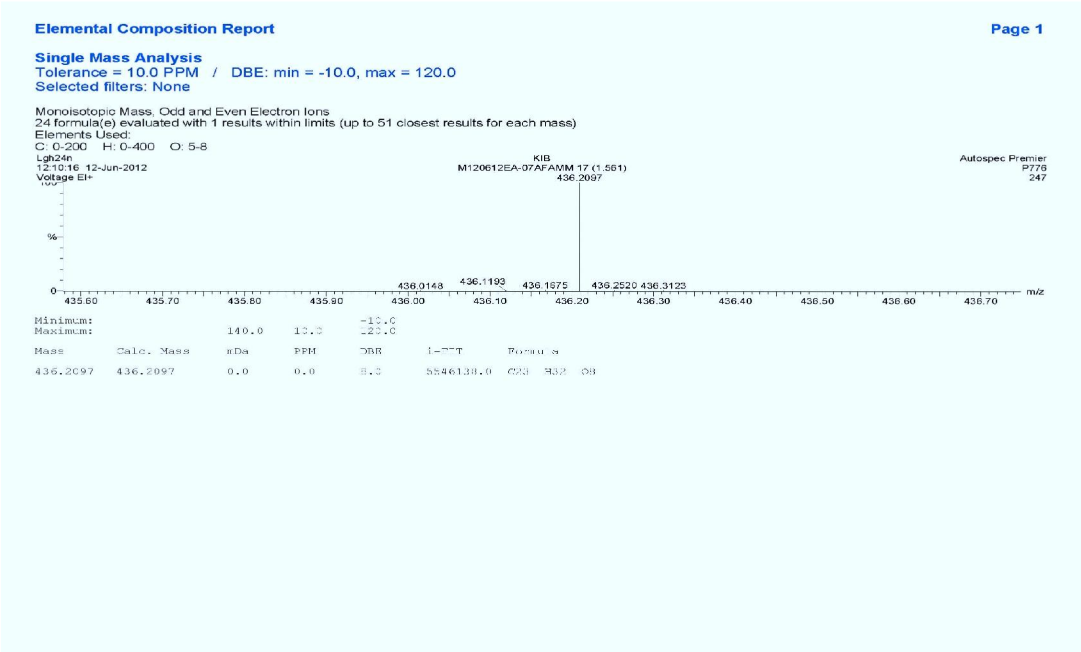

Figure 36S. ^{1}H NMR of aurovertin O (**6**).

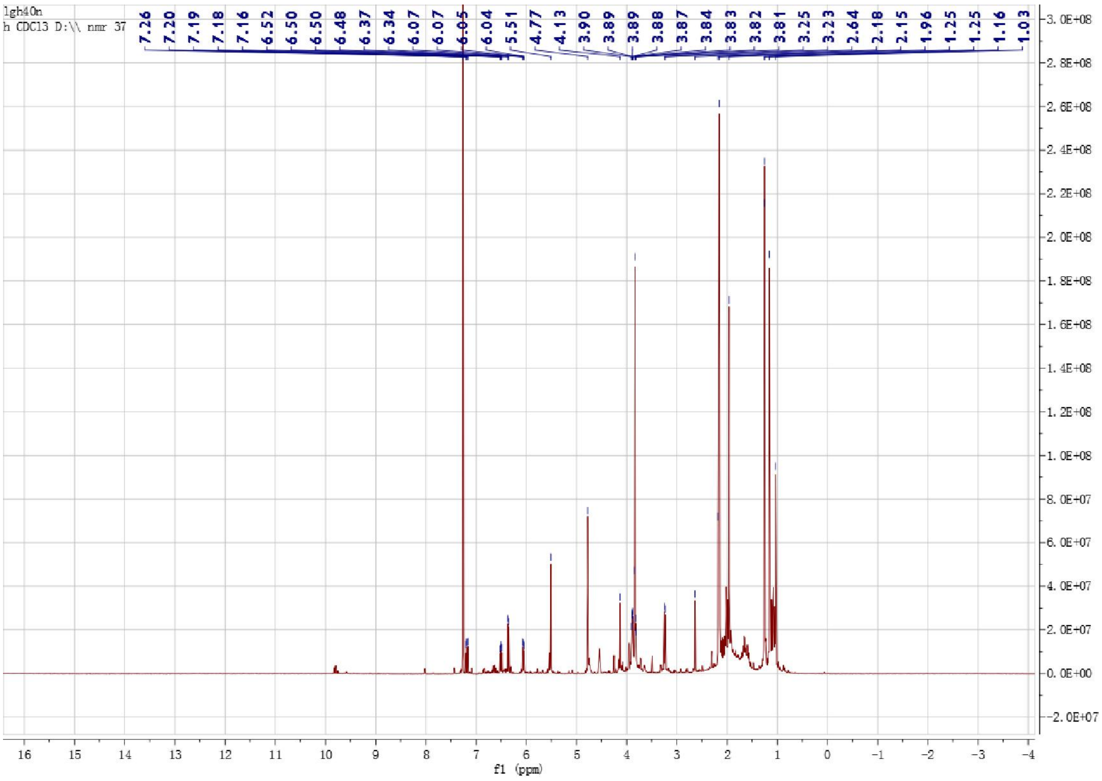

Figure 37S. ^{13}C NMR of aurovertin O (**6**).

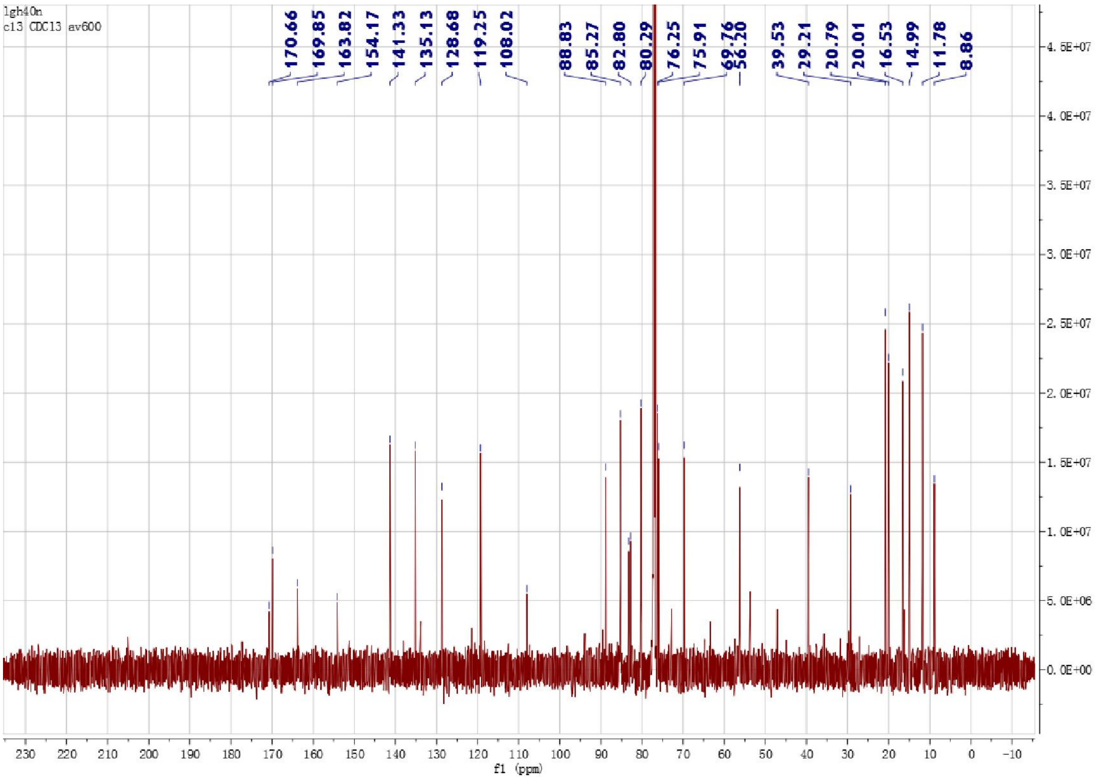

Figure 38S. HSQC of aurovertin O (**6**).

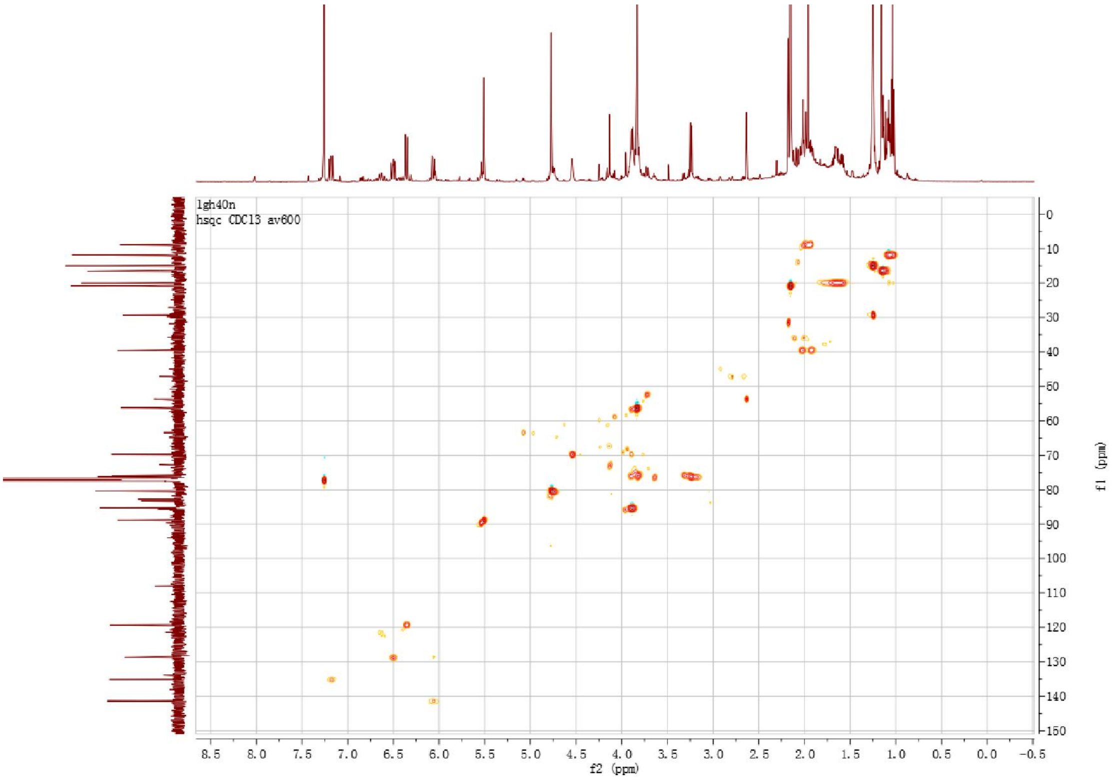

Figure 39S. HMBC aurovertin O (**6**)

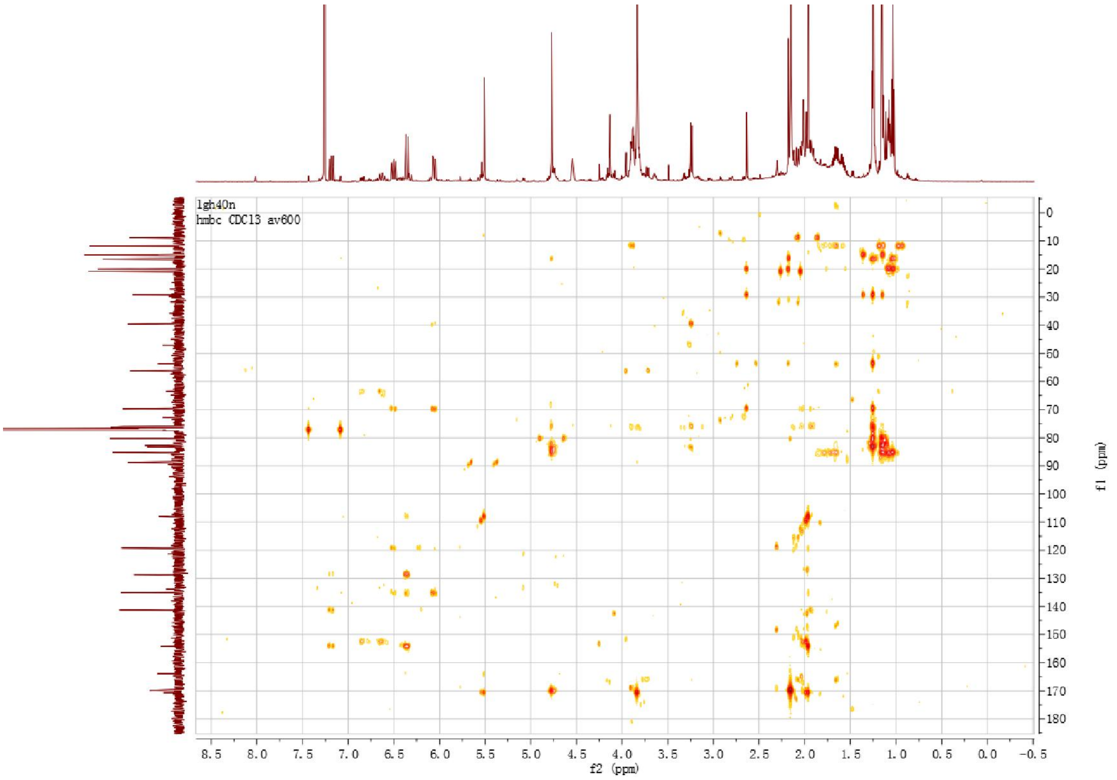

Figure 40S. ^{1}H-^{1}H COSY of aurovertin O (**6**).

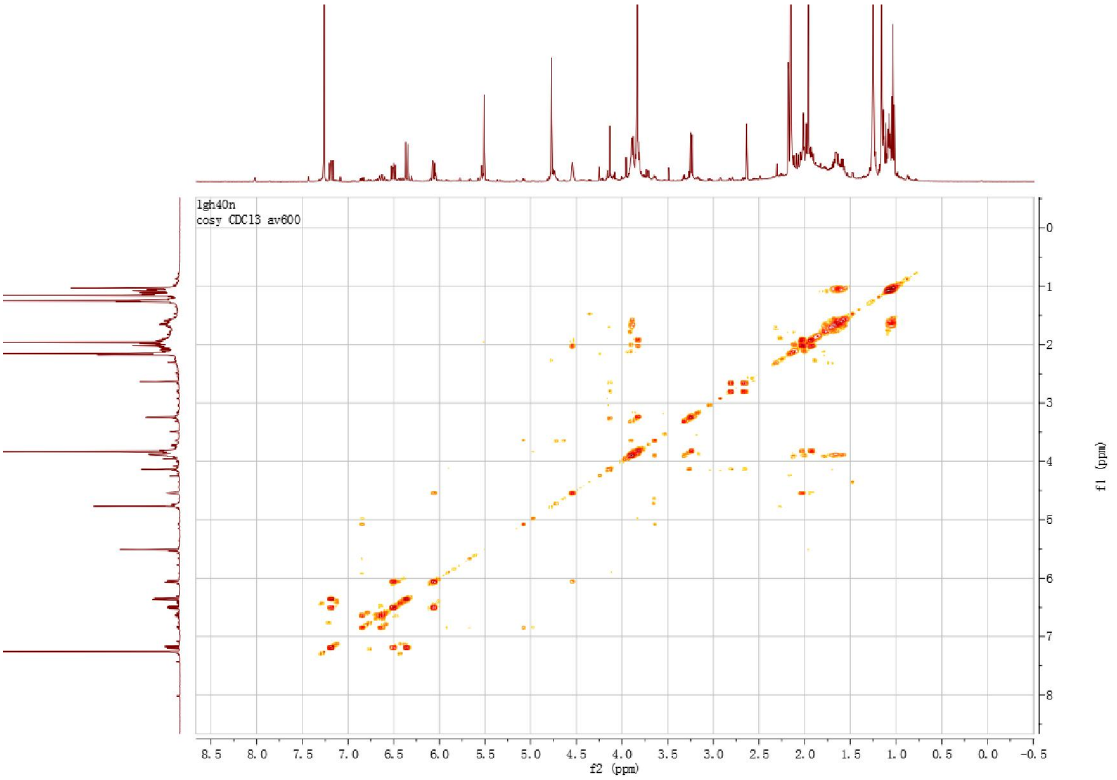

Figure 41S. ROESY of aurovertin O (**6**).

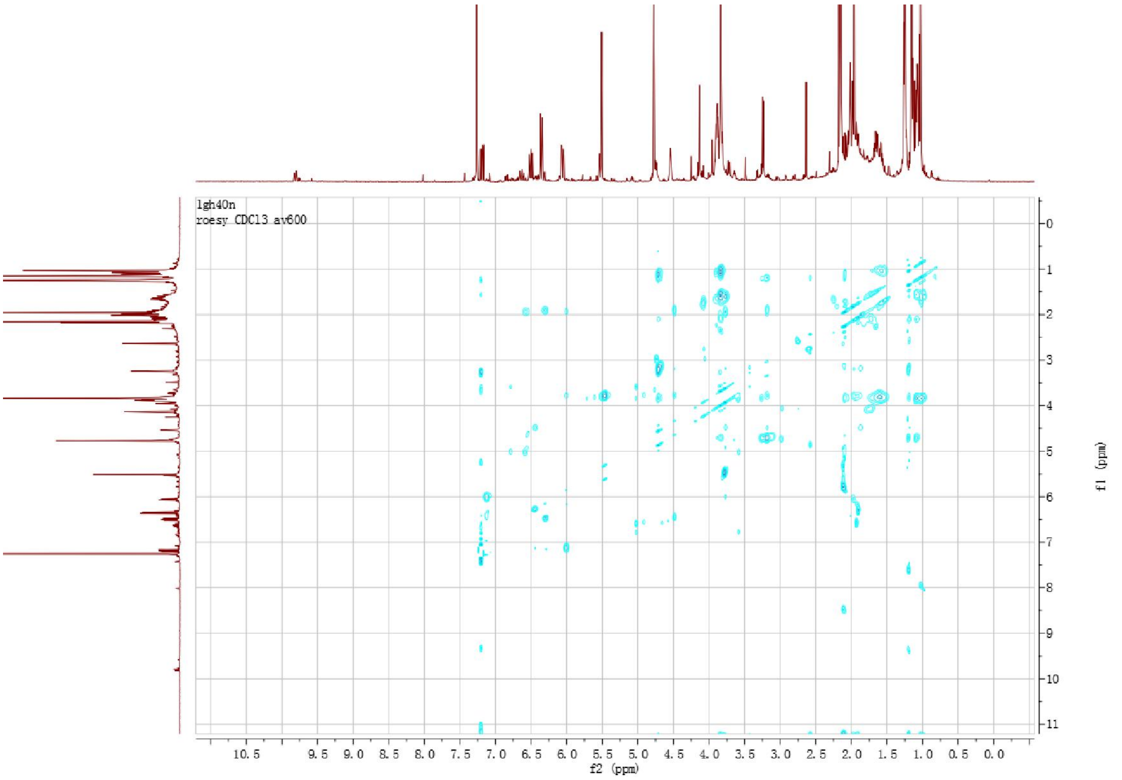

Figure 42S. HREIMS of aurovertin O (**6**).

Elemental Composition Report **Page 1**

Single Mass Analysis
Tolerance = 10.0 PPM / DBE: min = -10.0, max = 120.0
Selected filters: None

Monoisotopic Mass, Odd and Even Electron Ions
31 formula(e) evaluated with 1 results within limits (up to 51 closest results for each mass)
Elements Used:
C: 0-200 H: 0-400 O: 6-10

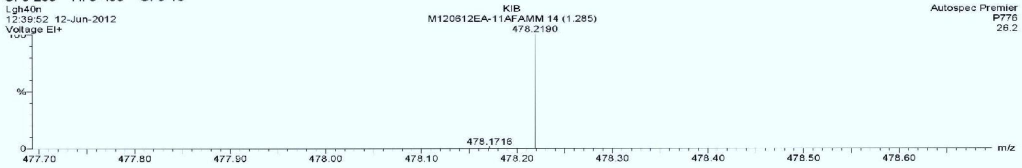

Minimum:				-10.0		
Maximum:		140.0	10.0	120.0		
Mass	Calc. Mass	mDa	PPM	DBE	i-FIT	Formula
478.2190	478.2203	-1.3	2.7	9.0	5546032.0	C25 H34 O9

Figure 43S. ^{1}H NMR of aurovertin P (**7**).

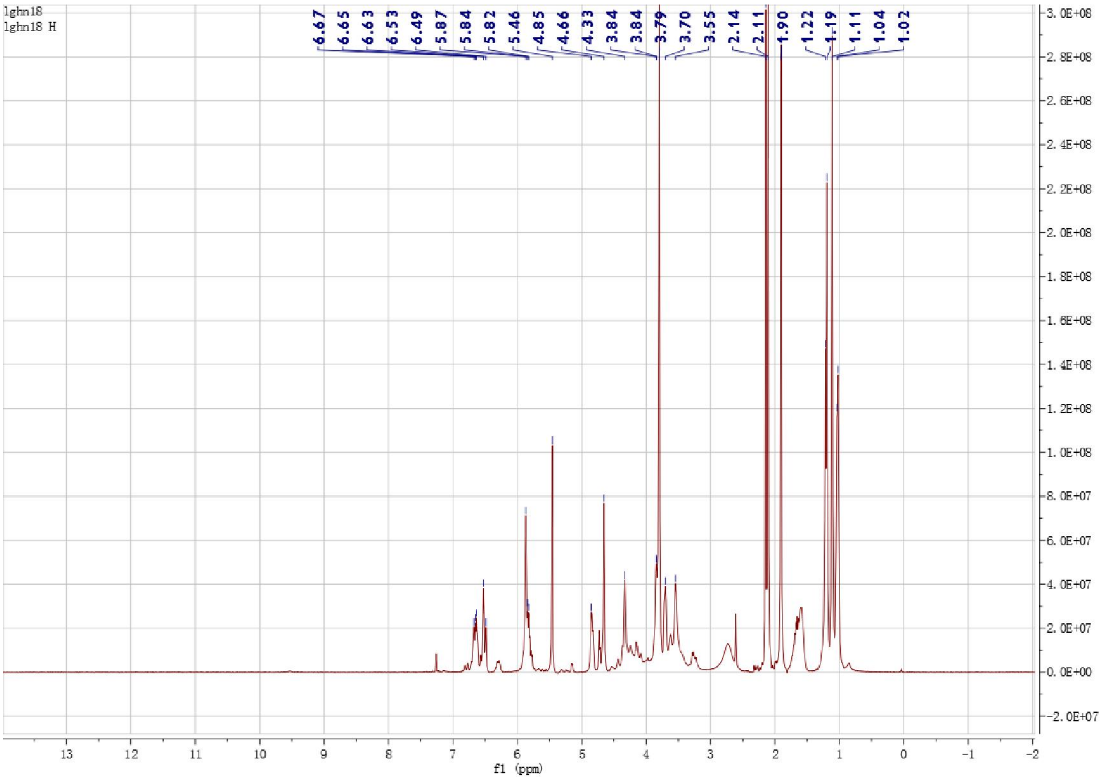

Figure 44S. ^{13}C NMR of aurovertin P (**7**).

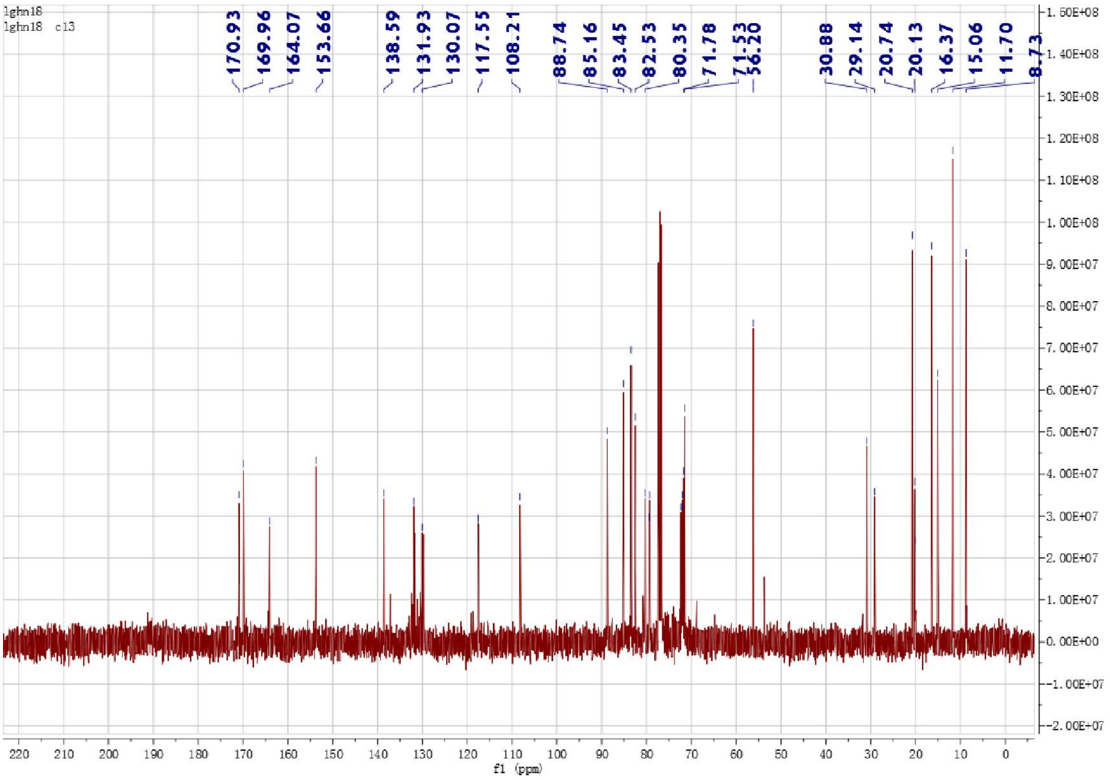

Figure 45S. HSQC of aurovertin P (**7**).

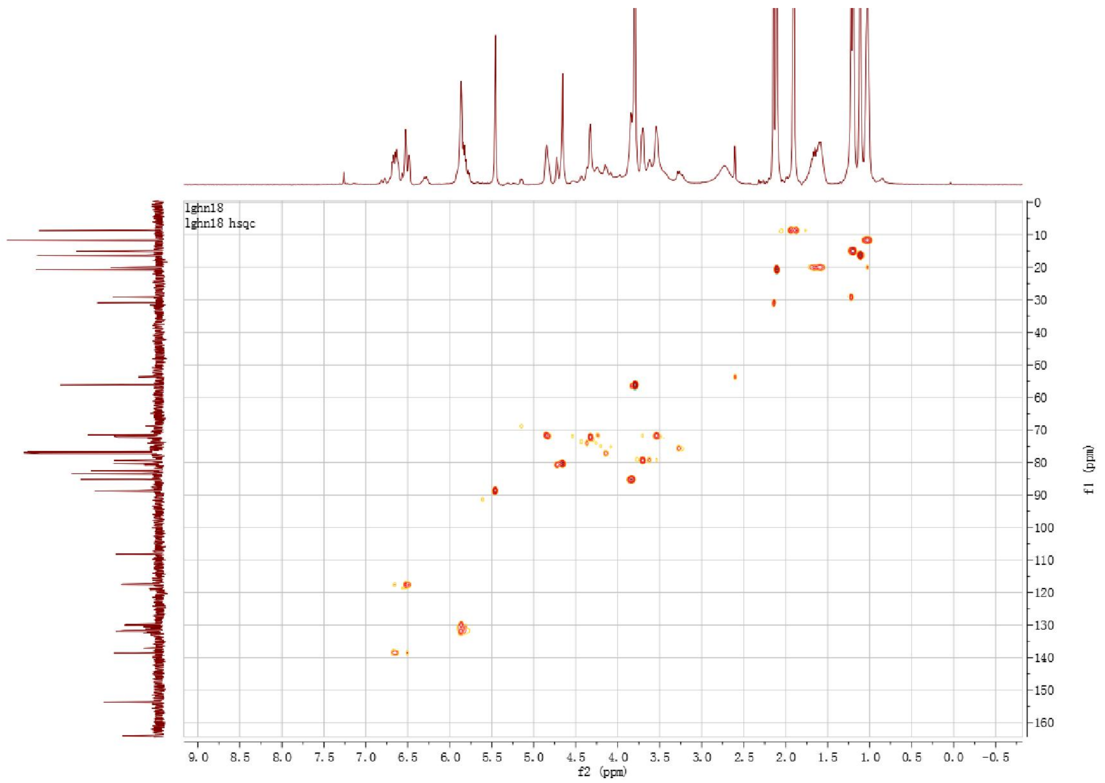

Figure 46S. HMBC of aurovertin P (**7**).

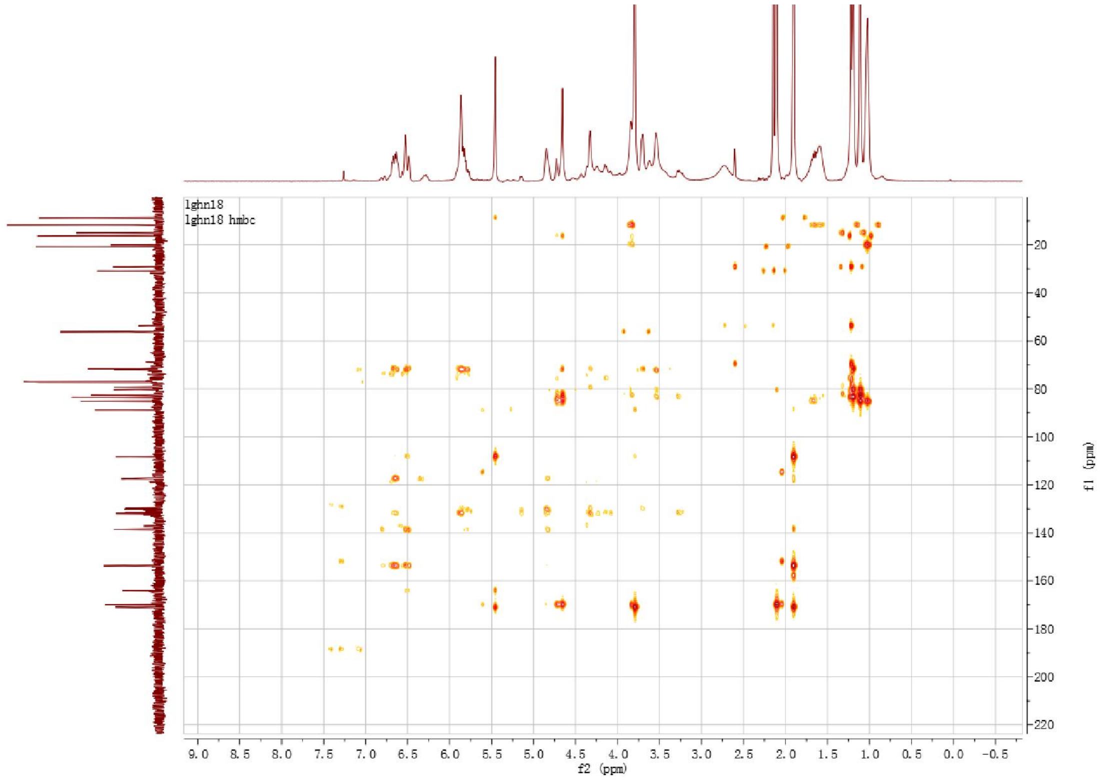

Figure 47S. ^{1}H-^{1}H COSY of aurovertin P (**7**).

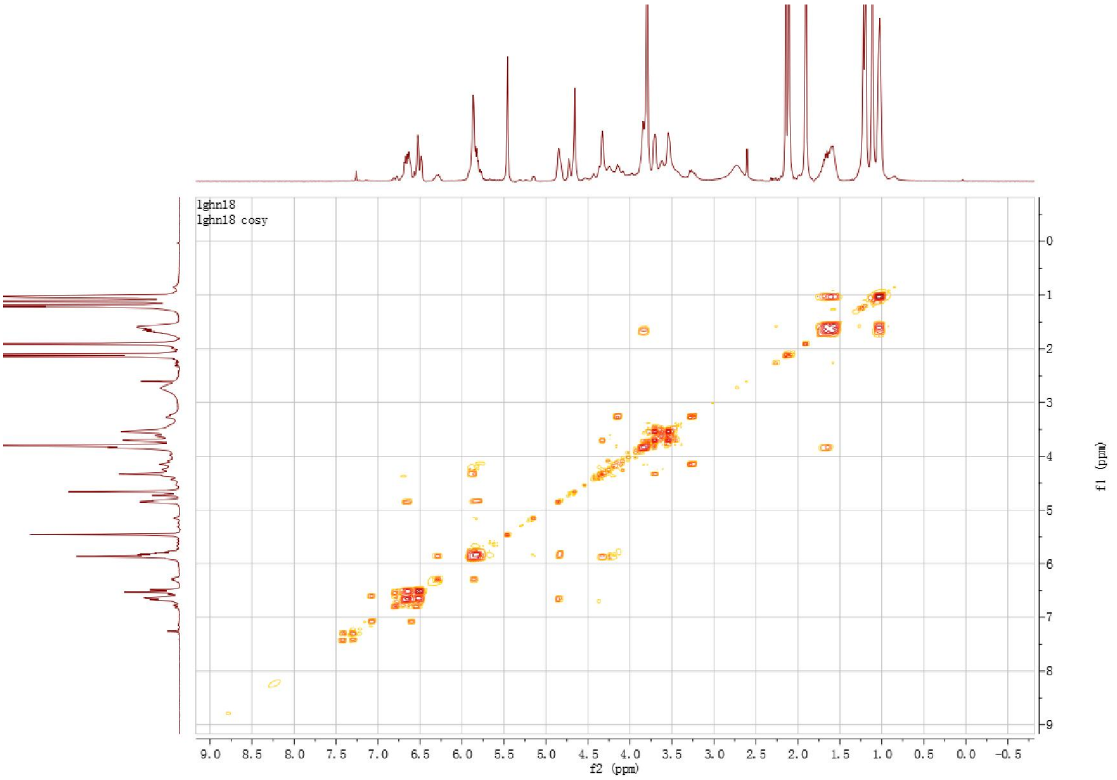

Figure 48S. ROESY of aurovertin P (**7**).

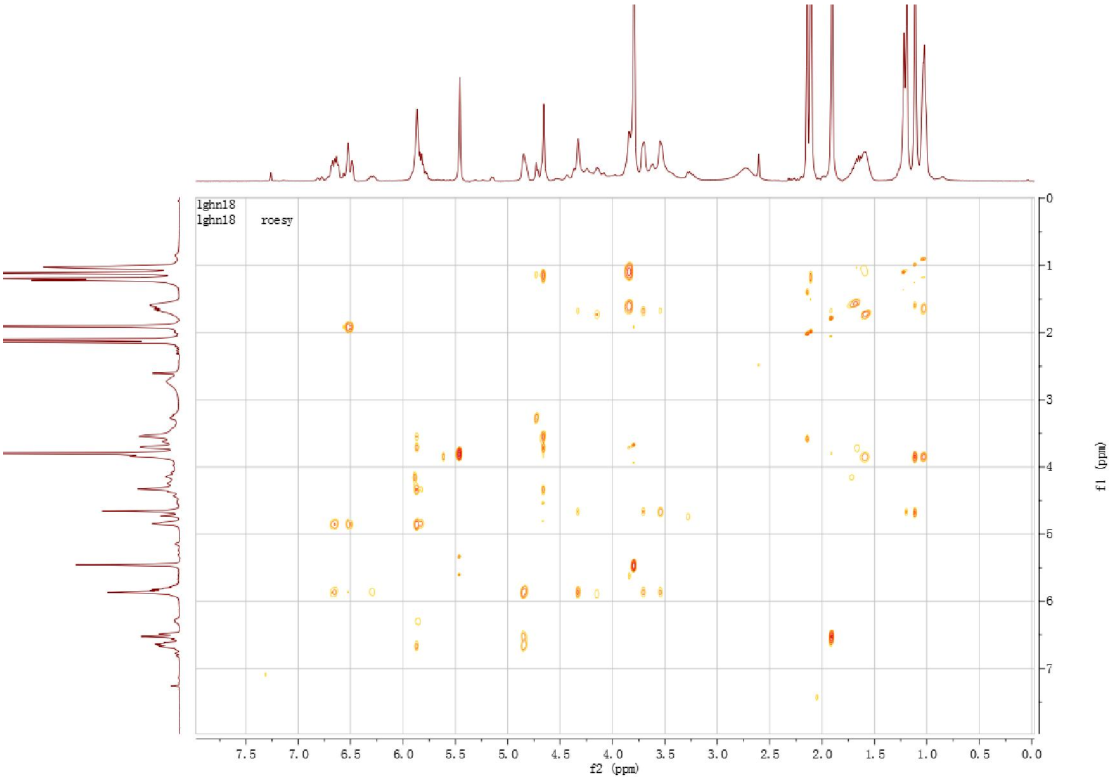

Figure 49S. HREIMS of aurovertin P (**7**).

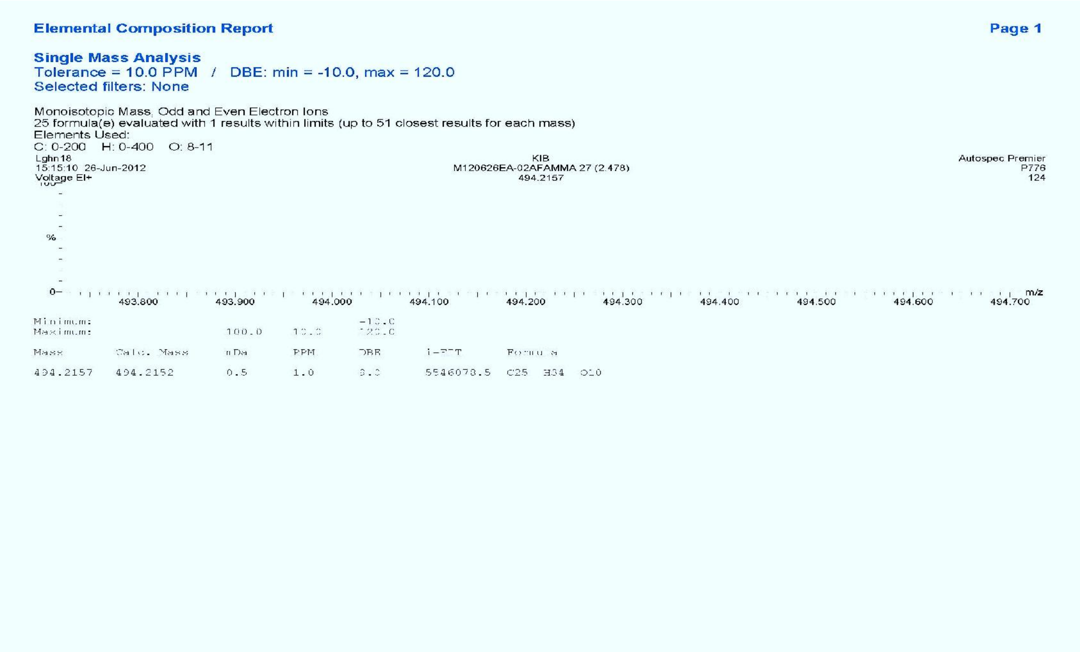

Figure 50S. ^{1}H NMR of aurovertin Q (**8**).

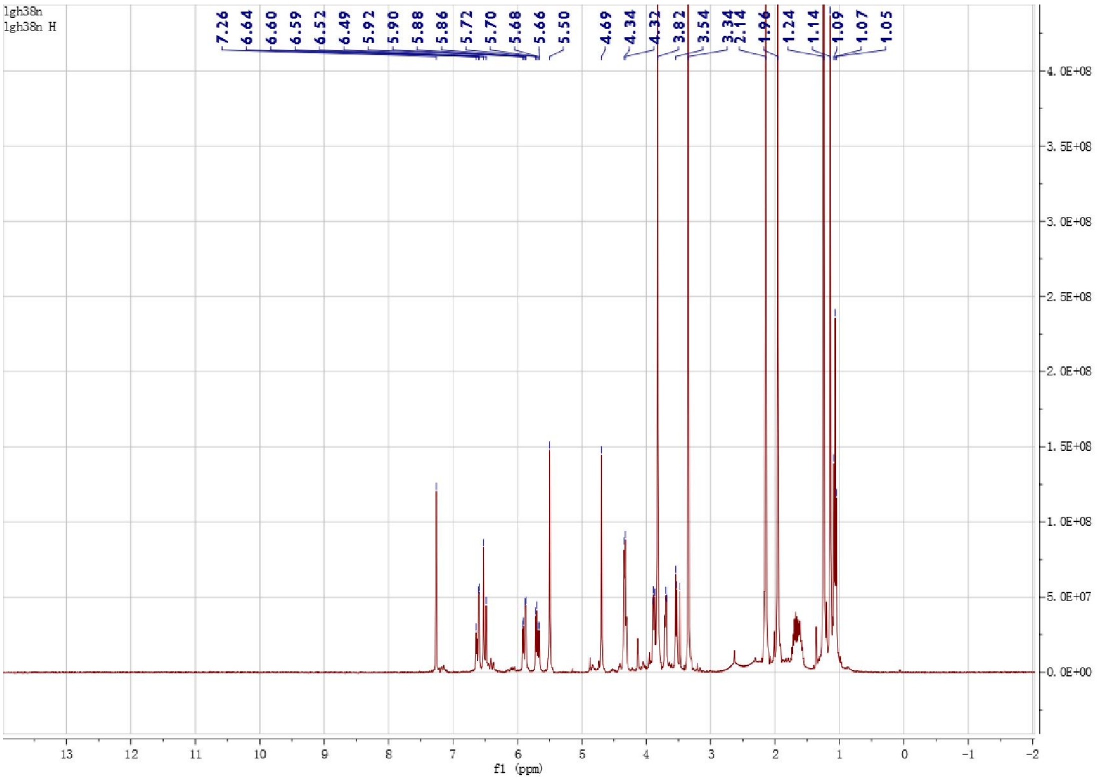

Figure 51S. ^{13}C NMR of aurovertin Q (**8**).

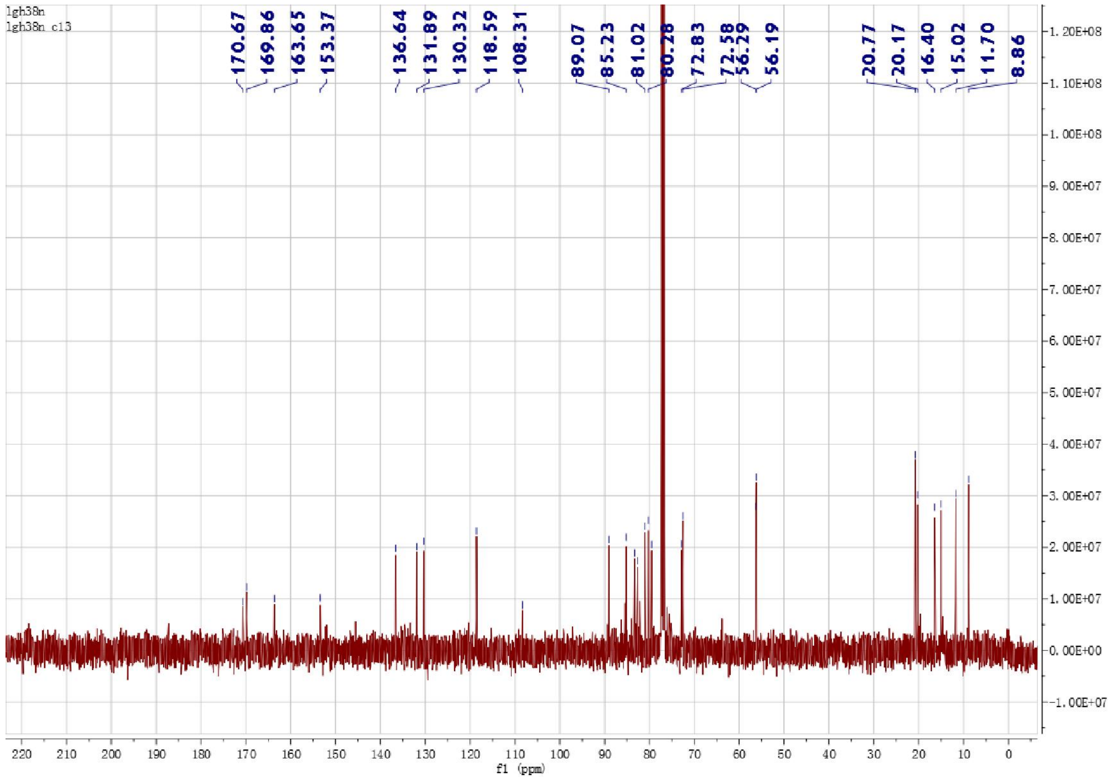

Figure 52S. HSQC of aurovertin Q (**8**).

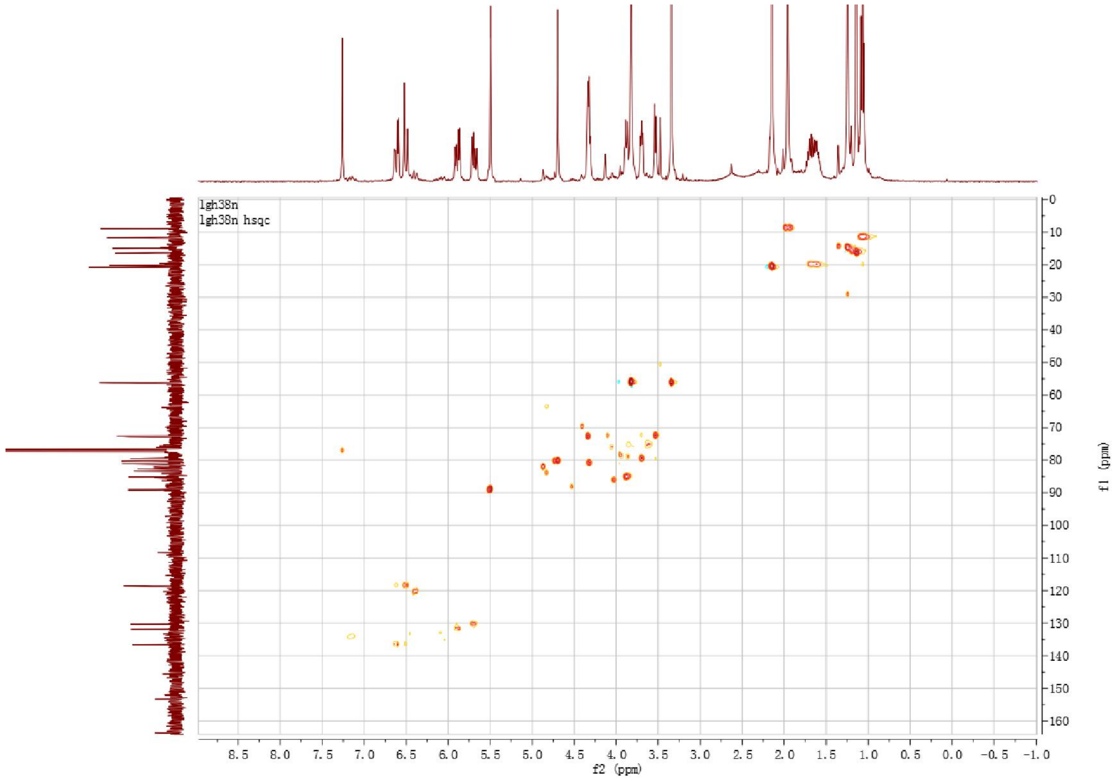

Figure 53S. HMBC of aurovertin Q (**8**).

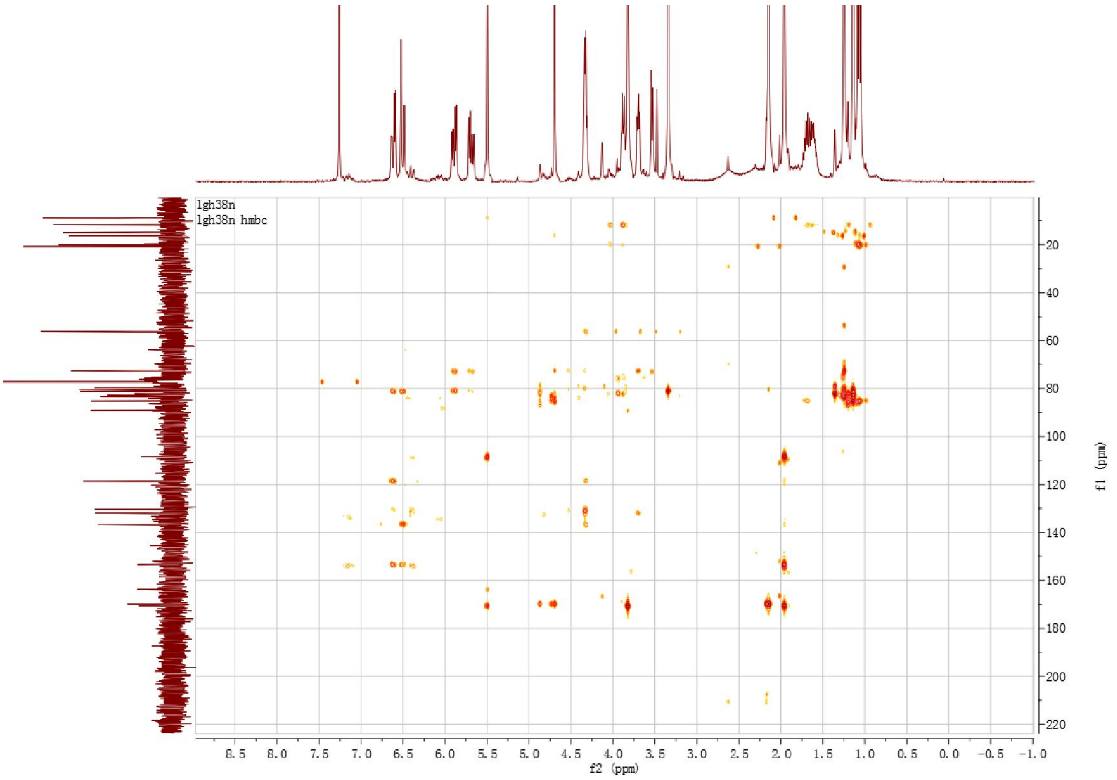

Figure 54S. ^1H-^1H COSY of aurovertin Q (**8**).

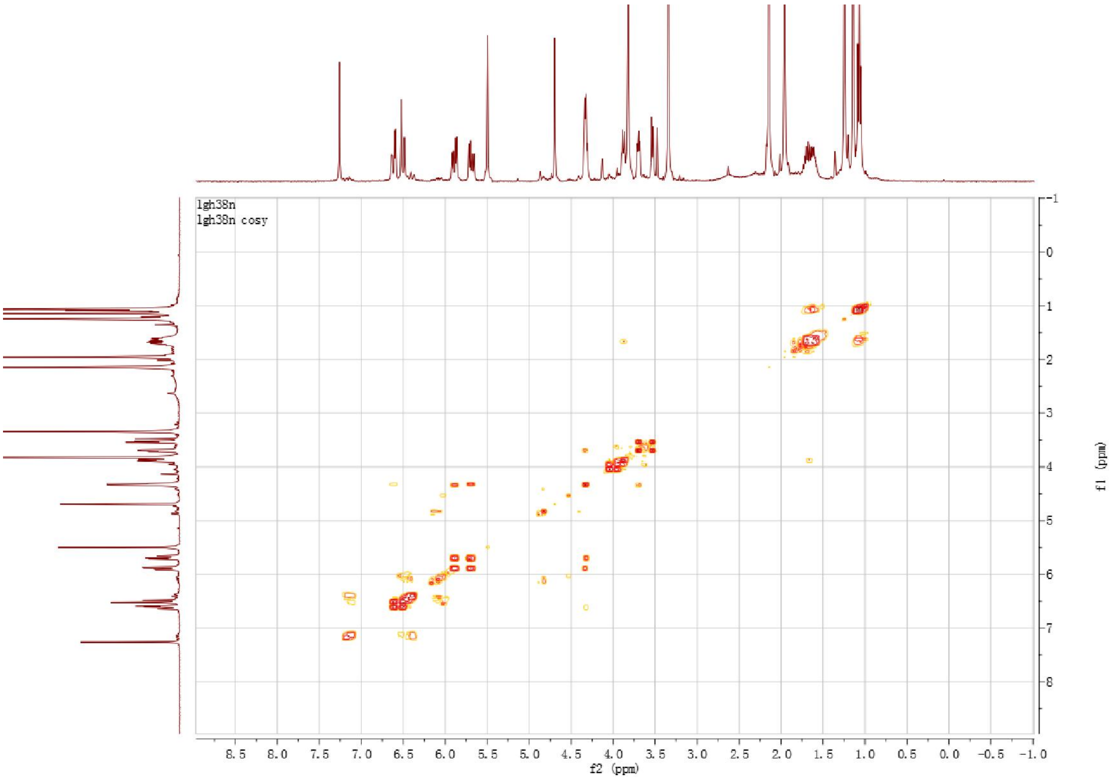

Figure 55S. ROESY of aurovertin Q (**8**).

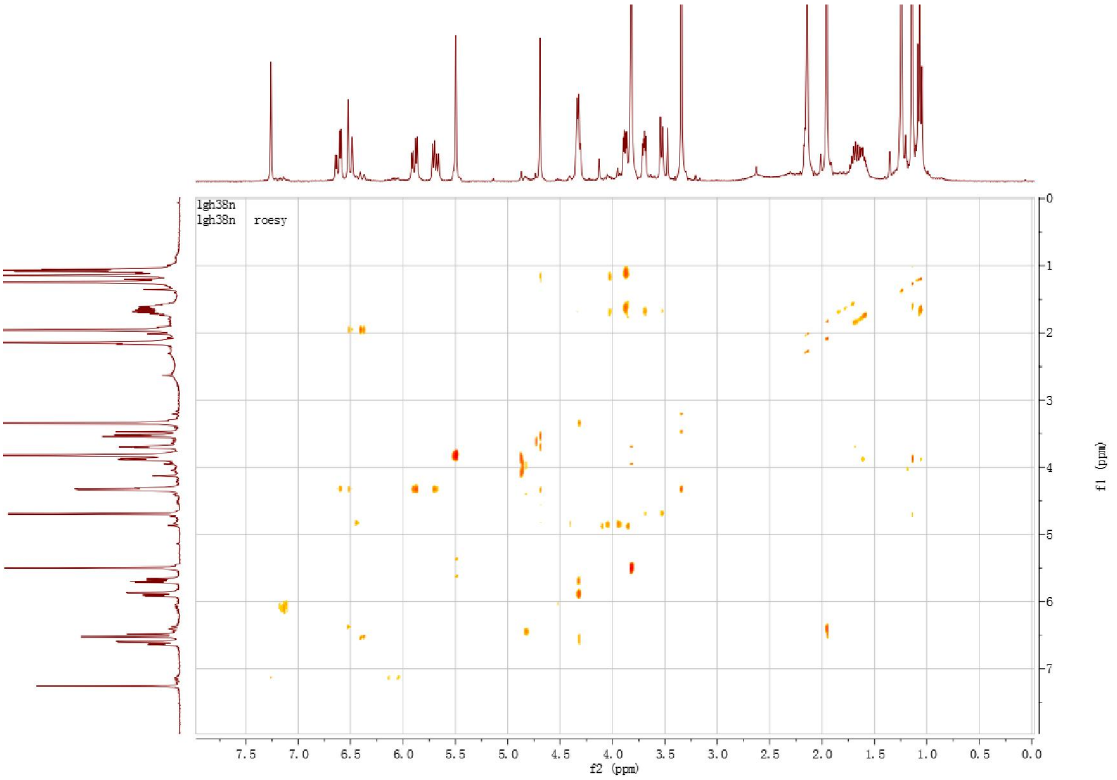

Figure 56S. HREIMS of aurovertin Q (**8**).

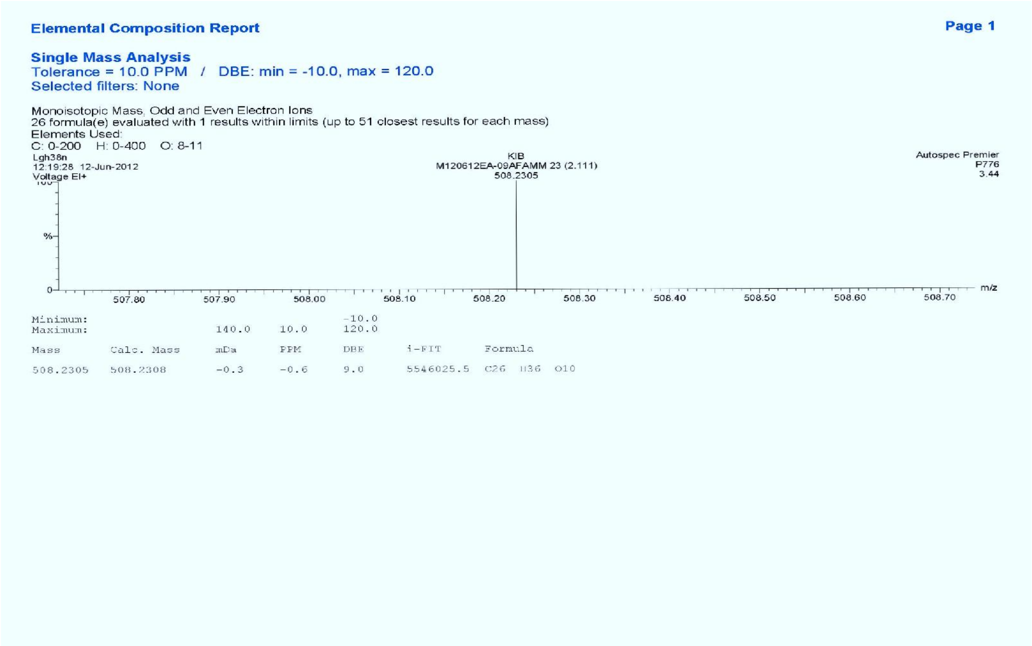

Elemental Composition Report **Page 1**

Single Mass Analysis
Tolerance = 10.0 PPM / DBE: min = -10.0, max = 120.0
Selected filters: None

Monoisotopic Mass, Odd and Even Electron Ions
26 formula(e) evaluated with 1 results within limits (up to 51 closest results for each mass)
Elements Used:
C: 0-200 H: 0-400 O: 8-11

Minimum:				-10.0		
Maximum:		140.0	10.0	120.0		
Mass	Calc. Mass	mDa	PPM	DBE	i-FIT	Formula
508.2305	508.2308	-0.3	-0.6	9.0	5546025.5	C26 H36 O10

Figure 57S. ^{1}H NMR of aurovertin R (**9**).

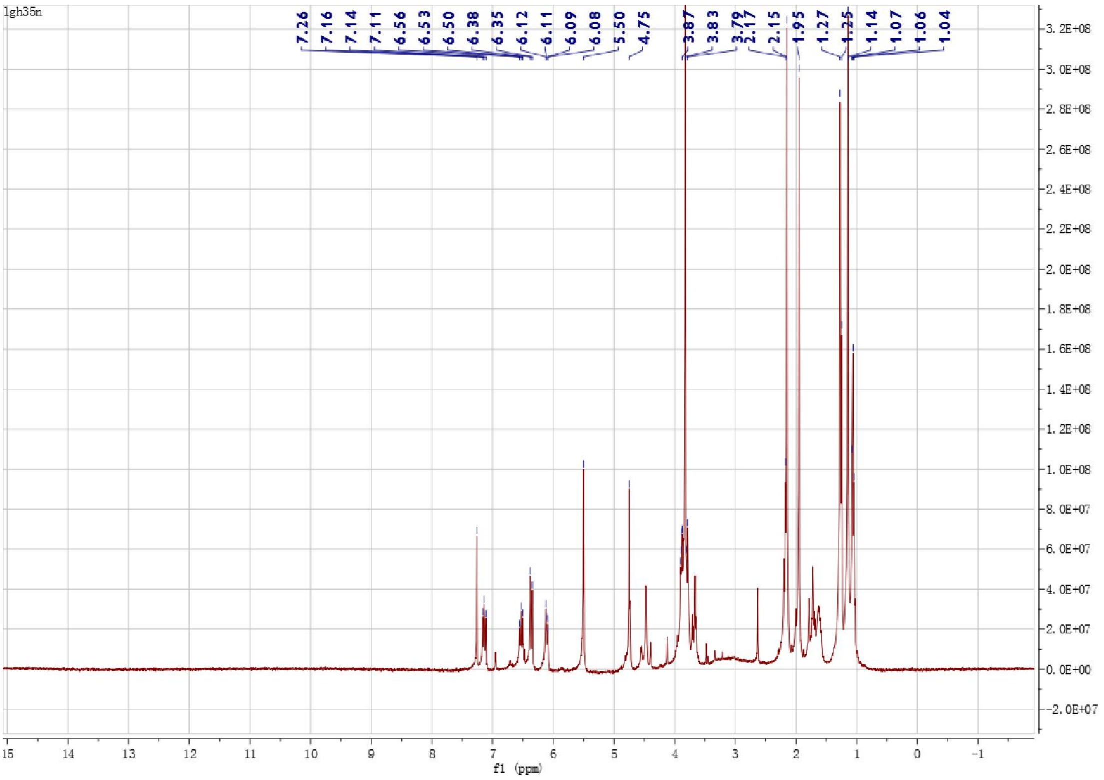

Figure 58S. ^{13}C NMR of aurovertin R (**9**).

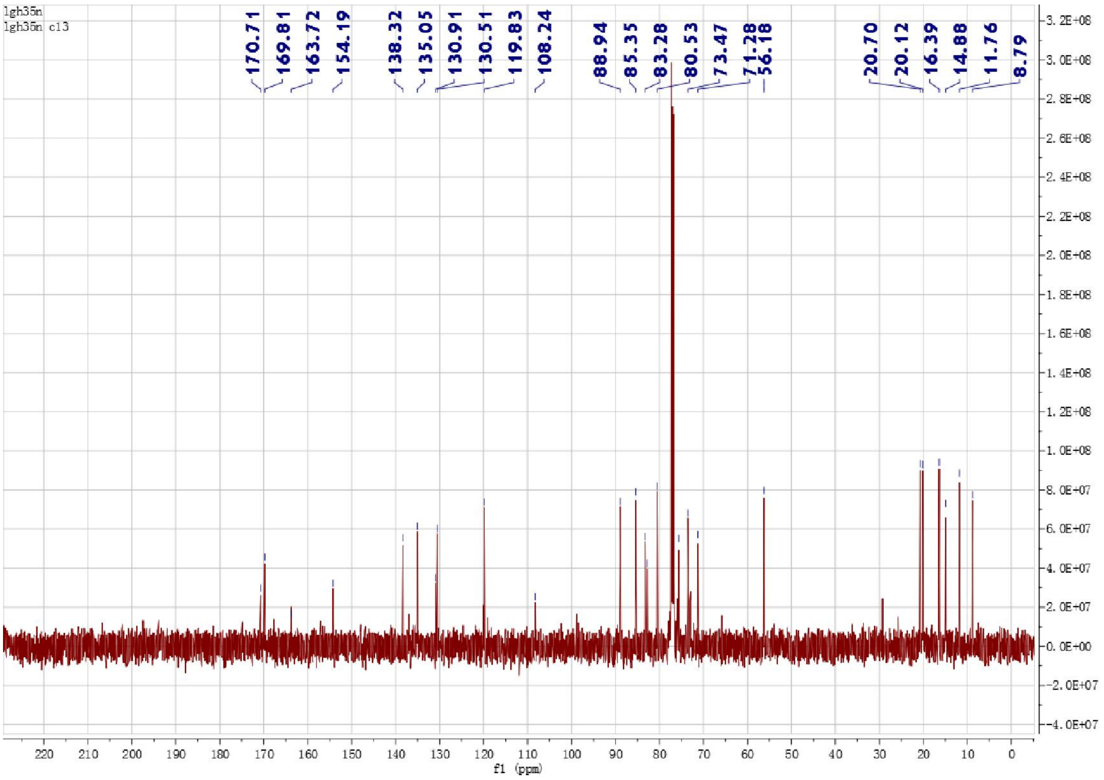

Figure 59S. HSQC of aurovertin R (**9**).

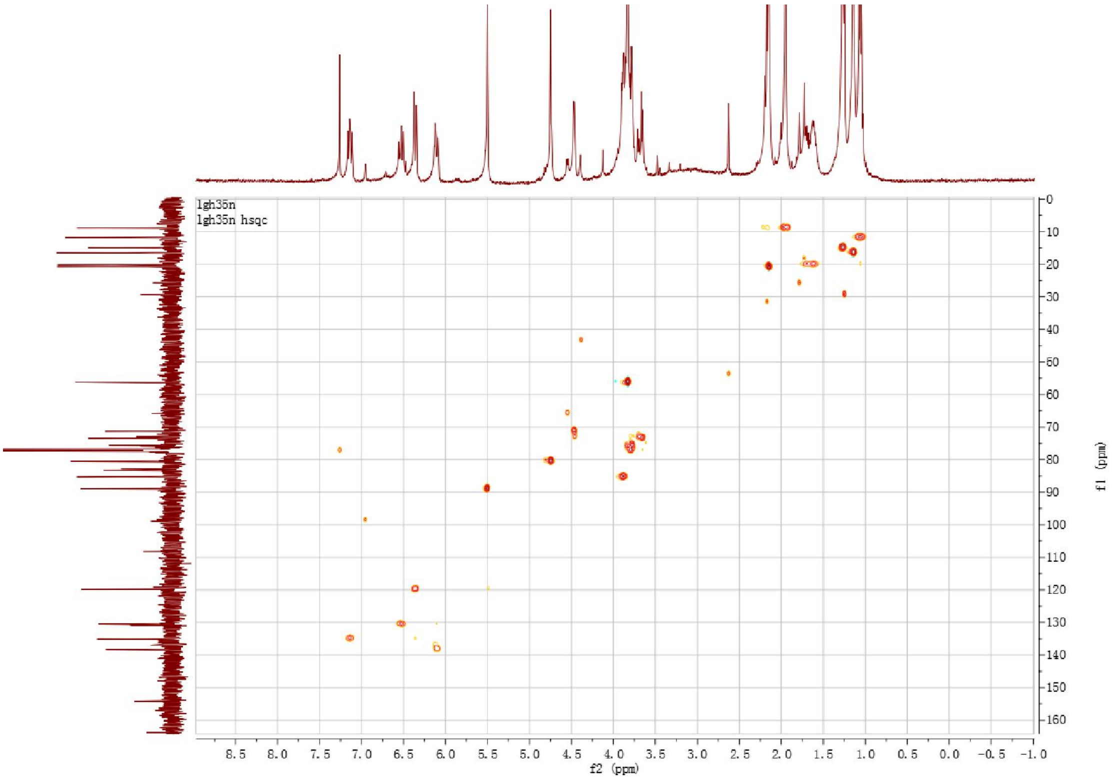

Figure 60S. HMBC aurovertin R (**9**)

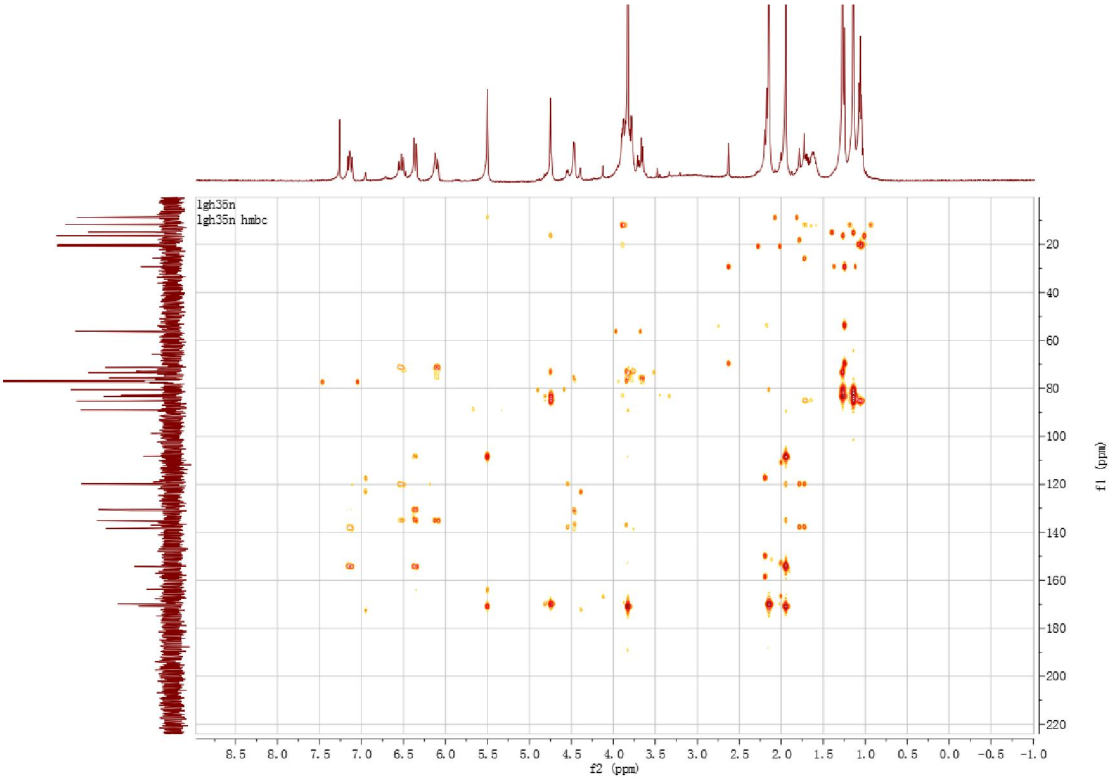

Figure 61S. ^{1}H-^{1}H COSY of aurovertin R (**9**).

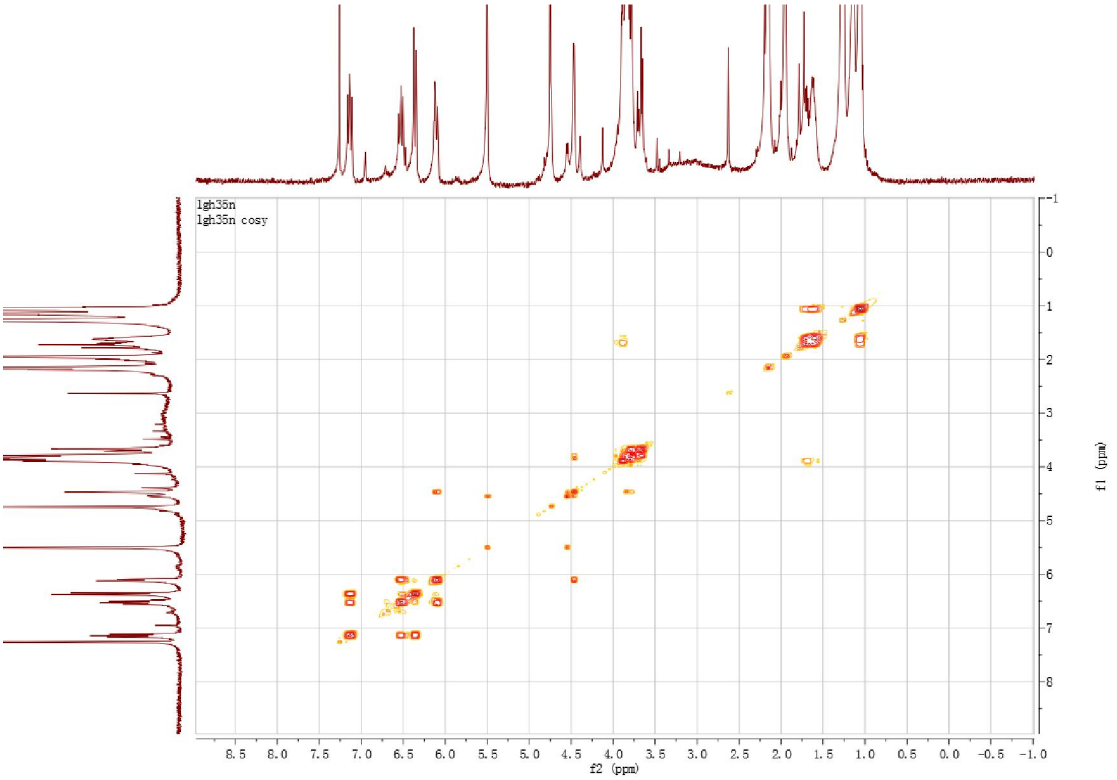

Figure 62S. ROESY of aurovertin R (**9**).

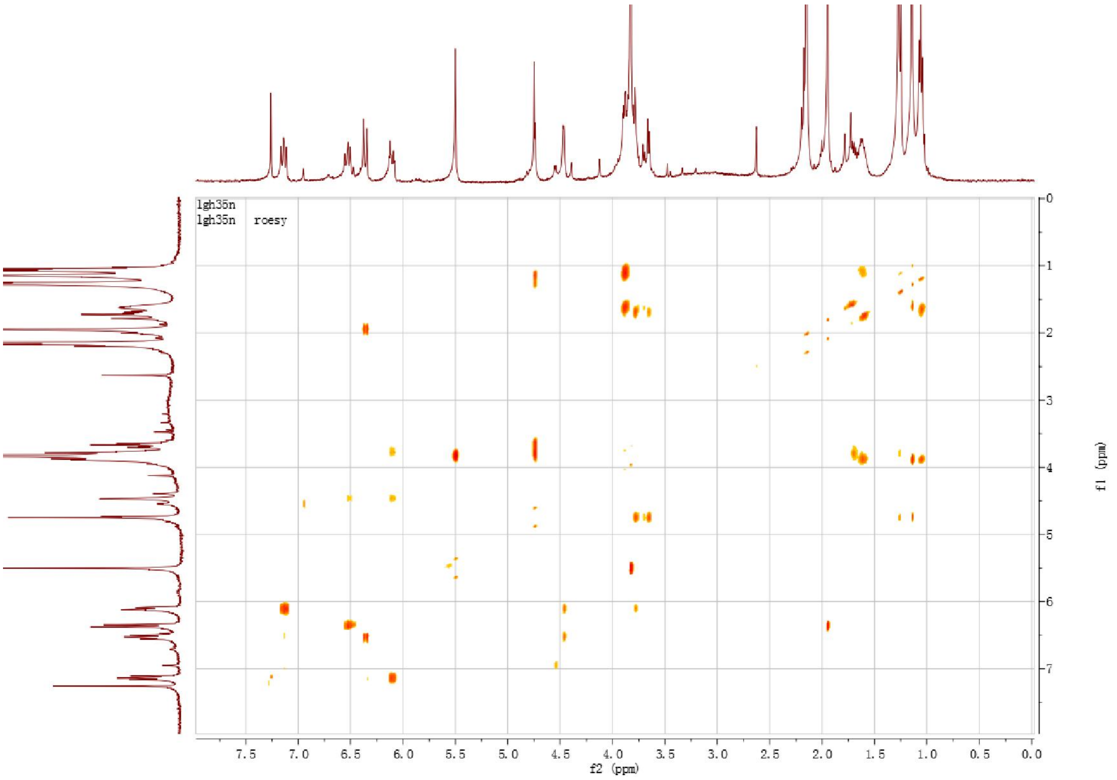

Figure 63S. HREIMS of aurovertin R (**9**).

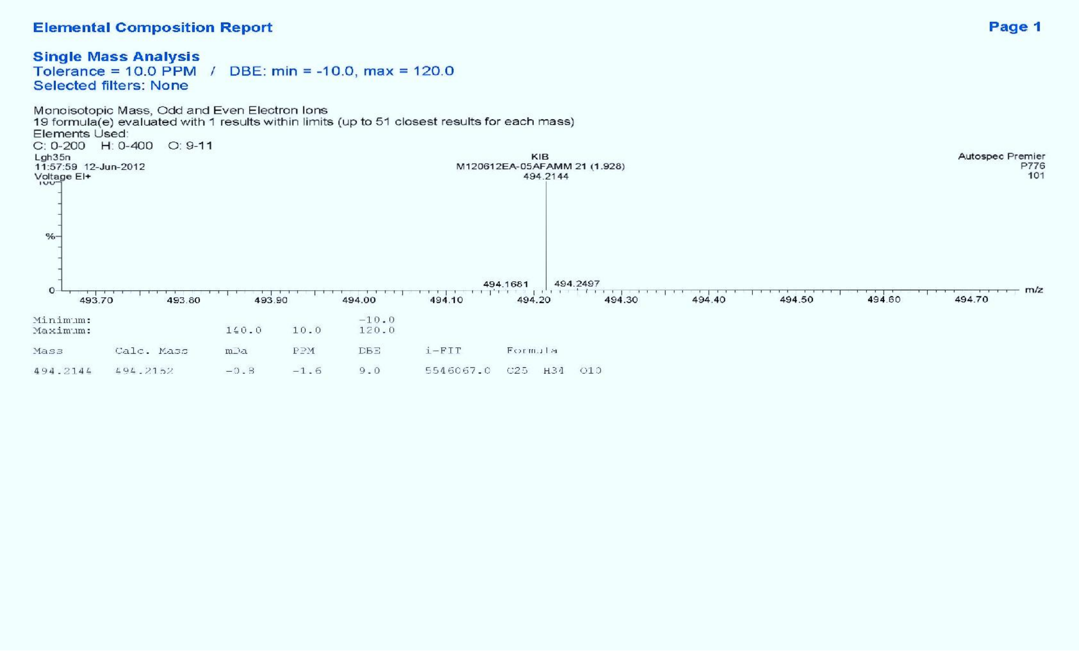

Figure 64S. ^{1}H NMR of aurovertin S (**10**).

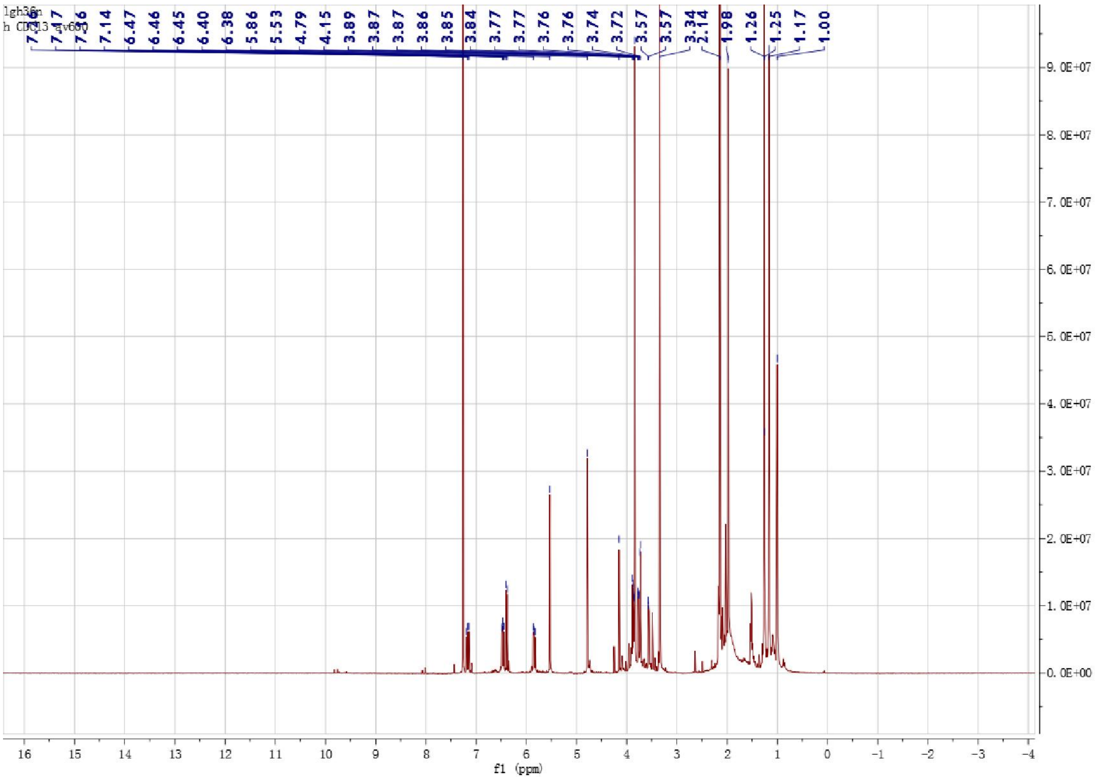

Figure 65S. ^{13}C NMR of aurovertin S (**10**).

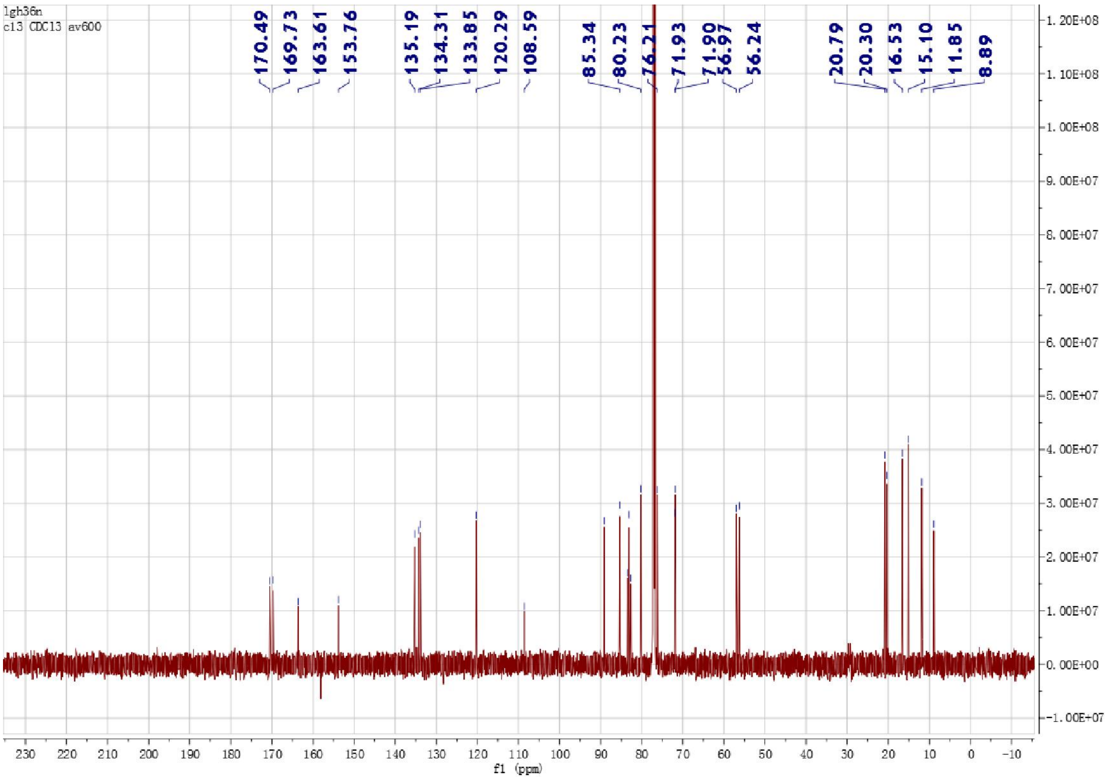

Figure 66S. HSQC of aurovertin S (**10**).

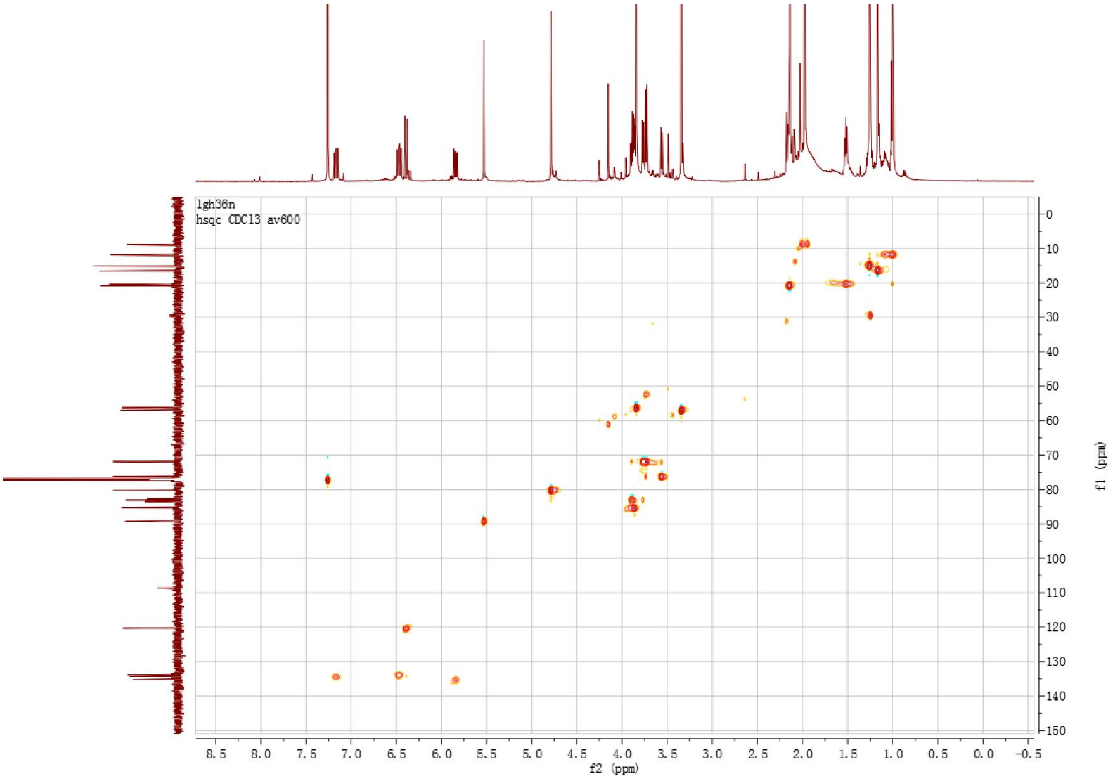

Figure 67S. HMBC aurovertin S (**10**)

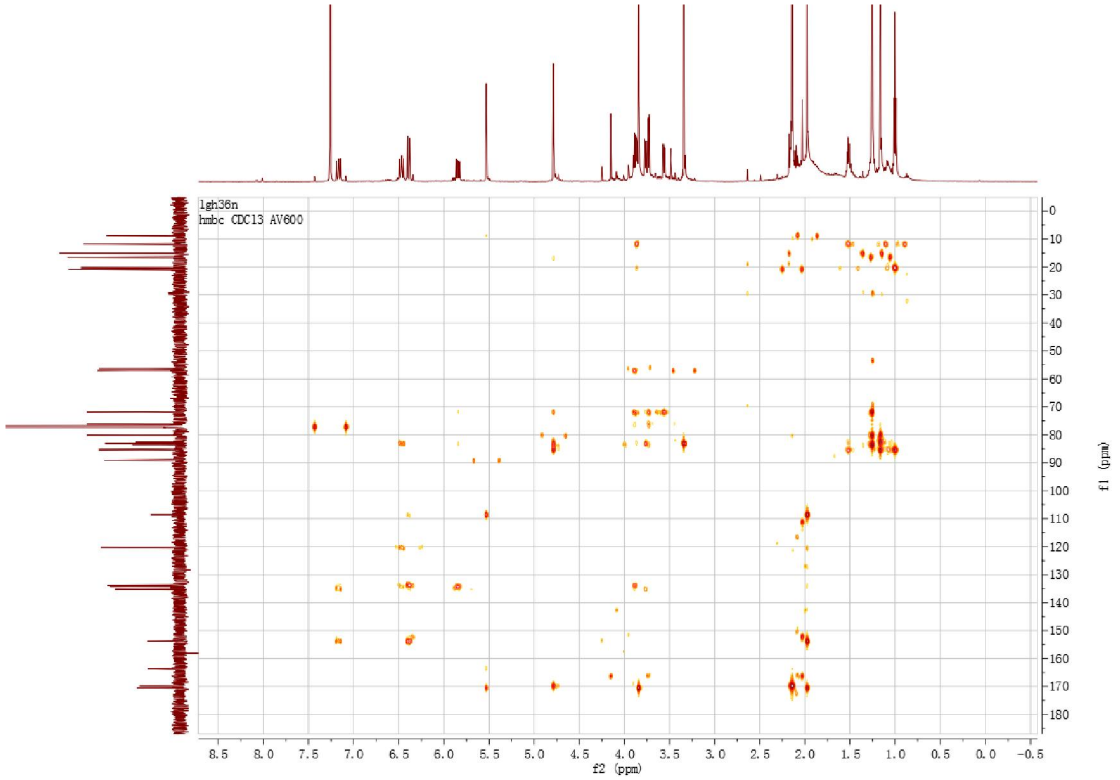

Figure 68S. ^{1}H-^{1}H COSY of aurovertin S (**10**).

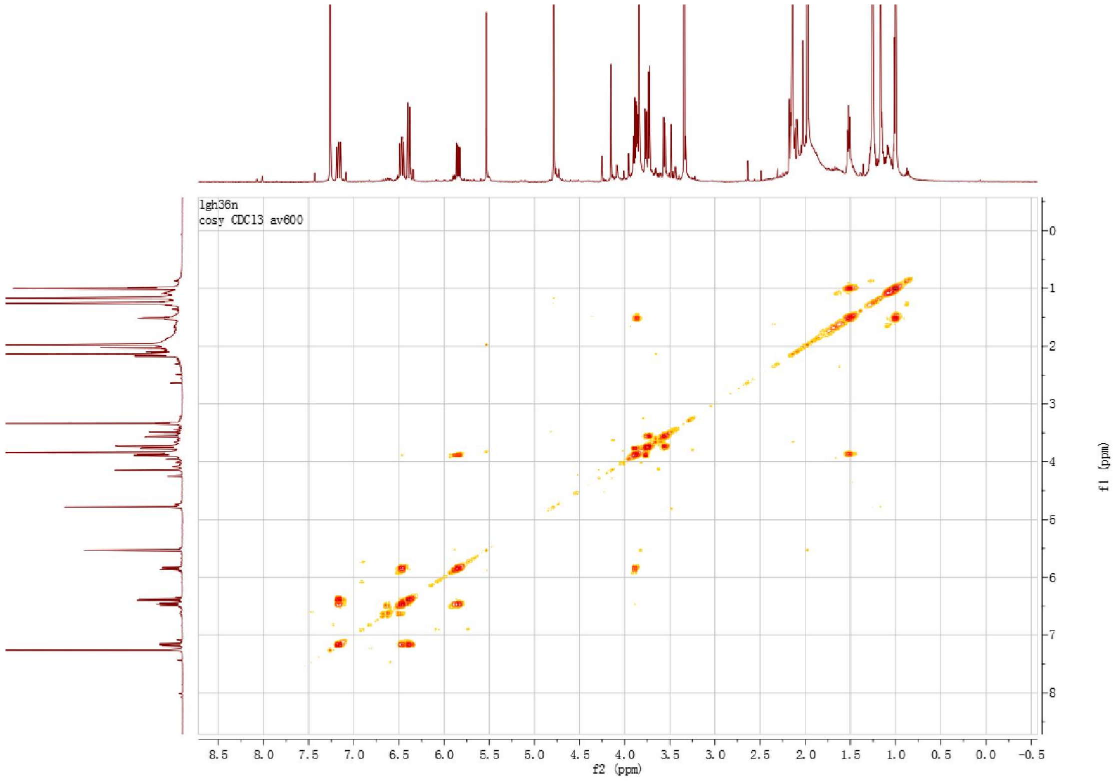

Figure 69S. ROESY of aurovertin S (**10**).

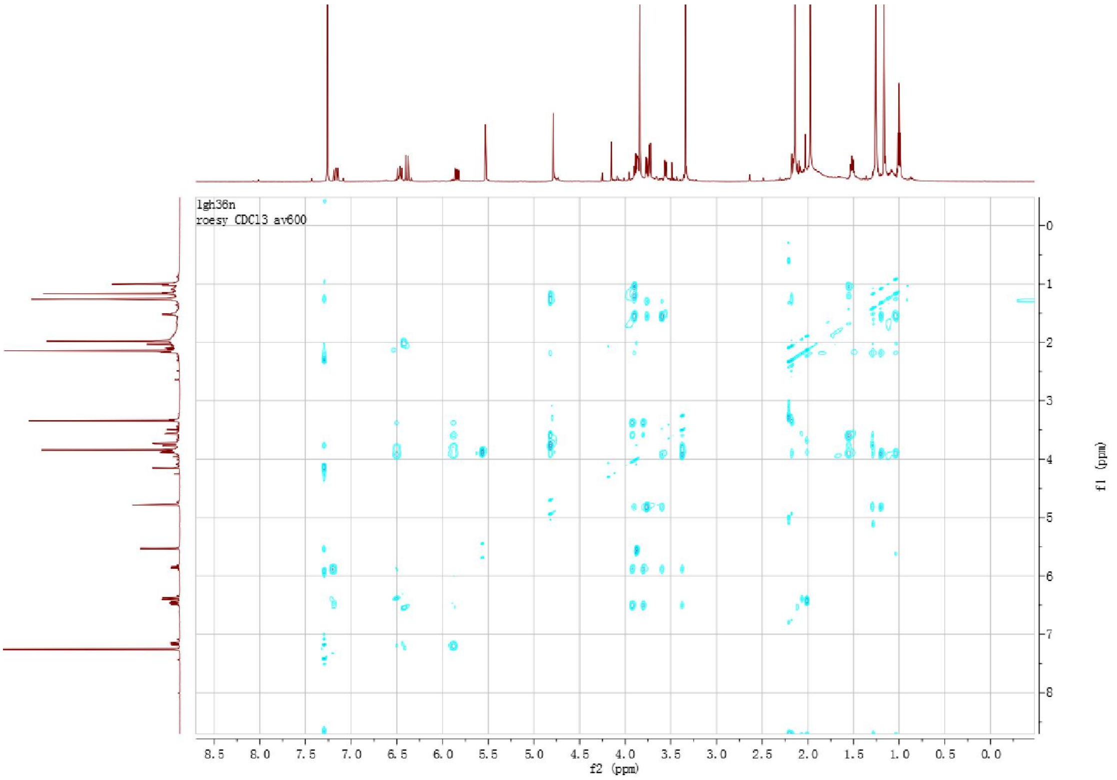

Figure 70S. HREIMS of aurovertin S (**10**).

Elemental Composition Report Page 1

Single Mass Analysis
Tolerance = 10.0 PPM / DBE: min = -10.0, max = 120.0
Selected filters: None

Monoisotopic Mass, Odd and Even Electron Ions
26 formula(e) evaluated with 1 results within limits (up to 51 closest results for each mass)
Elements Used:
C: 0-200 H: 0-400 O: 8-11

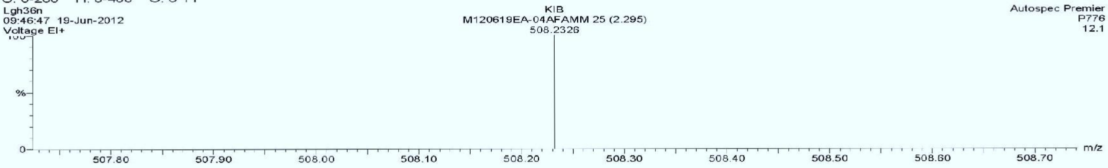

Minimum:				-10.0		
Maximum:		100.0	10.0	120.0		
Mass	Calc. Mass	mDa	PPM	DBE	i-FIT	Formula
508.2326	508.2308	1.8	3.5	9.0	5546027.0	C26 H36 O10
